# Climate urgency enables political alignment for Latin American power integration

**DOI:** 10.1038/s41467-026-77772-x

**Published:** 2026-09-17

**Authors:** Luis Ramírez, Gerardo Blanco, Felipe Sepulveda, Luiz Barroso, Wilfredo C. Flores, Rodrigo Moreno

**Affiliations:** 1 Departamento de Ingeniería Eléctrica, Universidad de Chile, Santiago, Chile; 2 Escuela de Ingeniería Eléctrica, Pontificia Universidad Católica de Valparaíso, Valparaíso, Chile; 3 Departamento de Ingeniería Industrial, Universidad de Chile, Santiago, Chile; 4 PSR, Río de Janeiro, Brasil; 5 Instituto de Investigación Tecnológica, Universidad Pontificia Comillas, Madrid, España; 6 Facultad de Ingeniería, Universidad Tecnológica Centroamericana, Tegucigalpa, Honduras; 7 Facultad de Ingeniería y Ciencias, y Escuela de Negocios, Universidad Adolfo Ibáñez, Santiago, Chile

**Keywords:** Energy and society, Political economy of energy, Climate-change policy, Energy policy

## Abstract

Political fragmentation continues to block urgently needed power integration in Latin America, despite the region’s vast renewable energy potential. Using the Multiple Streams Approach, this study argues that the escalating climate crisis now acts as a focusing event, aligning a pressing energy problem, viable integration solutions, and growing political will. This alignment signals that a policy window is open. This study combines the Multiple Streams Approach with system modeling to quantify overlooked political costs of inaction, specifically, geopolitical tensions and erosion of trust, offering actionable leverage for policy entrepreneurs. Simulations for 2025–2050 show that continued fragmentation increases system costs by up to 7%, reduces resilience to hydroclimatic shocks, and concentrates benefits within narrow cooperating blocs under low-trust conditions. These measurable and rising costs underscore that regional energy integration is no longer just a technical or economic issue; it is a political imperative. Bold leadership must act now to seize this fleeting window of opportunity.

## Abstract (web summary)

Modelling of Latin America’s power systems shows that continued political fragmentation raises costs by up to 7% and weakens resilience to drought, while regional grid integration could save 32 billion USD a year and sharply cut emissions by 2045.

## Introduction

Decarbonization urgency has reached historic levels. In 2024, global temperatures exceeded the 1.5 ^∘^C threshold above preindustrial levels, intensifying climate risks to ecosystems, economies, and communities^1^. As the leading source of greenhouse gas (GHG) emissions globally, accounting for over 70% of total GHG emissions, the energy sector must accelerate the replacement of fossil fuels with clean alternatives to reduce emissions and enhance system resilience^2–8^. Latin America (LA) is well-positioned to lead this transition. In 2023, 62% of its electricity came from renewable sources, well above the global average^9,10^, yet heavy reliance on hydropower ( ~ 45%) exposes the region to hydrological variability and capacity losses projected at up to 17.4% by the century’s end^11,12^, a vulnerability widely recognized in global assessments of climate impacts on energy systems^2^. Indeed, extreme droughts between 2021 and 2025 in Colombia’s Guavio reservoir and across the Rio de la Plata Basin triggered blackouts and rationing^13–17^. Under this context, regional integration, complementing solar in northern Chile, wind in Patagonia, and flexible hydro in the Amazon, offers a technical solution to buffer variability, bolster cross-border resilience, and reduce system costs^9,18–23^.

If the technical case for regional integration is increasingly clear, its political realization has remained far more elusive. Despite decades of recognizing these benefits^24,25^ and numerous initiatives^26–29^, multilateral integration remains stalled^29–31^. While subregional projects like the Central American Electrical Interconnection System (SIEPAC) demonstrate the value of cooperation^32–35^, broader progress continues to be constrained by political and institutional factors^29,36,37^. Governance asymmetries, mistrust, sovereignty concerns, and fragmented regulations, challenges also observed in other regions^33,38–43^, continue to obstruct feasible integration. Global interconnection reviews, in fact, consistently conclude that strong multilateral governance and credible dispute-resolution mechanisms are indispensable for deploying cross-border transmission^41,44–49^. For this reason, understanding how political inertia emerges, and how it can be overcome, remains a critical gap in the literature on cross-border power integration, one that directly shapes energy transitions in Latin America and other emerging regions^36,44,50–60^. To assess the implications of different degrees of cooperation, this study evaluates three integration pathways. Full Integration assumes complete regional coordination in planning, investment, and dispatch across all modeled countries. Partial Integration simulates cooperation among specific sub-regional blocs (e.g., Andean Community, Southern Cone, SIEPAC), reflecting existing political alliances and plausible intermediate steps. Fragmented Systems describe cases where each country optimizes its system primarily on a national basis, with limited cross-border coordination beyond existing agreements. These pathways serve as the analytical backbone of the quantitative scenarios presented throughout this study. In turn, they allow us to quantify the material consequences of the political dynamics that the analytical framework helps interpret, making visible how different degrees of cooperation translate into costs, vulnerabilities, and distributional outcomes across the region.

To navigate these complexities, the Multiple Streams Approach (MSA) offers a comprehensive framework that elucidates the intricate interplay between problems, policies, and political dynamics during the pivotal policymaking process^61^. This approach emphasizes the significance of three distinct streams (problems, policies, and politics) converging at critical junctures, often called “windows of opportunity.” This paper examines Latin American energy integration in the context of the escalating climate crisis and the urgent need for decarbonization, employing the MSA as its analytical framework^61,62^. According to the MSA, policy windows emerge when the problem, policy, and politics streams converge, often triggered by focusing events that heighten political attention^63–66^.

This policy window is open in Latin America. The intensifying impacts of climate change, including hydropower variability, droughts, and regional energy insecurity, act as powerful focusing events, elevating the political salience of energy system resilience and integration^54,67,68^. These events trigger an emerging political will (politics stream), signaled by declarations like the 2023 Brasília Consensus and increased multilateral financing ^56,69–71^.

Integrated modeling presented here shows the political costs of inaction, such as geopolitical and mistrust costs, revealing that fragmentation increases costs by up to 7% and erodes resilience during hydro-shocks, highlighting the tangible stakes. These results offer actionable, evidence-based leverage for policy entrepreneurs^61^ aiming to consolidate political alignment. Thus, these results finally explain why this is a crucial time for advocating for decisive political leadership in Latin America to speed up the energy transition and decarbonization, leveraging the ongoing climate crisis.

## Results

### MSA patterns in the history of Latin American integration

Following independence, Latin American nations largely prioritized national sovereignty, fostering fragmentation even amidst diverse twentieth-century integration efforts^72–74^. In contrast, the energy sector was recognized at an early stage for its critical importance and potential for synergy, emphasizing the ongoing incoherence between national priorities and regional cohesion^75,76^.

Against this backdrop, early country system development was primarily focused on national economies of scale, but in the middle of the century, interest in interconnection grew. The creation of CIER, initially Comisión de Integración Eléctrica Regional and later renamed Comisión de Integración Energética Regional (Regional Energy Integration Commission), in 1964, driven by specific national energy challenges, marked a step, though its foundational congress acknowledged inherent integration difficulties^74,77^. The 1970s oil crises and Cold War dynamics significantly pressured regional economies. In 1973, the Latin American Energy Organization (OLADE) was established to promote cooperation^73,78^; however, action remained primarily bilateral, with large-scale hydropower serving as the practical technical solution at the time (policy stream) ^79^.

This period exemplifies a classic MSA configuration^61^, where convergence of streams was largely driven by exogenous forces. The problem stream (oil crisis) and the politics stream (Cold War geopolitics) were shaped by global dynamics. Coupled with binational hydroelectric policies and enabled by policy entrepreneurs, this opened a policy window that led to landmark bilateral projects such as Itaipú, Salto Grande, and Yacyretá^79–81^. Similar cyclical patterns of cooperation and subsequent stagnation, driven by resource availability and national policy shifts, have characterized other energy integration efforts in the region, such as the Argentina-Chile gas interconnections^82^. However, while the window opened, the policy stream was inherently bilateral, constrained by national interests and focused on managing transboundary resources between specific state pairs^83,84^. This configuration ultimately hindered broader multilateral coordination and reinforced institutional fragmentation^72,74,85^.

By contrast, the SIEPAC case in Central America represents a different MSA pattern, one driven more by endogenous forces. Here, the problem stream was rooted in shared regional vulnerabilities, particularly small, isolated electricity systems lacking scale, resilience, and affordability. The policy stream (SIEPAC itself) was designed explicitly for multilateral implementation, incorporating gradual integration to mitigate sovereignty sensitivities. The politics stream also emerged internally, aligned with regional frameworks like the Central American Integration System (SICA), and reinforced by a shared urgency around supply risks and cost inefficiencies. This convergence allowed for effective multilateral coupling and progress^32^.

A third case, the Initiative for the Integration of Regional Infrastructure in South America (IIRSA), offers a hybrid perspective. Created to promote infrastructure integration across South America, including energy, communications, and transport, IIRSA saw mixed results. Road transport advanced significantly as the streams aligned around a common logistical problem, ready-made technical solutions, and a political consensus around export-led growth, partly driven by exogenous demand from China. Crucially, private-sector actors acted as policy entrepreneurs, steering integration efforts toward global market access rather than intra-regional exchange^86^. Energy integration, however, lacked this alignment. While the problem was acknowledged, the policy stream remained nationally framed, and the politics stream fragmented by sovereignty concerns, stalling progress^73,86,87^.

Understanding these three patterns through the lens of MSA is critical. The initial case shows that exogenous drivers can open policy windows but often lead to bilateral outcomes. The second reveals that shared endogenous vulnerabilities can produce deeper multilateral alignment. And the third illustrates how private entrepreneurial action and external economic drivers can selectively align streams. This comparison is particularly relevant today because, although the climate crisis is exogenous in origin, it generates deeply shared, endogenous vulnerabilities across Latin America, such as water stress, energy insecurity, and economic disruption. These conditions more closely resemble the SIEPAC context than the fragmented geopolitics of the 1970s, suggesting that the current policy window may favor multilateral convergence, if political will aligns accordingly^20,62,63^.

### The problem stream of climate vulnerability and energy insecurity

Despite Latin America’s high renewable electricity share (62% in 2023), its profound reliance on hydropower (4̃5%) creates a critical vulnerability^88^. Climate-driven hydrological variability increasingly compromises energy security across numerous nations, transforming a regional asset into a growing liability^2,88,89^.

Recent climatic events starkly illustrate this escalating risk. Indeed, a prominent paper has found that the current drought in Central Brazil is exceptional in a 700-year context^89^. In 2023, Colombia’s Guavio reservoir dropped to critical levels, jeopardizing national grid stability, while the 2021–2022 drought saw the Paraná River basin in the Southern Cone reach historic lows, severely impacting hydroelectric output and vital navigation routes ^13,14,81^. Such anomalies show increasing links to intensified El Niño-Southern Oscillation (ENSO) patterns, which are projected to become more frequent and severe under continued global warming^90,91^.

Modeling studies project that Latin America’s hydroelectric potential could diminish by 7.5% to 17.4% by the end of the century, contingent on global emissions pathways^2,11,92,93^. These impacts are regionally heterogeneous. Consistent declines are anticipated in Central America, Mexico, and Southern Cone nations (e.g., Argentina, Chile), whereas parts of the Andean region (e.g., Colombia, Peru) might experience stabilization or marginal gains due to altered precipitation patterns^94–96^. Ecuador, despite its Andean location, constitutes an important exception because its hydroelectric system is concentrated in basins particularly sensitive to ENSO-driven variability and projected precipitation reductions in its coastal and inter-Andean watersheds, making it more closely aligned with the vulnerability profile of Southern Cone nations^11,15^. This differential exposure significantly exacerbates risks for highly hydro-dependent countries like Paraguay and Ecuador, and starkly underscores how escalating global emissions intensify regional energy vulnerability in ways that do not follow simple geographic boundaries^12,15,97^.

Within the MSA, the intensifying effects of climate change activate the problem stream. No longer abstract forecasts, energy disruptions now take the form of blackouts, forced rationing, and soaring electricity tariffs across Latin America. As nations scramble for costly alternatives, these obvious impacts elevate public anxiety and political attention, transforming energy resilience from a technical issue into a politically salient priority^12,98^. This shift aligns with Kingdon’s logic that issue salience surges when physical conditions (like climate-induced disruptions) are framed as urgent public problems. Indeed, recent research on drought policy in Brazil demonstrates how such “creeping crises”^62^, initially perceived as regional or cyclical, can escalate to become powerful focusing events that prime national policy agendas when their impacts accumulate and expand. Likewise, as shown in Paraguay’s stagnant energy reforms, climate-induced shocks can prime the policy agenda even in the absence of institutional overhaul, though lasting change depends on political coupling and policy entrepreneurship^83^.

In sum, climate-induced vulnerability and entrenched hydropower dependence have converged to frame a systemic regional risk. This convergence has sharply focused public and institutional attention on Latin America’s energy fragility, solidly activating the problem stream within the MSA. The urgency and visibility of these risks heighten the demand for credible, practical solutions. In this context, a range of technically viable and economically sound integration proposals has emerged, suggesting that the policy stream, comprising feasible, acceptable, and implementable alternatives, is also taking form. The following section examines how regional integration, supported by model-based evidence, offers a compelling response to the systemic energy vulnerabilities now facing the region^84^.

### The Policy Stream and the Quantified Value of Regional Integration Amidst Climate Change

Solutions to societal problems often germinate within specialist communities (Kingdon’s “policy primeval soup”^61^) long before capturing widespread political attention. Regional electricity integration in Latin America exemplifies this pattern because, despite numerous studies highlighting its potential benefits for sustainability, security, and efficiency^19,56,99–101^, its implementation remains uneven, particularly in South America compared to Central America’s SIEPAC experience^32,37,56,102^. While various factors explain this gap, the shared vulnerability to climate change is emerging as a potentially unifying driver demanding regional cooperation^21,68,103^.

This study contributes directly to this policy stream by rigorously quantifying the benefits of integration using an optimal power system planning model tailored for Latin America’s context (see Methods section for model details). The model co-optimizes investments in generation, storage, and cross-border transmission infrastructure (2025–2050) to minimize total system costs (operational expenditures (OPEX), capital expenditures (CAPEX), and carbon costs) while meeting national demands and decarbonization goals, explicitly leveraging the complementarity of the region’s diverse renewable resources under various hydrological scenarios.

The following results summarize how this policy alternative performs across the main dimensions of decarbonization, cost, investment, and resilience.

### Quantitative benefits of regional integration

The simulations contrast integrated scenarios against fragmented, status-quo national systems that retain only currently existing interconnections, revealing substantial benefits achievable through coordinated regional action.

**Fig. 1:**
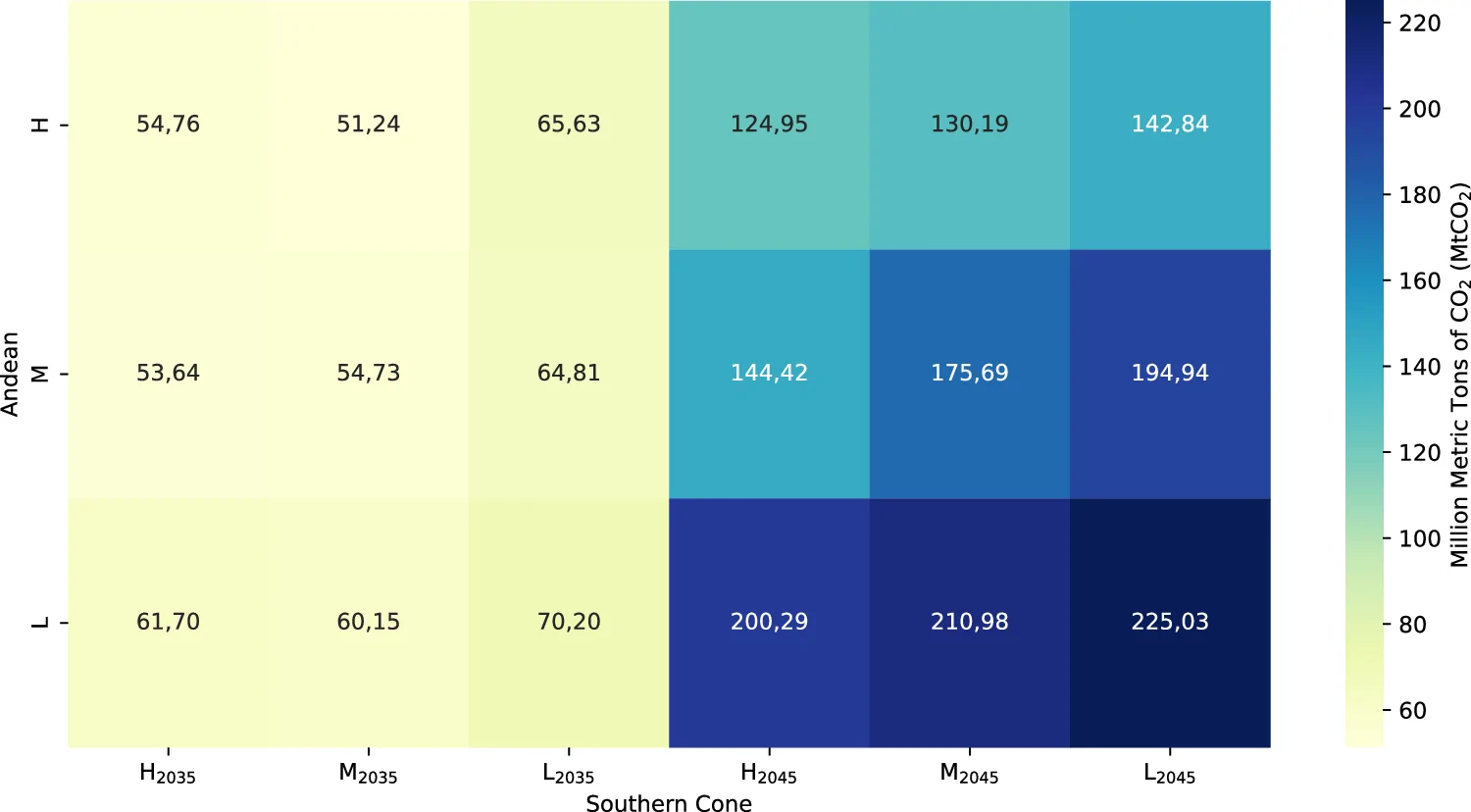
Regional power integration significantly curtails CO_2_ emissions from Latin America’s power sector, enhancing decarbonization efforts by 2045. This figure quantifies the annual reduction in CO_2_ emissions (MtCO_2_ yr^−^^1^) achieved through optimized regional electricity interconnection relative to a counterfactual scenario in which national power systems remain limited to today’s level of cross-border interconnection, with no additional transmission investment. Emissions data, presented for the year 2045, are disaggregated across nine distinct hydrological scenarios, reflecting a 3 × 3 matrix of High (H), Medium (M), and Low (L) hydropower availability in the Andean (rows) and Southern Cone (columns) regions (see Methods and Supplementary Materials for detailed scenario definitions and absolute emission figures). The substantially greater avoided emissions under low-hydropower conditions particularly underscore the critical role of integration in building resilient, low-carbon energy systems capable of meeting climate targets even under hydrological stress.

Emissions reductions are a central benefit of integration because optimized regional interconnection significantly curtails power sector carbon dioxide (CO_2_) emissions. By 2045, integrated scenarios achieve emissions levels between 41 and 99 million tonnes of CO_2_ (MtCO_2_) annually, drastically lower than the 174 to 324 MtCO_2_ projected without additional interconnections, especially under challenging low-hydropower conditions (Supplementary Table 6). These results demonstrate a deeper decarbonization potential compared to scenarios like the Smart Grid Scenario (SGS) from De Oliveira-De Jesus et al.^104^ and align with reduction potentials identified by Kober et al.^23^. The avoided emissions, visualized in Fig. 1, underscore integration’s role as a key climate mitigation tool.

**Fig. 2:**
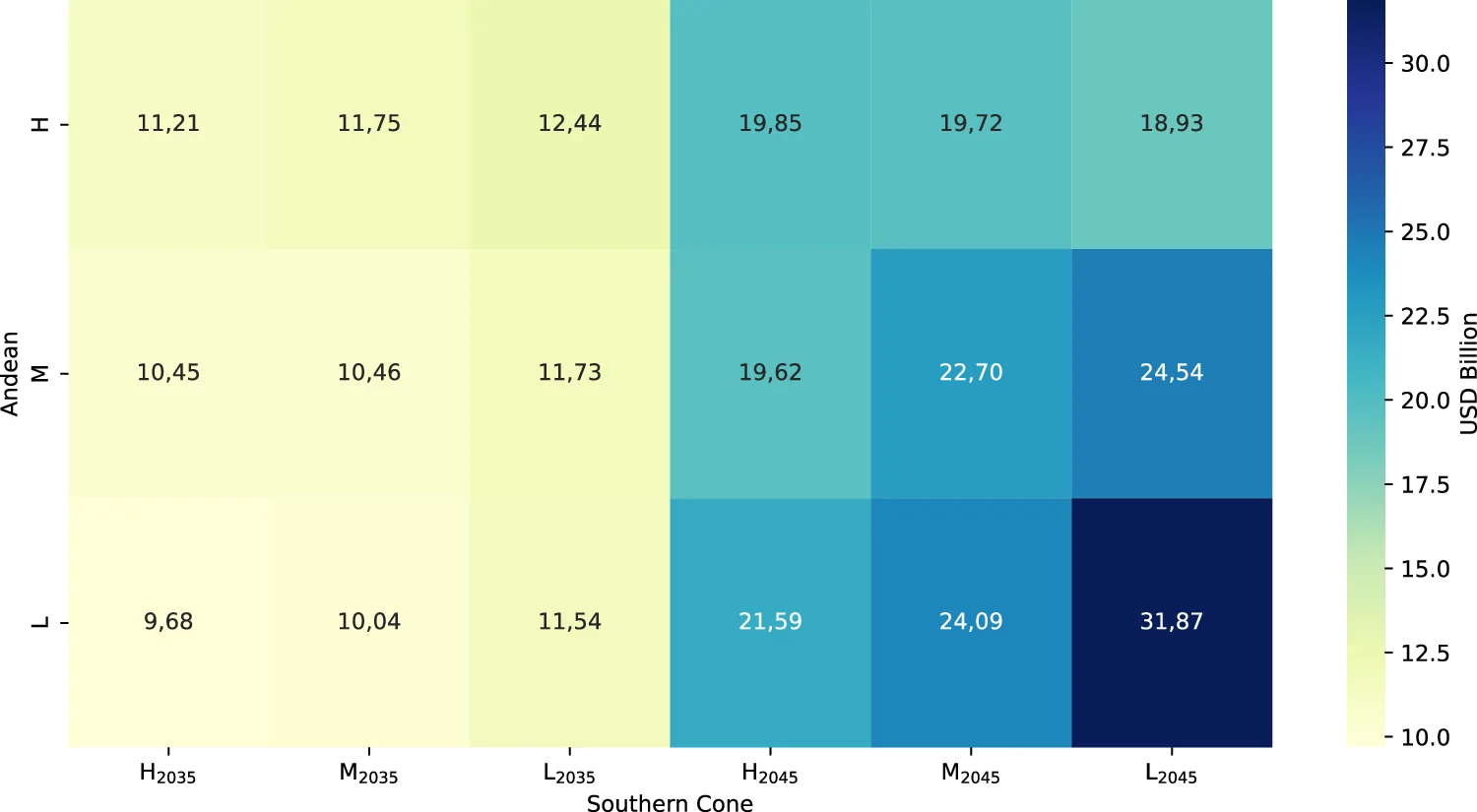
Substantial economic savings are unlocked through regional power integration in Latin America by 2045. The figure illustrates the net annual economic benefits (billion USD yr^−^^1^) derived from optimized regional power system interconnection compared to the higher costs associated with fragmented national systems that retain only current levels of cross-border interconnection. These savings are projected for the year 2045 across a 3 × 3 matrix of nine hydrological scenarios, encompassing High (H), Medium (M), and Low (L) hydropower availability in both the Andean (rows) and Southern Cone (columns) regions (refer to Methods and Supplementary Table 7 for detailed scenario parameters and absolute system cost data). The results highlight the compelling economic advantages of a coordinated regional approach, with particularly pronounced savings achieved during periods of significant water scarcity, thereby enhancing economic resilience against climate-induced shocks.

Integration also lowers system costs by yielding compelling economic advantages through efficient resource allocation. By 2045, total system costs in integrated scenarios range from 106 to 130 billion USD annually, compared to 126 to 162 billion USD for fragmented systems without additional cross-border transmission investment (Supplementary Table 7). This translates into potential net economic savings (avoided losses) reaching up to 32 billion USD annually by that year, particularly significant during periods of water scarcity (Fig. 2). These findings are notably consistent with savings projected by Barbosa et al.^105^, reinforcing the economic rationale for interconnection.

**Fig. 3:**
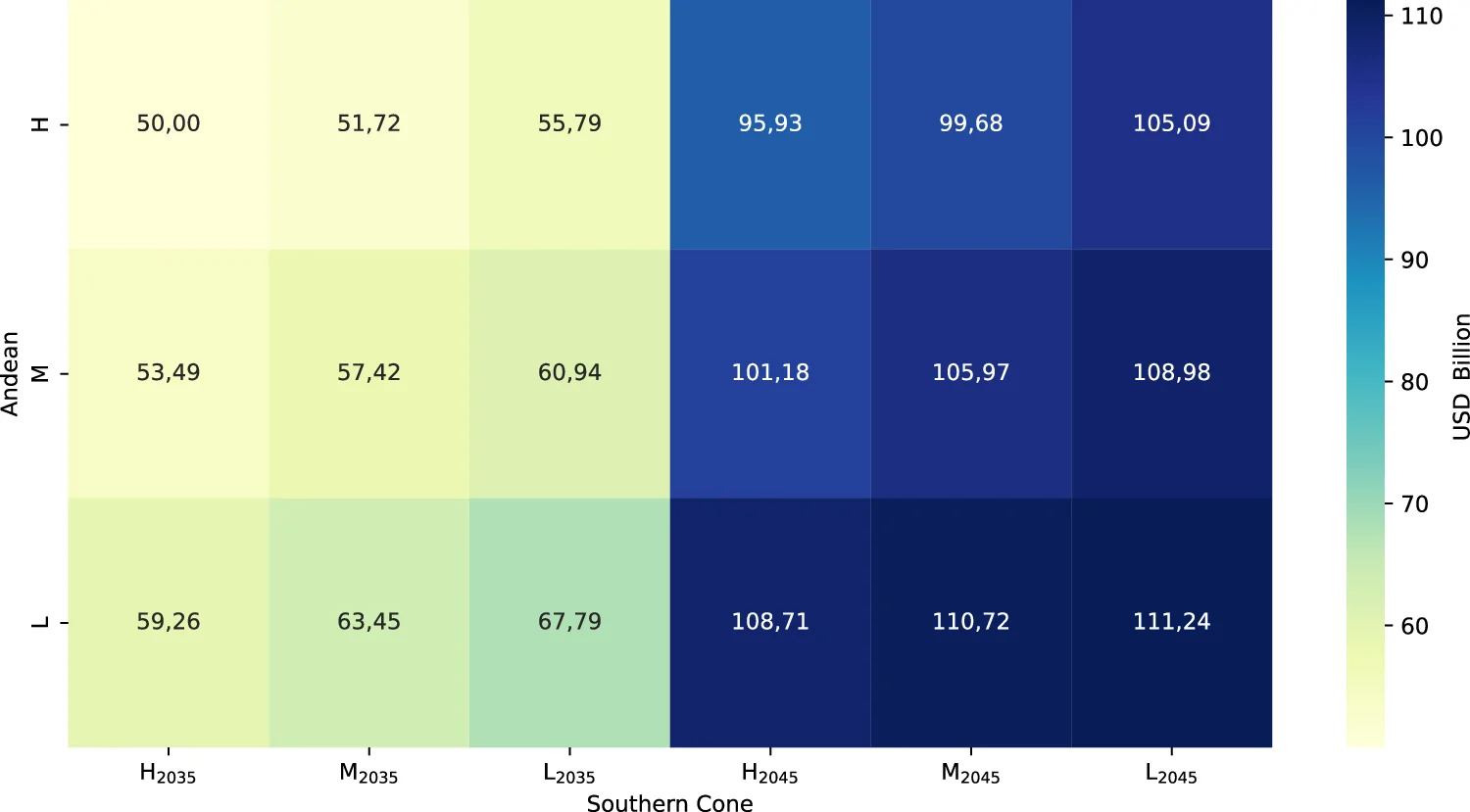
Regional integration facilitates optimized and efficient capital investment for Latin America’s power sector decarbonization by 2045. This figure presents the projected annual capital expenditure (billion USD yr^−^^1^) required for generation, storage, and cross-border transmission infrastructure under the optimized regional integration scenarios for the year 2045. Investment levels are detailed across nine distinct hydrological scenarios (a 3 × 3 matrix of High (H), Medium (M), and Low (L) hydropower availability in the Andean and Southern Cone regions; see Methods for comprehensive modeling assumptions). While substantial, these investments represent an efficient allocation of capital across the region, made possible by integration, which is crucial for achieving ambitious decarbonization goals through the optimal deployment of VRE sources and the strategic sharing of power resources.

Integration also optimizes investment because, while decarbonization requires substantial capital investment (reaching 96–111 billion USD by 2045 in the integrated scenarios, Fig. 3), integration ensures this capital is deployed more efficiently across the region. These investment levels are consistent with projections from ambitious climate scenarios in studies like Kober et al.^23^ and recent International Energy Agency (IEA)^106^ estimates for renewable energy needs. Furthermore, these findings align quantitatively with very recent sub-regional analyses. For instance, Wessel et al. (2025)^107^ demonstrate that uncoordinated decarbonization in the Southern Common Market (Mercosur) region alone increases additional generation capacity investments from 3.5–7.0 billion USD to 14.7–22.8 billion USD (a 3x to 5x increase). This magnitude of inefficiency is consistent with these findings, confirming that while the integrated pathways require 50–111 billion USD across 21 countries, political fragmentation would drive these regional capital requirements dramatically higher, scaling to a penalty far greater than that observed for the Mercosur bloc alone. Integration thus facilitates optimal use of variable renewable energy sources (VREs), channeling investments effectively while preventing massive capital waste.

Integration also enhances resilience and complementarity, an urgent priority given the diverse and significant climate change impacts projected for energy systems globally and regionally^2^. Regional interconnection is widely recognized as a powerful instrument for managing climate-induced hydrological risk, leveraging the strategic complementarity of diverse energy resources^12,18,88^. The model explicitly quantifies how interconnected Latin American systems can harness these geographic and technological synergies, balancing variable solar and wind generation across regions while compensating for hydropower deficits during droughts (as modeled for the Andean and Southern Cone regions) through imports from neighbors. This approach provides crucial levels of resilience and flexibility often unattainable within fragmented national systems, limited to today’s level of interconnection, that are heavily dependent on variable hydro resources.

The quantitative results from this analysis provide robust, multi-faceted evidence for the technical viability and socio-economic desirability of enhanced regional electricity integration as a core strategy for Latin America’s energy transition. Visualizations derived from the model (Figs. 1–3) offer concrete tools to aid policymakers in understanding trade-offs and benefits. A strong “policy stream”, rich in data, supported by consistent findings across multiple studies (including this one), and featuring well-defined, technically sound solutions, clearly exists. The critical bottleneck, therefore, lies not in the absence or weakness of viable policy options but in achieving the political consensus and institutional coordination necessary for their implementation, a challenge explored next. Indeed, similar compelling economic and security benefits are increasingly recognized for other forms of energy integration within Latin America, such as natural gas interconnections, highlighting a broader regional potential for cooperative energy strategies ^82^. Beyond energy security and emissions reductions, regional integration also carries significant implications for international economic competitiveness. OLADE (renamed the Latin American and Caribbean Energy Organization, OLACDE, in December 2025) has advanced the concept of *powershoring*, whereby Latin America’s abundant and low-cost renewable energy potential becomes a structural comparative advantage for attracting energy-intensive industries and green hydrogen value chains, as part of its analysis of regional electricity integration ^108^. Integrating power systems at the regional scale would amplify this advantage by enabling more stable, affordable, and reliable electricity supply across borders, reinforcing the region’s positioning in global decarbonized supply chains. This international competitiveness dimension adds a further layer to the cost argument for integration because, beyond avoiding internal system inefficiencies, fragmentation also forfeits the opportunity to leverage collective renewable endowments as a geopolitical and economic asset in the global energy transition.

### The politics stream and the quantified costs of fragmentation

Public policies emerge from complex political negotiations, not solely rational planning ^61^. In Latin America, this ‘politics stream’ is particularly challenging for energy integration, often stalled by dynamics like strong interest groups, sovereignty concerns, and historical distrust that favor national interests over collective action^74,83,84^. This political landscape is further shaped by contemporary geopolitical forces, both endogenous and exogenous, that simultaneously create additional pressures and opportunities for regional cooperation^87,109–111^. On the one hand, the global geopolitics of the energy transition has increased Latin America’s strategic relevance, given the region’s renewable resource base and its role in critical mineral supply chains for low-carbon technologies, including lithium, copper, nickel, graphite, and rare earth elements^109,110,112,113^. These dynamics may create incentives for deeper intra-regional coordination, not only to reduce system costs and improve resilience, but also to strengthen Latin America’s collective bargaining capacity in global decarbonized value chains. Similarly, European Union–Latin America and the Caribbean (EU-LAC) climate cooperation and agreements such as European Union–Mercosur (EU-Mercosur) and European Union–Chile (EU-Chile) provide exogenous momentum by introducing opportunities for financing, regulatory learning, and institutional convergence that could accelerate integration governance^114–117^. Concurrently, the renewed projection of United States (US) strategic primacy over the Western Hemisphere, articulated in the recent US National Security Strategy as a contemporary corollary to the Monroe doctrine, constitutes a further exogenous pressure that, while heightening external uncertainty, may paradoxically reinforce intra-Latin American cooperation as a defensive strategy to safeguard regional autonomy and decision-making over its energy resources and critical mineral wealth^109,110,118^. On the other hand, electoral shifts and energy-policy divergences in countries such as Argentina, Chile, Bolivia, and Ecuador risk reinvigorating resource nationalism and sovereignty-centered approaches, potentially counteracting integration efforts^109–111^. These competing geopolitical currents underscore that the politics stream is not static, and that policy entrepreneurs must navigate an increasingly complex international environment. While previous studies have quantified the economic benefits of regional power integration^23,56,105^, this study advances this literature by explicitly nuancing the geopolitical dimension of trust and mistrust into the analysis. Using a detailed energy system model, the analysis quantifies the economic costs not only of non-cooperation in general but also of political fragmentation driven by historical distrust and sovereignty concerns, barriers also consistently identified by regional institutions analyzing the slow pace of energy cooperation^108^. This framing translates abstract political dynamics into concrete, comparable trade-offs, offering policymakers a clearer understanding of how trust (or the lack thereof) materially shapes the regional energy landscape and the potential returns of overcoming these barriers.

### Quantified costs of political factors

The analysis operationalizes and quantifies key political costs.

**Fig. 4:**
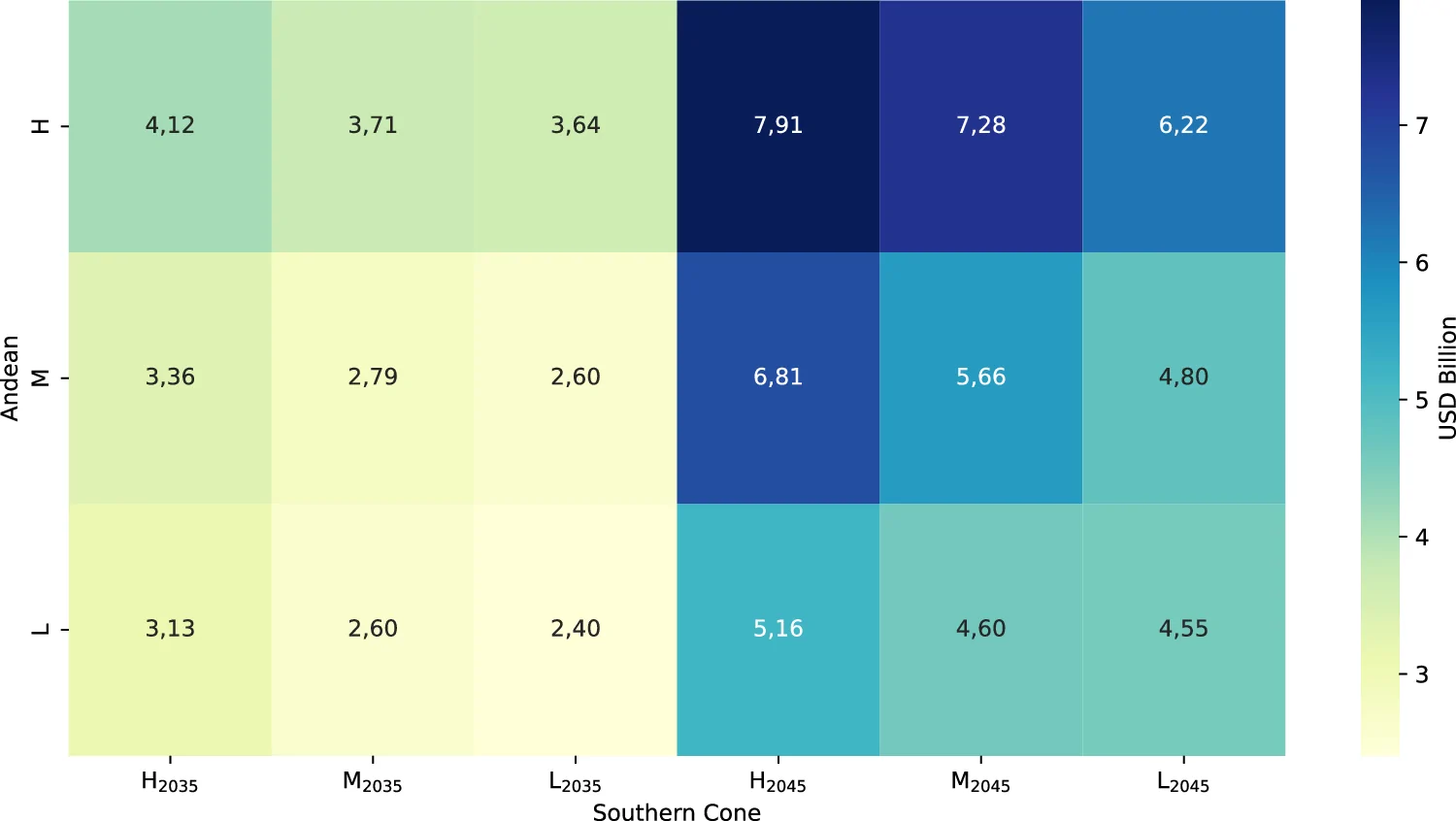
The geopolitical cost of prioritizing energy sovereignty over regional cooperation. Heat maps illustrate the estimated annual `Geopolitical Cost' across Latin America for 2035 and 2045, under scenarios with optimized transmission investments. This cost, calculated as the difference in total system cost between scenarios with and without national energy sovereignty constraints (see Methods), represents the economic inefficiency (in billions USD) imposed by prioritizing national self-sufficiency over fully optimized regional synergy. Results are shown across a 3 × 3 matrix combining High (H), Medium (M), and Low (L) hydropower availability scenarios for the Andean (rows) and Southern Cone (columns) regions. Notably, costs increase significantly by 2045 due to higher demand and can be counter-intuitively higher under abundant hydropower, as this reduces the immediate economic pressure for optimal cross-border exchange, thus magnifying the inefficiency cost of the sovereignty constraint. Costs range from 2.40 to 4.13 billion USD (2035) and from 4.55 to 7.91 billion USD (2045).

The ‘Geopolitical Cost’ of sovereignty preference is estimated as the economic penalty incurred when national sovereignty constraints hinder optimal regional cooperation (see Methods; Fig. 4). This cost reveals a significant inefficiency, amounting to several billion USD annually (4.6–7.9 billion USD by 2045 with integration), driven by suboptimal system deployment when national priorities are prioritized over regional synergy. Detailed dynamics are presented in Fig. 4.

**Fig. 5:**
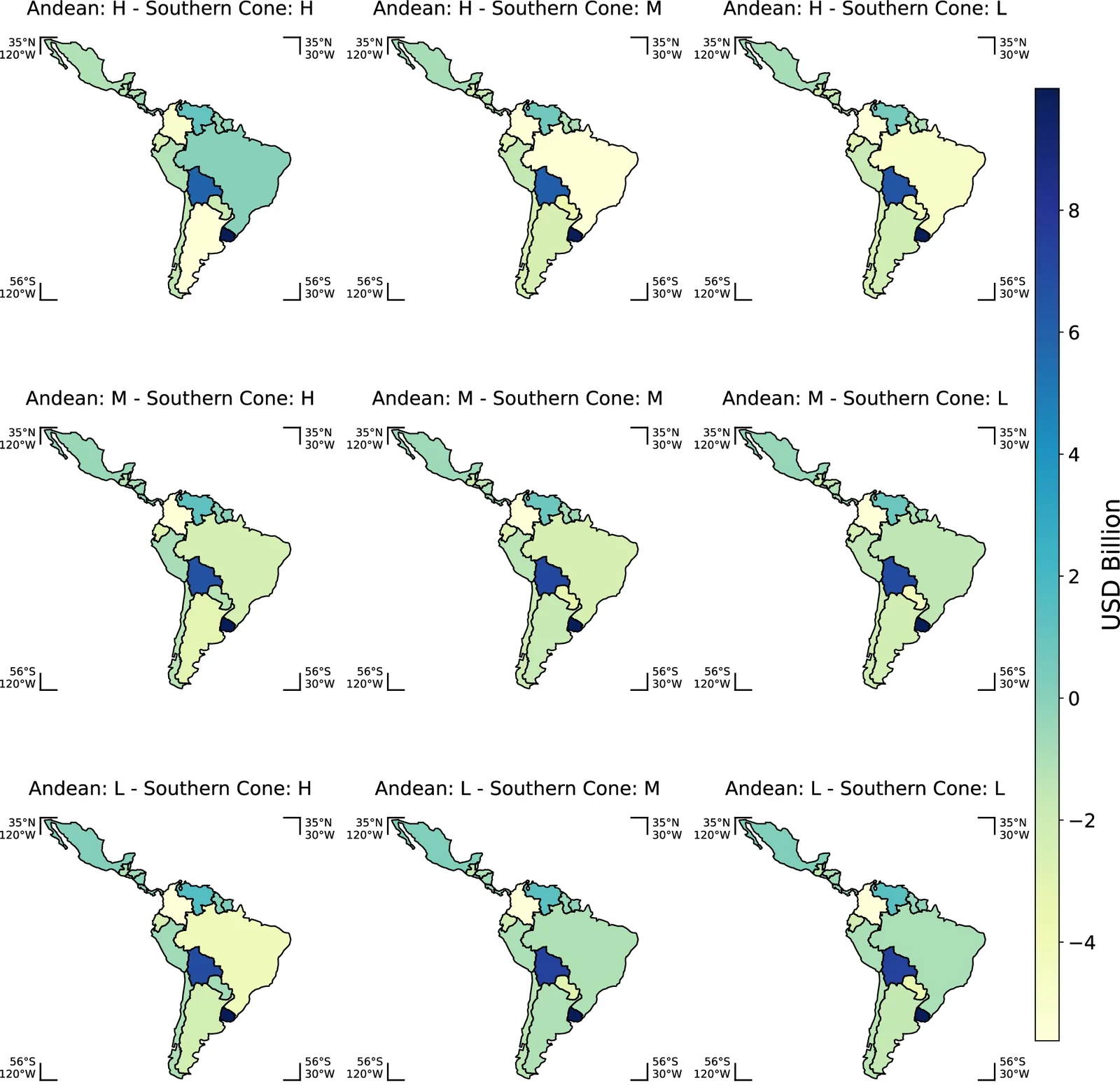
Economic loss from trusting. Quantifying risk aversion to regional integration. Heat maps show the potential `Economic Loss from Trusting' for individual Latin American countries in 2045 (in billions USD). This metric quantifies the economic risk exposure if a country relies heavily on an integrated regional system (forgoing national investments) that subsequently underperforms or fails to deliver expected benefits (scenario assumes no national sovereignty constraints; see Methods). Results are shown across a 3 × 3 matrix combining High (H), Medium (M), and Low (L) hydropower availability scenarios for the Andean (rows) and Southern Cone (columns) regions. Corner markings on each panel indicate the geographical extent of the maps, spanning 35^∘^N to 56^∘^S and 120^∘^W to 30^∘^W (WGS84, EPSG:4326; equirectangular projection). Color scale indicates loss magnitude (yellow = low/negative, blue = high). Bolivia and Uruguay consistently show the highest potential losses, reflecting greater perceived risk and potential aversion to deep integration, highlighting the need for trust-building and risk mitigation mechanisms.

The ‘Economic Loss from Trusting’ captures the economic risk perceived by countries relying on an integrated system that might underperform (Methods; Fig. 5). This quantifies risk aversion, identifying nations like Bolivia and Uruguay as particularly exposed (see Fig. 5 caption), thus highlighting the need for robust regional guarantees to foster trust. Evidence from other multinational grids shows that this risk exposure under uncertainty is itself a central determinant of whether countries commit to coordinated network investment rather than to national alternatives^49^.

**Fig. 6:**
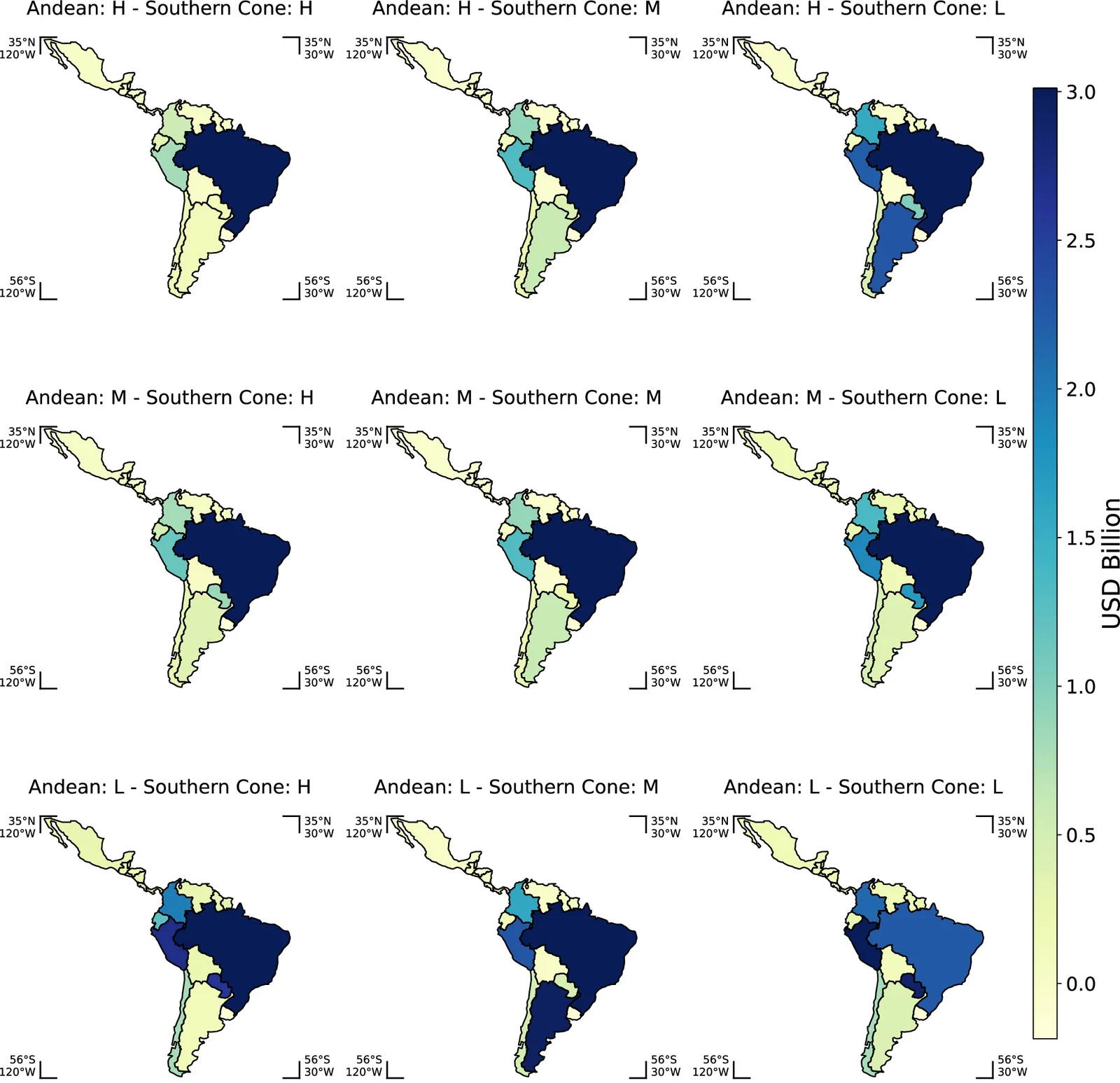
Economic loss from not trusting. The opportunity cost of fragmentation. Heat maps show the `Economic Loss from Not Trusting' for individual Latin American countries in 2045 (in billions USD). This metric quantifies the opportunity cost or economic damage incurred by not participating fully in an optimized regional integration (i.e., maintaining fragmentation, modeled here with sovereignty constraints active and potentially limited transmission compared to optimum). Results are shown across a 3 × 3 matrix combining High (H), Medium (M), and Low (L) hydropower availability scenarios for the Andean (rows) and Southern Cone (columns) regions. Corner markings on each panel indicate the geographical extent of the maps, spanning 35^∘^N to 56^∘^S and 120^∘^W to 30^∘^W (WGS84, EPSG:4326; equirectangular projection). Color scale indicates loss magnitude (yellow = low, blue = high). Losses are most severe under low hydro conditions. Brazil consistently incurs the largest absolute losses, followed significantly by Paraguay, Colombia, and Peru, indicating their strong economic incentive to pursue deeper regional cooperation.

The ‘Economic Loss from Not Trusting’ quantifies the substantial opportunity cost of continued fragmentation (Methods; Fig. 6). This reveals the direct economic penalty of the status quo, showing that Brazil, along with Paraguay, Colombia, and Peru, faces multi-billion dollar annual losses by failing to integrate deeply, especially under climate stress (see Fig. 6 caption).

### Leveraging quantified costs for political strategy

These quantified metrics serve as strategic political tools. The significant ‘Loss from Not Trusting’ pinpoints nations with the strongest economic incentive for integration, positioning Brazil as a potential policy entrepreneur and Paraguay, Colombia, and Peru as key allies^61^. Concurrently, ‘Loss from Trusting’ highlights where institutional design must actively mitigate perceived risks (e.g., for Bolivia and Uruguay) to enable broader participation. The analysis provides actionable intelligence for coalition building and targeted negotiation strategies based on quantified national interests and vulnerabilities.

While the politics stream remains the integration bottleneck, the climate crisis (problem stream) and viable solutions (policy stream) offer a window of opportunity. This study provides crucial leverage by demonstrating the quantifiable, multi-billion-dollar annual costs associated with political fragmentation, sovereignty constraints, and mistrust. This evidence, which quantifies the multi-billion-dollar costs of inaction and identifies specific national vulnerabilities, provides crucial leverage. Equipping negotiators and policy entrepreneurs with these concrete metrics, highlighting who bears the greatest losses from fragmentation and whose risks require mitigation, can help overcome historical inertia, foster necessary coalitions, and ultimately align the politics stream toward a secure, decarbonized regional energy future^62^.

## Discussion

This study offers a critical and timely contribution not only to the academic literature on energy policy and regional integration but, more pressingly, to the current geopolitical energy praxis in Latin America. This study moves beyond describing persistent political barriers by providing an actionable framework that combines the MSA with quantitative optimal energy expansion modeling to translate abstract political dynamics, such as historical distrust and sovereignty concerns, into concrete, billion-dollar economic trade-offs. This assessment of the political costs of inaction, developed in the politics-stream analysis, provides policymakers and policy entrepreneurs with actionable, evidence-based leverage. The analysis indicates that the escalating climate crisis, with its severe impacts on regional energy security, is not merely a “focusing event”, but a catalyst that can help overcome decades of stagnation in regional integration policies^2,12,62^.

The convergence of streams is palpable. It includes a highly salient Problem Stream, as examined in the problem-stream subsection, where climate-induced energy disruptions are no longer abstract forecasts but immediate crises; a well-evidenced Policy Stream, as developed in the policy-stream subsection, where regional integration is demonstrably a technically viable and economically superior solution; and an evolving Politics Stream, as analyzed in the politics-stream subsection, evidenced by emerging high-level commitments like the 2023 Brasília Consensus and invigorated multilateral funding^69,70^. Further evidenced by recent calls to action and strategic analyses from key regional institutions like OLACDE, which highlight both the urgency and the renewed potential for advancing electrical integration^108^. This stream convergence indicates that Latin America may now be transiting more than just an agenda-setting window; the region appears to have entered a decision window, where long-standing proposals for regional electricity integration can be translated into concrete policy action ^119^.

Nevertheless, this optimistic reading must be tempered by a recognition of the broader geopolitical economy in which Latin American integration is embedded. The region’s energy transition is not occurring in a vacuum. The Global North and China are actively competing for preferential access not only to critical minerals, including lithium, copper, nickel, graphite, and rare earth elements, but also to a broader range of commodities and associated value chains that underpin renewable energy infrastructure, storage, transmission, and electrification technologies^109,110,112,113^. The pace and shape of Latin American integration, therefore, depend not solely on intra-regional political will, but also on the decisions and strategic interests of these external actors, who shape financing terms, trade rules, and technology-transfer conditions. These dynamics introduce structural tensions that the MSA framework, focused primarily on policy-stream convergence and political coupling, does not fully capture.

Drawing directly on Harvey’s concept of accumulation by dispossession, these pressures can be interpreted as part of a broader risk that the financialization of green infrastructure, the privatization of strategic resources, and the external control of critical mineral value chains may transfer a significant share of Latin America’s renewable and mineral rents to external actors rather than to regional populations^120^. Recent debates on green colonialism and lithium extraction in Latin America further show that poorly governed energy-transition projects can reproduce territorial inequalities, socio-environmental conflicts, and forms of ecological dispossession^121,122^. Regional power integration, if governed through strong multilateral institutions with explicit benefit-sharing, socio-environmental safeguards, and regionally owned planning mechanisms, can serve as a counterweight to these dynamics, ensuring that the region’s renewable wealth becomes a collective asset rather than a site for external appropriation. This consideration further reinforces the urgency of building robust, regionally owned governance frameworks as a prerequisite for integration that is both effective and equitable.

Hence, the core contribution of this paper is to alert readers to the openness of this crucial but fading decision window, as well as support those strategic actors by providing an evidence-based foundation, through quantified geopolitical and economic costs of non-cooperation, for seizing it and moving integration from political discourse into institutionalized policy.

To seize this critical juncture, this study proposes a coordinated regional energy policy, headlined by a foundational “Latin American Energy Pact”. This high-level political agreement should aim for the phased establishment of a LA Electricity Integration System (SIEAL). A critical deliverable of this Pact should be the development of a coordinated legal corpus, akin to a dedicated LA Energy Treaty, an instrument that, to date, does not exist at the regional level, which is precisely what motivates its proposal here. The notion of a regional energy treaty aimed at promoting collaboration and a stable investment environment, especially in South America, has been previously examined^73^, highlighting its capacity to alleviate risks such as resource nationalism and draw essential infrastructure investment. The analysis acknowledges, however, that many analysts remain skeptical about the political and geopolitical feasibility of such an ambitious instrument, given the persistent sovereignty concerns, ideological divergences among governments, and the historically slow pace of institutionalization in Latin American regionalism^74,109,111^. Rather than dismissing these obstacles, this study treats them as precisely the barriers that the quantified costs of inaction presented in this study are designed to help overcome. This view can be extended to Latin America through a macro-treaty that would aim not to reinvent existing frameworks but to harmonize and ensure compatibility among current bilateral and sub-regional agreements (such as SIEPAC, Itaipú, Yacyretá, and Salto Grande) and broader initiatives (e.g., past Union of South American Nations (UNASUR) energy efforts and Mercosur energy forums), thereby building on existing political capital rather than starting from scratch. In this sense, key actions must include (a) standardizing core technical and commercial rules for cross-border electricity trade to reduce regulatory costs and administrative barriers; (b) prioritizing strategic interconnection corridors with high energy complementarity and mobilizing joint public-private resources for transmission and storage; (c) creating a Regional Energy Integration Fund (e.g., via Development Bank of Latin America (CAF)/Inter-American Development Bank (IDB)) to de-risk investments and ensure equitable benefit distribution; (d) implementing a phased regional rollout with clear milestones to build mutual trust and demonstrate tangible benefits; and (e) establishing simple monitoring indicators (e.g., trade volumes, emission reductions, cost savings) for accountability and adaptive management. Existing institutions like OLACDE/CIER should be empowered to support SIEAL’s implementation and ongoing coordination.

Further crucial steps include directly addressing the ‘cost of trust’ through regional risk-sharing mechanisms and harmonized contracts to reduce investment uncertainty, particularly for risk-averse nations^49^ (the subsection ‘Quantified Costs of Political Factors’). These mechanisms must be institutionally embedded rather than ad hoc, designed to persist across electoral cycles and changes in government orientation. Enhanced planning and coordination via a Regional Master Plan for Interconnections, supported by multilateral platforms involving all key stakeholders, is also essential; such a Master Plan must incorporate transparent benefit-distribution arrangements that make the gains from integration visible and credible to all participating nations, including those currently most skeptical. Finally, activating and empowering diverse policy entrepreneurs across governments, multilateral development banks (MDBs), academia, and civil society is vital to champion integration and build the broad coalitions needed to overcome institutional inertia.

A concrete continuation of the present paper should therefore be the design of a Latin American Transmission Allocation Mechanism. This mechanism is beyond the scope of the current analysis, but follows directly from its findings. If quantified benefits and trust costs are to inform real negotiations, future work must specify how transmission expansion costs, congestion rents, risk-sharing obligations, benefit-distribution rules, and compensation for adverse cross-border impacts would be allocated among participating countries. Developing such a mechanism would translate the political-cost evidence provided here into an implementable institutional instrument for bilateral projects governed by multilateral rules.

While this study’s MSA framework illuminates the current opportunity and the modeling quantifies key trade-offs, the study acknowledges limitations. The MSA does not fully model implementation intricacies or power asymmetries, and the energy simulations assume idealized cooperation^84^. Future research could incorporate complementary theories (e.g., Punctuated Equilibrium^123^, Advocacy Coalition Framework^124^) and simulate asymmetric cooperation^125^ to deepen understanding of energy policy change in Latin America and other fragmented regions.

Latin America confronts a pivotal, time-sensitive opportunity to transform its fragmented power systems into a resilient, integrated regional network. This study has demonstrated, through the combined lens of the MSA and quantitative power system modeling, that the three necessary conditions for major policy change are currently aligned. These conditions include a highly salient problem stream driven by climate-induced energy disruptions; a well-evidenced policy stream where regional integration is technically viable and economically superior; and an evolving politics stream signaled by emerging multilateral commitments and renewed institutional momentum. Furthermore, the current global geopolitical juncture, marked by conflicts in Ukraine and the Middle East, and the resulting volatility in fossil fuel availability and costs, acts as a powerful exogenous trigger. This macro-level instability only reinforces the urgency for Latin America to insulate its energy security through deep regional integration, thereby accelerating the opening of this policy window.

Quantitatively, the simulations show that continued fragmentation increases total system costs by up to 7%, reduces resilience to hydroclimatic shocks, and concentrates benefits within narrow cooperating blocs. By contrast, full regional integration can reduce annual CO_2_ emissions by up to 225 MtCO_2_ by 2045 and unlock economic savings of up to 32 billion USD yr^−^^1^, particularly under conditions of water scarcity. The geopolitical cost of prioritizing national sovereignty over regional synergy ranges from 4.6–7.9 billion USD annually by 2045, providing concrete, evidence-based leverage for negotiators and policy entrepreneurs.

However, translating this open policy window into effective and lasting action requires more than technical and economic arguments. Greater political cohesion is a necessary condition for the climate emergency to produce real institutional change, but it will not be sufficient on its own. Overcoming entrenched differences in national interests, historical distrust, and geopolitical tensions demands the deliberate construction of durable cooperative mechanisms, including regional risk-sharing instruments, harmonized regulatory frameworks, and transparent benefit-distribution arrangements, that make integration credible and self-sustaining over time. Without these institutional foundations, even the most compelling cost arguments risk remaining rhetorical.

The time to act is now, before this critical window closes. Bold political leadership, a shared strategic vision, and the collective will to build lasting cooperative institutions represent the final, indispensable ingredients for securing Latin America’s integrated, decarbonized energy future.

## Methods

This study employs a mixed-methods research design, combining qualitative political analysis grounded in the MSA with quantitative, long-term energy system expansion modeling. This integrated framework allows for a comprehensive assessment of the political-economic challenges and techno-economic opportunities for enhanced regional electricity integration in Latin America, examining both the structural drivers of historical policy inertia and the concrete benefits of increased cooperation.

### Qualitative application of MSA

The MSA, as conceptualized by Kingdon^61^, provides the theoretical lens to understand how major policy change becomes possible when its three constituent streams (problem, policy, and politics) converge, often facilitated by ‘focusing events’ and ‘policy entrepreneurs’. The conceptualization is made in three stages. In the initial stage, the study conducts a comprehensive review of three bodies of source material. The first is the academic literature, covering the historical political economy of Latin American regionalism and sovereignty^24,25,73–77,126^; the trajectory of binational hydropower projects and the policy-process studies that have examined them^79–81,83–86^; the experience of regional electricity integration and market coupling^29,30,32–35^; the institutional and governance barriers identified in Latin America and in comparable regions^36–38,51,52^; and the literature on energy transitions and policy frameworks^54–57,62^. This review also covers the observed and projected impacts of climate variability on hydropower and energy security^9,13–18^; the modelling of climate change effects on regional hydrological and renewable resources^88,89,91–96^; the interaction between climate stress, electricity markets, and long-term energy scenarios^97,98,103,105^; and power system integration and decarbonization pathways modelled for the region^10,20–23,99–101^. The second body comprises energy policy reports from key regional and international organizations, addressing both the climate vulnerability of energy systems^1,11,12,106^ and the status of and barriers to regional electricity integration^26–28,82,127^, together with recent multilateral financing commitments^70,71^. The third consists of recent, pivotal policy documents and political declarations^69^. Together, these sources comprise more than 100 works spanning 70 years of history and are reviewed to understand the historical context and identify long-term patterns (informing the subsection ‘MSA patterns in the history of Latin American integration’). In the second stage, to assess the current policy window (informing the Discussion section), the study uses a coding process^128^ for interpreting these secondary data (reports, declarations, academic literature) that involves identifying text excerpts and documented events aligning with predefined codes based on MSA theory, including ‘problem stream indicators’, ‘policy stream elements’ (including proposed solutions and their perceived viability), ‘politics stream signals’ (national mood, political forces, government changes), ‘political entrepreneurs’, ‘policy entrepreneurs’, ‘focusing events’, ‘policy windows’, ‘agenda window’, and ‘decision window’. The analytical period for the contemporary analysis is primarily chosen to correspond with the availability of detailed data and recent policy shifts (2020–2025). The final step involves interpreting these coded elements and documented events, using deductive inference to reconstruct the interplay of the streams and assess the nature and openness of the historical patterns of MSA in LA energy integration and the current policy window. A timeline approach is used internally to organize facts and events within the problem, policy, and political streams to identify their convergence. Following this, these facts are framed by a quantitative analysis of the impacts of LA integration on regional power system evolution.

### Quantitative regional power system expansion model

To quantify the technical, economic, and CO_2_ emission implications of different regional integration pathways and specific political-institutional constraints, this study develops and uses a detailed multi-country power system expansion model for Latin America.

To enhance transparency and facilitate reproducibility, the complete set of simulation outputs (generation mixes, transmission flows, investments, and costs) is accessible through an interactive visualization platform at https://explorer.energia.la/.

### Model formulation and objectives

The model is a multi-period linear optimization tool, formulated to co-optimize investment in and dispatch of generation, storage, and cross-border transmission infrastructure across 21 Latin American nations. The optimization horizon spans from 2025 to 2050, with results typically analyzed in five-year increments. The objective function is to minimize the total discounted system costs, which include annualized capital expenditures for additional generation, storage, and transmission assets, as well as fixed and variable operational costs where relevant. The variable OPEX for thermal technologies are reported in Supplementary Table 2, while the annualized CAPEX per unit of installed capacity for each technology are presented in Supplementary Table 3. The model is implemented in Python, leveraging the Pyomo optimization modeling language, and is solved using Gurobi. The model operates on a multi-decadal time horizon, covering the period 2025 to 2050 in five-year increments. To capture daily variability in renewable generation and demand, it uses representative days with hourly resolution, allowing the simulation of intraday dynamics and the evaluation of flexibility needs. Key decision variables include (a) generation capacity additions, by technology and country; (b) energy dispatch across hours and seasons; (c) storage deployment and operation, particularly batteries and hydro reservoirs; and (d) expansion of cross-border transmission capacity, including both existing and planned interconnections (see Supplementary Methods). The model includes several critical technical and policy constraints, ensuring realistic operation. These constraints include reliability requirements, including minimum reserve margins at national and regional levels; emissions limits, allowing comparison of high- and low-carbon scenarios; minimum renewable penetration targets, consistent with national energy plans and climate commitments; and transmission modeling, incorporating power losses, capacity limits, and inter-country flow constraints based on existing and planned infrastructure. Further details can be seen in Supplementary Table 5. Spatially, the model represents each country as a node in a regional network, with transmission links reflecting physical interconnections. It captures both domestic generation mix and import/export dynamics, enabling the evaluation of regional synergies and the role of integration in enhancing resilience.

### Geographical scope and model inputs

The geographical scope encompasses all the countries in South America, Central America, and Mexico. This selection covers the vast majority of the region’s electricity demand and renewable resource potential.

Key input data and variables are derived from official national energy plans, IDB, OLADE and IEA databases^19,106,108,129^, recent technical reports, and relevant literature. National-level hourly demand profiles in megawatts (MW) projected to 2050 are used to characterize electricity demand, accounting for baseline gross domestic product (GDP) growth, population trends, and varying electrification pathways (e.g., for transport and industry). Technology cost and performance parameters include investment costs (USD kW^−^^1^), fixed operation and maintenance (O&M) costs (USD kW^-1^ yr^−1^), variable O&M costs (USD MWh^−1^), economic lifetimes, efficiencies, and key operational parameters (e.g., ramp rates, availability factors) for a portfolio of generation technologies (solar photovoltaic (PV), onshore/offshore wind, reservoir and run-of-river hydropower, natural gas-fired combined-cycle gas turbines (CCGTs), open-cycle gas turbines (OCGTs), geothermal, and biomass), battery energy storage systems (BESS), and high-voltage direct current (HVDC)/high-voltage alternating current (HVAC) transmission lines. Renewable energy potentials are represented through spatially explicit hourly generation profiles (per unit of capacity) for solar irradiance and wind speeds across different resource zones within countries, together with country-specific developable potentials in gigawatts (GW) or terawatt-hours annually (TWh yr^−1^) for hydro, solar, and wind, adjusted for geographical, technical, and broad socio-environmental considerations. Emission factors and fuel costs comprise technology-specific CO_2_ emission factors (tonnes of CO_2_ per MWh, tCO_2_ MWh^−1^) and projected international/regional costs for fossil fuels (e.g., natural gas). Finally, the model is initialized with the existing generation fleet (capacity, technology, age), storage capacities, and current cross-border interconnection infrastructure (voltage levels, thermal capacity limits), with firmly committed expansion projects reported in national plans also incorporated.

National electricity systems are represented as single nodes, and transmission constraints between countries are explicitly modeled as capacitated links, respecting regulatory and geopolitical boundaries for energy exchange.

These assumptions allow for a clear estimation of the structural economic and environmental costs associated with different degrees of political fragmentation and the corresponding benefits of enhanced cooperation under specified, idealized conditions.

### Key model constraints

The optimization is subject to several critical technical and policy constraints to ensure realistic system operation. An energy balance constraint requires that hourly electricity generation, including net imports, meet demand at each national node for all representative periods. Reliability requirements are enforced through minimum planning reserve margins (or other reliability metrics, such as the loss of load probability, where used) at national or regional levels, depending on the scenario, to ensure security of supply. Renewable energy targets and emission limits are incorporated through national or regional renewable energy penetration goals and CO_2_ emission reduction targets (or carbon prices) consistent with stated climate commitments or policy ambitions. Generation and storage operational limits are imposed through constraints on ramping capabilities, minimum stable generation levels, energy storage operational characteristics (efficiency, charge/discharge rates, storage capacity), and hydro reservoir energy limits and flow constraints. Transmission constraints further restrict power flow on existing and candidate interconnection lines according to their thermal capacities, with electrical losses accounted for accordingly.

### Scenario design for policy analysis

To evaluate the impact of regional integration under varying political, institutional, and physical conditions, this study develops a comprehensive set of policy-relevant scenarios. This design enables sensitivity analysis of key system outcomes (e.g., total costs, CO_2_ emissions, energy security metrics) under different degrees of inter-country cooperation and specific exogenous stressors. The scenario architecture is structured around three key dimensions: the level of regional integration, the institutional assumptions used to operationalize political factors, and the climate and demand stressors applied across scenarios.

The level of regional integration is the primary dimension explored, spanning three pathways. Full Integration assumes complete coordination in planning, investment, and dispatch across all 21 modeled countries, with minimal institutional barriers to electricity trade. Partial Integration simulates cooperation among specific sub-regional blocs (e.g., Andean Community, Southern Cone, SIEPAC), reflecting existing political alliances, historical precedents, or plausible intermediate steps toward wider integration. Fragmented Systems (or Status Quo) describe cases where each country optimizes its system primarily on a national basis, with limited or no additional cross-border coordination beyond currently existing interconnections and bilateral agreements.

To explore the impact of political realities, specific institutional parameters are varied to operationalize political factors. Sovereignty preference, captured through the Geopolitical Cost, is modeled by comparing the fully integrated regional optimum with scenarios where national systems are constrained to prioritize a certain degree of energy self-sufficiency or limit reliance on specific neighbors, even if this results in higher regional costs; the economic penalty incurred represents the ‘Geopolitical Cost’, and the formulation and further explanation of this cost can be found in Supplementary Note 1. Trust level costs, encompassing both the Cost of Trusting and the Cost of Not Trusting, are reflected through adjustments to inter-country electricity exchange limits, differentiated risk premiums applied to investments in cross-border infrastructure, or varying transaction costs on traded electricity, particularly in partial integration or fragmented scenarios; lower perceived trust translates into stricter operational constraints or higher financial hurdles for cooperative projects, and the formulation and further explanation of these costs can also be found in Supplementary Note 1.

All integration levels and institutional settings are further analyzed under a range of climate and demand stressors, including varying hydrological conditions for hydropower (e.g., average inflow years, multi-year dry periods based on historical El Niño cycles) and different demand growth trajectories (e.g., baseline projections vs. high electrification scenarios driven by accelerated uptake of electric vehicles and industrial decarbonization). The geographic illustration of the achieved optimal interconnections can be found in Supplementary Note 4.

This multi-dimensional scenario matrix allows for the robust quantification of metrics such as the ‘Geopolitical Cost’, the ‘Economic Loss from Trusting’, and the ‘Economic Loss from Not Trusting’, as presented and discussed in the subsections ‘Quantified Costs of Political Factors’ and ‘Leveraging Quantified Costs for Political Strategy’. It is important to note that these scenarios are designed as analytical tools to illuminate strategic trade-offs and guide policy design, rather than to provide precise predictions of future outcomes.

### Model validation

To ensure the credibility of the model outputs, the base-year (2025) configuration and generation mix are calibrated against recent historical data (e.g., 2022–2023) from national energy balances and OLADE statistics. Likewise, the results are compared with relevant papers in the literature in order to ensure that the magnitude order is coherent^21,23,68,105^. Key model parameters, such as technology costs and renewable potentials, are benchmarked against IEA reports, International Renewable Energy Agency (IRENA) data, and recent regional studies ^12,106,108,129^. While perfect replication of complex national systems is challenging, these steps ensure that the model provides a plausible representation of regional energy dynamics and a robust basis for comparative scenario analysis.

### Reporting summary

Further information on research design is available in the Nature Portfolio Reporting Summary linked to this article.

## Supplementary information

Supplementary Information

Reporting Summary

Transparent Peer Review file

## Source data

Source Data

## Data Availability

The datasets used to construct the energy system model are derived from publicly available sources, including IDB, OLADE, and IEA reports^12,19,106^. Additional regional data on transmission infrastructure, renewable potential, and technology costs were obtained from official reports and academic literature^21,23,88,105^. Model input data and processed model output data, which can be used to fully reproduce all analyses presented in this research, have been deposited in Zenodo^130^. In addition, the complete set of simulation outputs (generation mixes, transmission flows, investments, and costs) is publicly accessible through an interactive visualization platform at https://explorer.energia.la/, whose source code is openly available at https://github.com/gerardoblancopy/LatamEnergyIntregrationExplorer. Source data are provided with this paper.

## Code availability

The energy system optimization model is implemented in Python using Pyomo and solved with Gurobi. The model code, scenario generation scripts, post-processing routines, visualization scripts, and input files required to reproduce the main results and figures are publicly available in a permanent Zenodo repository at https://doi.org/10.5281/zenodo.19775192.

## References

[1] C3S, C. C. C. S. Global climate highlights 2024. https://climate.copernicus.eu/global-climate-highlights-2024 (2025).

[2] Yalew, S. G. et al. Impacts of climate change on energy systems in global and regional scenarios. *Nat. Energy* **5**, 794–802 10.1038/s41560-020-0664-z (2020).

[3] Webb, D., Hanssen, O. N. & Marten, R. The health sector and fiscal policies of fossil fuels: an essential alignment for the health and climate change agenda. *BMJ Glob. Health* **8**, e012938 (2023). 10.1136/bmjgh-2023-012938 PMC10565167 37813437

[4] Speizer, S. et al. Integrated assessment modeling of a zero-emissions global transportation sector. *Nat. Commun.* **15**, 4439 (2024). 10.1038/s41467-024-48424-9 PMC11126718 38789428

[5] Rickman, J. et al. The challenge of phasing-out fossil fuel finance in the banking sector. *Nat. Commun.* **15**, 7881 (2024). 10.1038/s41467-024-51662-6 PMC11387497 39256349

[6] Papadis, E. & Tsatsaronis, G. Challenges in the decarbonization of the energy sector. *Energy* **205**, 118025 (2020).

[7] Peng, W., Yang, J., Lu, X. & Mauzerall, D. L. Potential co-benefits of electrification for air quality, health, and Co_2_ mitigation in 2030 China. *Appl. Energy* **218**, 511–519 (2018).

[8] Sheinbaum, C., Ruíz, B. J. & Ozawa, L. Energy consumption and related CO_2_ emissions in five Latin American countries: changes from 1990 to 2006 and perspectives. *Energy* **36**, 3629–3638 10.1016/j.energy.2010.07.023 (2011).

[9] Gonzalez-Salazar, M. & Poganietz, W. Evaluating the complementarity of solar, wind and hydropower to mitigate the impact of El Niño Southern Oscillation in Latin America. *Renew. Energy* **174**, 453–467 (2021).

[10] Marinkovic, C. & Vogt-Schilb, A. Is energy planning consistent with climate goals? assessing future emissions from power plants in Latin America and the Caribbean. *Nat. Sustain.* **6**, 577–587 (2023).

[11] International Energy Agency (IEA). Climate impacts on latin american hydropower. Tech. Rep., Organisation for Economic Co-operation and Development (OECD) (2023).

[12] Organización Latinoamericana de Energía (OLADE), Tecnalia & Agencia Española de Cooperación Internacional para el Desarrollo (AECID). Vulnerabilidad y riesgo de los sistemas energéticos de América Latina y el Caribe ante las amenazas del cambio climático: informe final del proyecto SCREEN ALC. Tech. Rep. OLADE, Quito, Ecuador (2023).

[13] Henao, F., Rodriguez, Y., Viteri, J. P. & Dyner, I. Optimising the insertion of renewables in the Colombian power sector. *Renew. Energy* **132**, 81–92 https://www.sciencedirect.com/science/article/pii/S0960148118308966 (2019).

[14] Besnier, J., Getirana, A., Beaudoing, H. & Lakshmi, V. Characterizing the 2019-2021 drought in La Plata river basin with GLDAS and SMAP. *J. Hydrol.: Reg. Stud*. Accessed: 2025-05-13 (2024).

[15] Naranjo-Silva, S. et al. Hydropower scenarios in the face of climate change in Ecuador. *Sustainability* **15**, 10160 (2023).

[16] Lala, J. O., Carvajal Mora, H. R., Orozco Garzón, N. V., Vega, J. & Ohishi, T. Examining the evolution of energy storing in the ecuadorian electricity system: a case study. *Energies* **17**, 3500 (2024).

[17] Weng, W., Becker, S. L., Lüdeke, M. & Lakes, T. Landscape matters: insights from the impact of mega-droughts on Colombia’s energy transition. *Environ. Innov. Societal Transit.* **36**, 1–16 (2020).

[18] Viviescas, C. et al. Contribution of variable renewable energy to increase energy security in Latin America: Complementarity and climate change impacts on wind and solar resources. *Renew. Sustain. Energy Rev.* **113**, 109232 (2019).

[19] Paredes, J. R. La red del futuro: desarrollo de una red eléctrica limpia y sostenible para América Latina. 10.18235/0000937 (2017).

[20] Bataille, C. et al. Net-zero deep decarbonization pathways in Latin America: Challenges and opportunities. *Energy Strategy Rev.* **30**, 100510 10.1016/j.esr.2020.100510 (2020).

[21] Santos da Silva, S. R. et al. Power sector investment implications of climate impacts on renewable resources in Latin America and the Caribbean. *Nat. Commun.* **12**, 1276 (2021). 10.1038/s41467-021-21502-y PMC7904843 33627651

[22] Moreno, J., Medina, J. P. & Palma-Behnke, R. Latin America’s renewable energy impact: Climate change and global economic consequences. *Energies* **17**, 179 (2024).

[23] Kober, T. et al. A multi-model study of energy supply investments in Latin America under climate control policy. *Energy Econ.* **56**, 543–551 (2016).

[24] Rippy, J. F. British investments in Latin-American electrical utilities. * Hispanic Am. Historical Rev.* **34**, 251–255 (1954).

[25] Ewald, E. & Angland, D. Regional integration of electric power systems. *IEEE Spectr.* **1**, 96–101 (1964).

[26] Comisión de Integración Energética Regional (CIER). Historia. https://www.cier.org/es-uy/Paginas/Historia.aspx. Consultado el 11 de noviembre de 2024 (1964).

[27] Comisión de Integración Energética Regional (CIER) & Corporación Andina de Fomento (CAF). Nuevas oportunidades de interconexión eléctrica en América Latina. Segunda edición. Tech. Rep., CIER y CAF, Bogotá, Colombia. Disponible en: publicaciones.caf.com y www.cier.org.uy (2012).

[28] Ruchansky, B. Integración eléctrica en América Latina: antecedentes, realidades y caminos por recorrer. Estudios y Perspectivas 113, Comisión Económica para América Latina y el Caribe (CEPAL) and Deutsche Gesellschaft für Internationale Zusammenarbeit (GIZ), México, D.F. URL https://core.ac.uk/download/38672350.pdf. Document number: LC/L.3623; LC/MEX/L.1085 (2013).

[29] Raineri, R., Dyner, I., Franco, C. & Castañeda, J. C. Latin America energy integration: A retrospective and future outlook. *Energy Policy* **52**, 1–10 10.1016/j.enpol.2012.08.054 (2013).

[30] Ochoa, C. & Ackere, A. Winners and losers of market coupling. *Energy* **80**, 522–534 (2015).

[31] Batlle, C., Barroso, L. & Pérez-Arriaga, I. The changing role of the state in the expansion of electricity supply in Latin America. *Energy Policy* **38**, 7152–7160 (2010).

[32] Gómez-Ramírez, G., García-Santander, L., Rojas-Morales, J. R., Lazkano-Zubiaga, M. & Meza, C. Electrochemical storage and flexibility in transfer capacities: Strategies and uses for vulnerable power grids. *Energies* **17**, 5878 (2024).

[33] Larsen, M., Jenss, A., Schuetze, B. & Cupers, K. Elsewheres of the unbuilt: The global effects of transnational energy infrastructure projects. *Int. J. Urban Regional Res.* **48**, 916–935 (2024).

[34] Suffian, S., Ponce de Leon Barido, D., Kabalan, M. & Singh, P. Grid adequacy and cooperation in Central America: A data-driven analysis. *2017 IEEE Power & Energy Society General Meeting* 1–5 (2017).

[35] Suffian, S. & Singh, P. Assessing the added value of coordinated economic dispatch in Central America. *2018 IEEE 38th Central America and Panama Convention (CONCAPAN XXXVIII) 1–6* (2018).

[36] Redondo-Olivar, D., Olmos, L. & Pérez-Arriaga, I. J. Modeling for the regional integration of electricity markets: Application to the Andean countries. *Energy Econ.* **74**, 672–685 10.1016/j.eneco.2018.07.025 (2018).

[37] Santos-Carrillo, F. & Vassaux, L. A. P. Domestic regimes and national preferences as factors of regionalism’s crisis: The case of Guatemala’s regional integration policy. *Revista Brasileira de Política Internacional*. 10.1590/0034-7329202300215 (2023).

[38] Huda, M. & McDonald, M. Regional cooperation on energy in South Asia: Unraveling the political challenges in implementing transnational pipelines and electricity grids. *Energy Policy* **98**, 73–83 (2016).

[39] Tesfamichael, M. & Fuchs, J. Navigating complexity: integrating political realities into energy system modelling for effective policy in sub-Saharan Africa. *Prog. Energy* **6**, 043001 (2024).

[40] Ogeya, M. & Lambe, F. The political economy of mini-grid electricity development and innovation in Kenya. *Renew. Sustain. Energy Transit.* **6**, 100092 (2025).

[41] Roumkos, C., Biskas, P. & Marneris, I. Integration of European electricity balancing markets. *Energies* **15**, 2240 (2022).

[42] Wu, Y. Electricity market integration: Global trends and implications for the EAS region. *Energy Strategy Rev.* **2**, 138–145 10.1016/J.ESR.2012.12.002 (2013).

[43] Pineau, P.-O. Electricity sector integration in West Africa. *Energy Policy* **36**, 210–223 10.1016/J.ENPOL.2007.09.002 (2008).

[44] Puka, L. & Szulecki, K. The politics and economics of cross-border electricity infrastructure: a framework for analysis. *Energy Res. Soc. Sci.* **4**, 124–134 (2014).

[45] Brinkerink, M., Ó Gallachóir, B. & Deane, P. A comprehensive review on the benefits and challenges of global power grids and intercontinental interconnectors. *Renew. Sustain. Energy Rev.* **107**, 274–287 (2019).

[46] Makrygiorgou, D. I., Andriopoulos, N., Georgantas, I., Dikaiakos, C. & Papaioannou, G. Cross-border electricity trading in Southeast Europe towards an internal European market. *Energies* **13**, 6653 (2020).

[47] Loureiro, M., Claro, J. & Fischbeck, P. Coordinating cross-border electricity interconnection investments and trade in market-coupled regions. *Int. J. Electr. Power Energy Syst.* **109**, 296–305 10.1016/J.IJEPES.2018.07.003 (2019).

[48] Biancardi, A., Mendes, C. & Staffell, I. Battery electricity storage as both a complement and substitute for cross-border interconnection. *Energy Policy* **189**, 114134 (2024).

[49] Konstantelos, I., Moreno, R. & Strbac, G. Coordination and uncertainty in strategic network investment: case on the North Seas Grid. *Energy Econ.* **64**, 131–148 10.1016/j.eneco.2017.03.022 (2017).

[50] Dutt, D. Weak states, fast transitions? Exploring the role of actors, governance capacity, and tensions in indian energy politics. *Energy Res. Soc. Sci.* **94**, 102876 (2022).

[51] Valdivia, G. Oil frictions and the subterranean geopolitics of energy regionalisms. *Environ. Plan. A: Econ. Space* **47**, 1422–1439 10.1177/0308518X15595764 (2015).

[52] Iranzo, Á & Caballero, S. The periphery at the centre: an analysis of Latin American regionalism from the borders. *Space Polity* **24**, 346 – 361 (2020).

[53] Vaidya, R., Yadav, N., Rai, N., Neupane, S. & Mukherji, A. Electricity trade and cooperation in the bbin region: lessons from global experience. *Int. J. Water Resour. Dev.* **37**, 439–465 10.1080/07900627.2019.1566056 (2019).

[54] Cherp, A., Vinichenko, V., Jewell, J., Brutschin, E. & Sovacool, B. Integrating techno-economic, socio-technical and political perspectives on national energy transitions: a meta-theoretical framework. *Energy Res. Soc. Sci.* **37**, 175–190 (2018).

[55] Urban, F. *Low Carbon Transitions for Developing Countries* (Routledge, Abingdon, UK, 2014). URL https://www.routledge.com/Low-Carbon-Transitions-for-Developing-Countries/Urban/p/book/9780415826458.

[56] Alvarez, J. M., Fernandez-Vazquez, S., Macedo, S. V. & Ortega-Andrade, A. Enhancing energy security and efficiency through a regional integration policy: the case of the Southern Cone. *Energy Policy* **195**, 114384 (2024).

[57] Hochstetler, K. *Political Economies of Energy Transition: Wind and Solar Power in Brazil and South Africa* (Cambridge University Press, Cambridge, UK). 10.1017/9781108898376 (2020).

[58] Rubanda, M. E., Senyonga, L., Ngoma, M. & Adaramola, M. S. Electricity trading in energy market integration: a theoretical review. *Energies* **16**, 103 (2023).

[59] Lenhart, S. & Fox, D. Participatory democracy in dynamic contexts: a review of regional transmission organization governance in the United States. *Energy Res. Soc. Sci.* **83**, 102345 (2022).

[60] Johnathon, C., Agalgaonkar, A., Kennedy, J. & Planiden, C. Analyzing electricity markets with increasing penetration of large-scale renewable power generation. *Energies* **14**, 7618 (2021).

[61] Kingdon, J. W. *Agendas, Alternatives, and Public Policies* 2nd edn. Longman Classics in Political Science (Longman Pub Group, New York, 2011).

[62] Cavalcante, L. et al. From creeping crisis to policy change: the adoption of drought preparedness policy in Brazil. *Water Policy* **25**, 949–965 (2023).

[63] Amri, M. & Logan, D. Policy responses to COVID-19 present a window of opportunity for a paradigm shift in global health policy: an application of the multiple streams framework as a heuristic. *Glob. Public Health* **16**, 1187–1197 10.1080/17441692.2021.1925942 (2021). 34044747

[64] Tanaka, Y., Chapman, A. J., Tezuka, T. & Sakurai, S. Putting the process into the policy mix: simulating policy design for energy and electricity transitions in Japan. *Energy Res. Soc. Sci.* **70**, 101702 10.1016/j.erss.2020.101702 (2020).

[65] Shephard, D. D. et al. Kingdon’s multiple streams approach in new political contexts: consolidation, configuration, and new findings. *Governance* **34**, 523–543 (2021).

[66] Eckersley, P. & Lakoma, K. Straddling multiple streams: focusing events, policy entrepreneurs and problem brokers in the governance of English fire and rescue services. *Policy Stud.* **43**, 1001–1020 10.1080/01442872.2021.1892620 (2021).

[67] Pereira, C., Singh, S. P. & Mueller, B. Political institutions, policymaking, and policy stability in Latin America. *Lat. Am. Politics Soc.* **53**, 59–89 (2011).

[68] Oliveira, T., Gurgel, A. & Tonry, S. The effects of a linked carbon emissions trading scheme for Latin America. *Clim. Policy* **20**, 1–17 (2020).

[69] Ministerio de Relaciones Exteriores de Colombia. Consenso de Brasília. URL https://www.cancilleria.gov.co/international/consensus/consensobrasilia. Accessed: 2025-05-12 (2023).

[70] Inter-American Development Bank. Informe anual del Banco Interamericano de Desarrollo 2023: Reseña del año URL https://publications.iadb.org/publications/spanish/document/Informe-anual-del-Banco-Interamericano-de-Desarrollo-2023-Resena-del-ano.pdf. Accessed: 2025-05-12. (2024).

[71] CAF - Banco de Desarrollo de América Latina y el Caribe. Caf se consolida como el banco verde de América Latina y el Caribe. URL https://www.caf.com/es/actualidad/noticias/2024/02/caf-se-consolida-como-el-banco-verde-de-america-latina-y-el-caribe/ Accessed: 2025-05-12 (2024)

[72] Rodríguez, J. C. & Haag, V. The space left for regional integration (or lack thereof): Structural causes of institutional fragmentation in Latin America (1991–2019). *Revista Brasileira de Política Internacional* 10.1590/0034-7329202200111 (2022).

[73] Pierce, J. A South American energy treaty: How the region might attract foreign investment in a wake of resource nationalism. *Cornell Int. Law J.* **44**, 417 – 440 (2011).

[74] Mares, D. & Martin, J. Regional energy integration in Latin America: lessons from Chile’s experience with natural gas. *Third World Q.* **33**, 55–70 (2012).

[75] Zamora, A. Strategic implications of emerging market-oriented Latin American petroleum policies. *Energy Strategy Rev.* **3**, 55–62 10.1016/j.esr.2014.07.001 (2014).

[76] Puntigliano, A. R. "geopolitics of integration” and the imagination of south america. *Geopolitics* **16**, 846–864 (2011).

[77] Palestini, S. & Agostinis, G. Constructing regionalism in South America: the cases of sectoral cooperation on transport infrastructure and energy. *J. Int. Relat. Dev.* **21**, 46–74 10.1057/JIRD.2015.15 (2018).

[78] da Fonseca, L. M. The Latin American Energy Organization. *Energy* **6**, 861–877 10.1016/0360-5442(81)90129-8 (1981).

[79] Rosa, J. D. Economics, politics, and hydroelectric power: The paraná river basin. *Latin American Res. Rev*. 10.1017/s002387910002104x (1983).

[80] Blanc, J. Itaipu’s forgotten history: the 1965 act of Foz do iguaçu and the Itaipu project’s early legal and diplomatic controversies. *J. Lat. Am. Stud.* **49**, 295–321 (2017).

[81] Gwynn, M. A. International law and transboundary dams: lessons learned from the binational entity Itaipu (Brazil and Paraguay). *Front. Clim*. 10.3389/fclim.2023.1272254 (2023).

[82] Organización Latinoamericana de Energía (OLADE) and CAF – banco de desarrollo de América Latina y el Caribe. {Integración gasífera Argentina - Chile: Resumen ejecutivo. Tech. Rep., Organización Latinoamericana de Energía (OLADE) and CAF – banco de desarrollo de América Latina y el Caribe, Quito, Ecuador (2025).

[83] Llamosas, C., Upham, P. & Blanco, G. Multiple streams, resistance and energy policy change in Paraguay. *Energy Res. Soc. Sci.* **39**, 161–169 10.1016/j.erss.2018.03.011 (2018).

[84] Sanjurjo, D. Taking the multiple streams framework for a walk in Latin America. *Policy Sci.* **53**, 205–221 10.1007/s11077-020-09376-1 (2020).

[85] de Halleux, M., Estache, A. & Serebrisky, T. S. Governance choices and policy outcomes in the Latin American and Caribbean electricity sector. *Uti. Policy* **67**, 101105 10.1016/J.JUP.2020.101105 (2020).

[86] Kanai, J. M. The pervasiveness of neoliberal territorial design: Cross-border infrastructure planning in South America since the introduction of IIRSA. *Geoforum* **69**, 160–170 10.1016/J.GEOFORUM.2015.10.002 (2016).

[87] Reyes, C. V. in *Energy integration in South America and global geopolitics* (ed.Vivares, E.) *Regionalism, Development and the Post-Commodities Boom in South America* 147–164 (Springer International Publishing, Cham, 2018).

[88] Gonzalez-Salazar, M. & Poganietz, W. R. Making use of the complementarity of hydropower and variable renewable energy in Latin America: a probabilistic analysis. *Energy Strategy Rev.* **44**, 100972 (2022).

[89] Stríkis, N. M. et al. Modern anthropogenic drought in Central Brazil unprecedented during last 700 years. *Nat. Commun.* **15**, 1728 (2024). 10.1038/s41467-024-45469-8 PMC11258244 38409095

[90] Cai, W. et al. Climate impacts of the el niño-southern oscillation on south america. *Nat. Rev. Earth Environ.* **1**, 215–231 (2020).

[91] Cai, W. et al. Enso and greenhouse warming. *Nat. Clim. Change* **5**, 849–859 10.1038/nclimate2743 (2015).

[92] de Jong, P. et al. The impact of regional climate change on hydroelectric resources in South America. *Renew. Energy* **173**, 76–91 10.1016/J.RENENE.2021.03.077 (2021).

[93] de Jong, P. et al. Hydroelectric production from Brazil’s são francisco river could cease due to climate change and inter-annual variability. *Sci. Total Environ.* **634**, 1540–1553 10.1016/j.scitotenv.2018.03.256 (2018). 29710652

[94] Lucena, A. et al. The vulnerability of renewable energy to climate change in Brazil. *Energy Policy* **37**, 879–889 (2009).

[95] Carvajal, P. & Li, F. Challenges for hydropower-based nationally determined contributions: a case study for Ecuador. *Clim. Policy* **19**, 974–987 (2019).

[96] Pabón-Caicedo, J. D. et al. Observed and projected hydroclimate changes in the Andes. *Front. Earth Sci.* **8**, 61 (2020).

[97] Melo, D., Scanlon, B., Zhang, Z., Wendland, E. & Yin, L. Reservoir storage and hydrologic responses to droughts in the paraná river basin, south-eastern Brazil. *Hydrol. Earth Syst. Sci.* **20**, 4673–4688 10.5194/hess-20-4673-2016 (2016).

[98] Mideksa, T. K. & Kallbekken, S. The impact of climate change on the electricity market: a review. *Energy Policy* **38**, 3579–3585 10.1016/J.ENPOL.2010.02.035 (2010).

[99] Ochoa, C., Dyner, I. & Franco, C. J. Simulating power integration in Latin America to assess challenges, opportunities, and threats. *Energy Policy* **61**, 267–273 (2013).

[100] Agostini, C. A., Guzmán, A. M., Nasirov, S. & Silva, C. A surplus based framework for cross-border electricity trade in South America. *Energy Policy* **128**, 673–684 10.1016/j.enpol.2019.01.053 (2019).

[101] Chowdhury, A. F. M. K., Wessel, J., Wild, T., Lamontagne, J. & Kanyako, F. Exploring sustainable electricity system development pathways in South America’s MERCOSUR sub-region. *Energy Strategy Rev.* **49**, 101150 (2023).

[102] Hira, A. & Amaya, L. Does energy integrate. *Energy Policy* **31**, 185–199 (2003).

[103] Postic, S., Selosse, S. & Maïzi, N. Energy contribution to Latin American Indcs: Analyzing sub-regional trends with a times model. *Energy Policy* **101**, 170–184 10.1016/J.ENPOL.2016.11.023 (2017).

[104] De Oliveira-De Jesus, P. M., González De León, M., & Melán, R. A. A mitigation scenario for Latin American power-relatedcarbon dioxide emissions. *Energy Sources, Part B: Economics, Planning, and Policy* **15**, 492–512 10.1080/15567249.2020.1770374 (2022).

[105] Barbosa, L. S. N. S., Bogdanov, D., Vainikka, P. & Breyer, C. Hydro, wind and solar power as a base for a 100% renewable energy supply for South and Central America. *PLoS ONE* **12**, e0173820 10.1371/journal.pone.0173820 (2017). PMC5362076 28329023

[106] International Energy Agency (IEA). World energy investment outlook. Tech. Rep., Organisation for Economic Co-operation and Development (OECD). URL https://www.iea.org/reports/world-energy-investment-outlook-2024 (2024).

[107] Wessel, J. et al. Regional coordination can alleviate the cost burden of a low-carbon electricity system. *Nat. Commun.* **16**, 9033 (2025). 10.1038/s41467-025-64093-8 PMC12514305 41073444

[108] Organización Latinoamericana de Energía (OLADE). Situación de la integración eléctrica en América del Sur. Tech. Rep., OLADE, Quito, Ecuador (2024).

[109] Escribano, G., Lázaro-Touza, L. & Pardo, E. Renewable energies in Latin America: resources, public policies, and geopolitics. In *Handbook on the Geopolitics of the Energy Transition*. Edward Elgar Publishing, pp. 535–549 (2023).

[110] Guerrero, A. L. Geopolitics of global energy transformation and territorial dynamics of energy transition in South America. *Ambient. Soc.* **24**, e00263 (2021).

[111] Sabbatella, I. & Santos, T. The IPE of regional energy integration in South America. In *The Routledge Handbook to Global Political Economy*. Routledge, pp. 719–740 (2020).

[112] International Energy Agency. Global critical minerals outlook 2024. Tech. Rep., IEA, Paris URL https://www.iea.org/reports/global-critical-minerals-outlook-2024 Accessed: 2026-04-26 (2024).

[113] Comisión Económica para América Latina y el Caribe (CEPAL) Minerales críticos para la transición energética y la electromovilidad: oportunidades para el desarrollo económico con desafíos socioambientales. URL https://www.cepal.org/pt-br/node/64620 Accessed: 2026-04-26 (2024)

[114] Averchenkova, A., Lázaro-Touza, L. & Escribano, G. Beyond leading by example: enhanced EU-LAC climate cooperation—the case of Brazil, Chile and Mexico. *Int. Environ. Agreements: Politics, Law Econ.* **25**, 267–284 (2025).

[115] European External Action Service & European Commission. A new agenda for relations between the EU and Latin America and the Caribbean. URL https://www.eeas.europa.eu/eeas/new-agenda-relations-between-eu-and-latin-america-and-caribbean_en Accessed: 2026-04-26 (2023).

[116] European Commission. The EU-Mercosur trade agreement URL https://commission.europa.eu/topics/trade/eu-mercosur-trade-agreement_en. Last updated 2026-03-23; accessed: 2026-04-26 (2026).

[117] Council of the European Union. Advanced framework agreement between the European Union and its member states, of the one part, and the Republic of Chile, of the other part URL https://www.consilium.europa.eu/en/documents/treaties-agreements/agreement/?docLanguage=en&id=2023027. Official Journal references L, 2024/01758 and L, 2024/01759; accessed: 2026-04-26 (2024).

[118] The White House. National Security Strategy of the United States of America. Executive Office of the President, Washington, D.C. URL https://www.whitehouse.gov/wp-content/uploads/2025/12/2025-National-Security-Strategy.pdf. Accessed: 2026-04-28 (2025).

[119] Herweg, N., Huß, C. & Zohlnhöfer, R. Straightening the three streams: Theorising extensions of the multiple streams framework. *Eur. J. Political Res.* **54**, 435–449 (2015).

[120] Harvey, D. *The New Imperialism* (Oxford University Press, 2003).

[121] Dorn, F. M. Green colonialism in Latin America? towards a new research agenda for the global energy transition. *Eur. Rev. Lat. Am. Caribb. Stud.* **114**, 137–146 (2022).

[122] Gómez Lende, S. De la fractura metabólica a la acumulación por desposesión: minería del litio, imperialismo ecológico y despojo hídrico en el noroeste argentino. *Agua y. Territorio* **20**, 23–40 (2022).

[123] Colgan, J., Keohane, R. & Van de Graaf, T. Punctuated equilibrium in the energy regime complex. * Rev. Int. Organ.* **7**, 117–143 (2011).

[124] van den Dool, A. & Schlaufer, C. Policy process theories in autocracies: key observations, explanatory power, and research priorities. *Rev. Policy Res.* **41**, 865–891 (2024).

[125] Lin, J., Gao, C., Zeng, J. & Han, F. Stackelberg-Nash asymmetric bargaining-based scheduling optimization and revenue-allocation for multi-operator regional integrated energy system considering competition-cooperation relationship and source-load uncertainties. *Energy* **291**, 130262 (2024).

[126] Gallagher, K. Trading away the ladder? Trade politics and economic development in the Americas. *N. Political Econ.* **13**, 37–59 (2008).

[127] Ferreira, F., Castillo, T., Chamba, M., Salazar, G. & Añó, O. Integración eléctrica en Latinoamérica y el Caribe: Barreras y análisis de esquemas regulatorios. Tech. Rep. OLADE. Sitio web: http://biblioteca.olade.org (2012).

[128] Yin, R.*Case Study Research* Applied social research methods series (SAGE Publications, 2014). URL https://books.google.cl/books?id=Cdk5DQAAQBAJ.

[129] Organización Latinoamericana de Energía (OLADE). Panorama energético de América Latina y el Caribe 2024. Informe técnico, OLADE URL https://www.olade.org/publicaciones/panorama-energetico-de-america-latina-y-el-caribe-2024/ (2024).

[130] Ramírez, L. et al. Latamenergyintregrationexplorer (version v2). 10.5281/zenodo.19775192 (2026).

