# Supplementary material for: Climate urgency enables political alignment for Latin American power integration
Source: Nat Commun. 2026 Sep 17;17:10943. doi: 10.1038/s41467-026-77772-x (PMC13680428; doi:10.1038/s41467-026-77772-x)
Supplement: Supplementary file 1 [file 41467_2026_77772_MOESM1_ESM.pdf]

Supplementary Information for:

Climate urgency enables political alignment for Latin American power integration

Luis Ramírez[1,*], Gerardo Blanco[2,*], Felipe Sepulveda[3],
Luiz Barroso[4,5], Wilfredo C. Flores[6], Rodrigo Moreno[7]

[1] Departamento de Ingeniería Eléctrica, Universidad de Chile, Santiago, Chile
[2] Escuela de Ingeniería Eléctrica, Pontificia Universidad Católica de Valparaíso, Valparaíso, Chile
[3] Departamento de Ingeniería Industrial, Universidad de Chile, Santiago, Chile
[4] PSR, Río de Janeiro, Brasil
[5] Instituto de Investigación Tecnológica, Universidad Pontificia Comillas, Madrid, España
[6] Facultad de Ingeniería, Universidad Tecnológica Centroamericana, Tegucigalpa, Honduras
[7] Facultad de Ingeniería y Ciencias, y Escuela de Negocios, Universidad Adolfo Ibáñez, Santiago, Chile

*Corresponding authors: luis.ramirez.c@ug.uchile.cl; gerardo.blanco@pucv.cl

Contents

Supplementary Methods **Page**

Supplementary Methods. Optimization model 3

Supplementary Notes

Supplementary Note 1. Formulation of geopolitical costs and losses from trusting/not trusting 10

Supplementary Note 2. Cost assumptions and emission factors 12

Supplementary Note 3. Hydrological resource patterns in Latin America 14

Supplementary Note 4. Long-term cross-border transmission in Latin America . . . 17

Supplementary Note 5. Quantitative summary of emissions and costs 22

Supplementary Figures

Supplementary Figure 1. Normalized hourly hydropower generation profiles for selected Latin American countries 15

Supplementary Figure 2. Monthly hydropower-profile correlation matrix 16

Supplementary Figure 3. Existing cross-border electricity interconnections in Latin America by 2025 18

Supplementary Figure 4. Planned transmission interconnections and capacity expansions by 2035 (no sovereignty scenario) 19

Supplementary Figure 5. Planned transmission interconnections and capacity expansions by 2045 (no sovereignty scenario) 20

Supplementary Figure 6. Planned transmission interconnections and capacity expansions by 2035 (sovereignty scenario) 21

Supplementary Figure 7. Planned transmission interconnections and capacity expansions by 2045 (sovereignty scenario) 22

Supplementary Tables

Supplementary Table 1. Sets, indices, decision variables, and parameters used in the optimization model 3

Supplementary Table 2. Variable operating costs of thermal generators 12

Supplementary Table 3. Annualized investment costs by technology 12

Supplementary Table 4. Carbon dioxide emission factors for thermal technologies . 13

Supplementary Table 5. Existing and candidate transmission lines 13

Supplementary Table 6. Absolute carbon emissions for 2035 and 2045 23

Supplementary Table 7. Total operation and investment costs for 2035 and 2045 . 23

Supplementary References 24

Supplementary Methods

Optimization Model

The optimization model minimizes the total cost of electricity supply in Latin America by jointly optimizing investments in generation, storage, and transmission, along with system operation. The model is formulated as a linear program and captures hourly operation across a full representative year. It includes multiple technology types such as solar, wind, hydropower (both run-of-river and reservoir), nuclear, and diesel generation, as well as battery energy storage systems (BESS). Investment decisions are endogenously determined for dispatchable and variable renewable generation, BESS, and transmission lines. The model ensures hourly nodal balance, enforces technical constraints on generation and transmission, and penalizes unserved energy and artificial loop flows. Additionally, hydropower generation is constrained by historical production profiles and capacity limits, while sovereignty constraints can be imposed to ensure each country maintains a minimum level of firm generation to meet its peak demand. All data inputs—including demand, capacity factors, existing infrastructure, and cost assumptions—are derived from publicly available sources and regional planning reports.[1–3]

Model Notation

The sets, indices, decision variables, and parameters used in the optimization model are summarized in Supplementary Table 1. Unless otherwise stated, units are reported as megawatts (MW), megawatt-hours (MWh), hours (h), per unit (p.u.), and U.S. dollars (USD).

Supplementary Table 1: Sets, indices, decision variables, and parameters used in the optimization model. Abbreviations and units: BESS = battery energy storage system; CV = variable cost; CI = investment cost; ENS = energy not supplied; LOOP = loop-flow penalty; VOLL = value of lost load; MW = megawatt; MWh = megawatt-hour; h = hour; p.u. = per unit; USD = U.S. dollars.

Symbol	Meaning	Unit
**Sets and indices**		
$t \in T$	Time periods, typically hourly periods.	–
$b \in B$	Batteries.	–
$n \in N$	Nodes or countries.	–
$l \in L$	Transmission lines.	–
$g \in G^C$	Conventional dispatchable generators.	–
$s \in G^S$	Solar generators.	–
$w \in G^W$	Wind generators.	–
$r \in G^R$	Run-of-river hydropower plants.	–
$h \in G^H$	Reservoir-based hydropower plants.	–
$u \in G^N$	Nuclear generators.	–
$k \in K^{\text{firm}}$	Generation technologies or resources contributing to sovereignty.	–
B_n	Batteries located at node n.	–
G_n^C	Conventional dispatchable generators located at node n.	–

Continued on next page

Supplementary Table 1: Sets, indices, decision variables, and parameters used in the optimization model (continued). Abbreviations and units: BESS = battery energy storage system; CV = variable cost; CI = investment cost; ENS = energy not supplied; LOOP = loop-flow penalty; VOLL = value of lost load; MW = megawatt; MWh = megawatt-hour; h = hour; p.u. = per unit; USD = U.S. dollars.

Symbol	Meaning	Unit
G_n^S	Solar generators located at node n.	–
G_n^W	Wind generators located at node n.	–
G_n^R	Run-of-river hydropower plants located at node n.	–
G_n^H	Reservoir hydropower plants located at node n.	–
G_n^N	Nuclear generators located at node n.	–
L_n^{out}	Transmission lines originating at node n.	–
L_n^{in}	Transmission lines ending at node n.	–
K_n^{firm}	Firm generation technologies or plants contributing to sovereignty at node n.	–
T^+	Time periods excluding the first period, i.e., $T^+ = T \setminus \{t_1\}$.	–
**Variables**		
$P_{g,t}^C$	Power generation from conventional dispatchable generator g at hour t.	MW
$P_{s,t}^S$	Power generation from solar generator s at hour t.	MW
$P_{w,t}^W$	Power generation from wind generator w at hour t.	MW
$P_{r,t}^R$	Power generation from run-of-river hydropower plant r at hour t.	MW
$P_{h,t}^H$	Power generation from reservoir hydropower plant h at hour t.	MW
$P_{u,t}^N$	Power generation from nuclear generator u at hour t.	MW
$C_{b,t}^{\text{BESS}}$	Charging power of battery b at hour t.	MW
$D_{b,t}^{\text{BESS}}$	Discharging power of battery b at hour t.	MW
$P_{b,t}^{\text{BESS}}$	Net injection from battery b at hour t, positive when discharging and negative when charging.	MW
$E_{b,t}^{\text{BESS}}$	Energy stored in battery b at hour t.	MWh
$E_{h,t}^H$	Energy stored in reservoir h at hour t.	MWh
$\Delta_{n,t}$	Energy not supplied (ENS) in node n at hour t.	MW
$F_{l,t}$	Power flow on line l at hour t.	MW
$F_{l,t}^{\text{LOOP}}$	Auxiliary nonnegative variable for the loop-flow penalty (LOOP), used to penalize absolute line flows.	MW
I_g^C	New investment in conventional generation capacity g.	MW
I_s^S	New investment in solar capacity s.	MW
I_w^W	New investment in wind capacity w.	MW
I_b^{BESS}	New investment in BESS power capacity b.	MW
I_l^F	New transmission capacity investment in line l.	MW

Continued on next page

Supplementary Table 1: Sets, indices, decision variables, and parameters used in the optimization model (continued). Abbreviations and units: BESS = battery energy storage system; CV = variable cost; CI = investment cost; ENS = energy not supplied; LOOP = loop-flow penalty; VOLL = value of lost load; MW = megawatt; MWh = megawatt-hour; h = hour; p.u. = per unit; USD = U.S. dollars.

Symbol	Meaning	Unit
I_k	New investment in capacity of firm resource k; this is a notational alias rather than an independent decision variable. It is mapped to I_g^C, I_s^S, or I_w^W when expandable, and set to $I_k = 0$ for resources without investment decisions.	MW
**Parameters**		
$D_{n,t}$	Electricity demand at node n at hour t.	MW
D_n^{peak}	Peak demand at node n, defined as $D_n^{\text{peak}} = \max_{t \in T} D_{n,t}$.	MW
$\overline{P}_g^C$	Existing maximum capacity of conventional generator g.	MW
$\overline{P}_s^S$	Existing maximum capacity of solar generator s.	MW
$\overline{P}_w^W$	Existing maximum capacity of wind generator w.	MW
$\overline{P}_r^R$	Existing maximum capacity of run-of-river hydropower plant r.	MW
$\overline{P}_h^H$	Existing maximum capacity of reservoir hydropower plant h.	MW
$\overline{P}_u^N$	Existing maximum capacity of nuclear generator u.	MW
$\overline{F}_l$	Existing maximum transmission capacity of line l.	MW
$\overline{E}_h^H$	Maximum energy storage capacity of reservoir h.	MWh
$E_b^{\text{BESS,init}}$	Initial stored energy in BESS b.	MWh
$E_h^{H,\text{init}}$	Initial stored energy in reservoir h.	MWh
Hr_b	Duration of BESS storage b.	h
η_b^{ch}	Charging efficiency of BESS b.	p.u.
η_b^{dis}	Discharging efficiency of BESS b.	p.u.
$\phi_{s,t}^S$	Solar availability profile of generator s at hour t.	p.u.
$\phi_{w,t}^W$	Wind availability profile of generator w at hour t.	p.u.
$\phi_{r,t}^R$	Run-of-river generation profile of plant r at hour t.	p.u.
$\phi_{h,t}^H$	Normalized reservoir hydropower inflow profile of plant h at hour t.	p.u.
$\text{Inflow}_{h,t}$	Estimated inflow to reservoir h at hour t.	MWh
Prod_r^R	Historical annual generation of run-of-river plant r.	MWh
Prod_h^H	Historical annual generation of reservoir plant h.	MWh
α_k	Firm capacity contribution factor of firm resource k.	p.u.
CV_g^C	Variable operating cost of conventional generator g.	USD MWh^{-1}
CI_g^C	Investment cost of conventional generator g.	USD MW^{-1}
CI_s^S	Investment cost of solar generator s.	USD MW^{-1}
CI_w^W	Investment cost of wind generator w.	USD MW^{-1}
$\text{CI}_b^{\text{BESS}}$	Investment cost of BESS energy capacity b.	USD MWh^{-1}
CI_l^F	Investment cost of transmission line l.	USD MW^{-1}

Continued on next page

Supplementary Table 1: Sets, indices, decision variables, and parameters used in the optimization model (continued). Abbreviations and units: BESS = battery energy storage system; CV = variable cost; CI = investment cost; ENS = energy not supplied; LOOP = loop-flow penalty; VOLL = value of lost load; MW = megawatt; MWh = megawatt-hour; h = hour; p.u. = per unit; USD = U.S. dollars.

Symbol	Meaning	Unit
VOLL	Value of Lost Load; cost penalty per unit of unmet demand.	USD MWh^{-1}
λ	Small penalty factor for artificial loop flows.	USD MWh^{-1} or penalty unit
$\overline{P}_k$	Existing installed maximum capacity of firm resource k; it is a notational alias to $\overline{P}_g^C$, $\overline{P}_s^S$, $\overline{P}_w^W$, $\overline{P}_r^R$, $\overline{P}_h^H$, or $\overline{P}_u^N$, depending on the technology of k.	MW

Objective Function

The objective is to minimize the total system cost, which includes operating costs, investment costs, and penalties, as shown in Supplementary Equation (1).

The objective components are operating expenditures (OPEX), capital expenditures (CAPEX), generation-expansion costs (GX), transmission-expansion costs (TX), energy-not-supplied penalties (ENS), and loop-flow penalties (LOOP).

$$\begin{aligned}\min Z = \mathrm{OPEX} + \mathrm{CAPEX}^{\mathrm{GX}} + \mathrm{CAPEX}^{\mathrm{TX}} + \mathrm{CAPEX}^{\mathrm{BESS}} \\ + \mathrm{ENS} + \mathrm{LOOP}.\end{aligned} \tag{1}$$

The objective-function components are defined as follows: The corresponding formulas are presented in Supplementary Equations (2), (3), (4), (5), (6), and (7).

Operating Cost :

$$\mathrm{OPEX} = \sum_{t\in T}\sum_{g\in G^C} \mathrm{CV}_g^C \cdot P_{g,t}^C. \tag{2}$$

Generation Investment Cost ($\mathbf{CAPEX^{GX}}$):

$$\begin{aligned}\mathrm{CAPEX}^{\mathrm{GX}} = \sum_{g\in G^C} \mathrm{CI}_g^C \cdot I_g^C + \sum_{s\in G^S} \mathrm{CI}_s^S \cdot I_s^S \\ + \sum_{w\in G^W} \mathrm{CI}_w^W \cdot I_w^W.\end{aligned} \tag{3}$$

Transmission Investment Cost ($\mathbf{CAPEX^{TX}}$):

$$\mathrm{CAPEX}^{\mathrm{TX}} = \sum_{l\in L} \mathrm{CI}_l^F \cdot I_l^F. \tag{4}$$

Storage Investment Cost ($\mathbf{CAPEX^{BESS}}$):

$$\mathrm{CAPEX}^{\mathrm{BESS}} = \sum_{b \in B} \mathrm{CI}_b^{\mathrm{BESS}} \cdot \left(I_b^{\mathrm{BESS}} \cdot \mathrm{Hr}_b \right). \tag{5}$$

Unserved Energy Penalty (ENS):

$$\mathrm{ENS} = \sum_{t \in T} \sum_{n \in N} \mathrm{VOLL} \cdot \Delta_{n,t}. \tag{6}$$

Loop Flow Penalty :

$$\mathrm{LOOP} = \sum_{t \in T} \sum_{l \in L} \lambda \cdot F_{l,t}^{\mathrm{LOOP}}. \tag{7}$$

Where λ is a small penalty factor (e.g., 0.001) used to discourage artificial loop flows.

Model Constraints

Nodal Power Balance

For each node $n \in N$ and time $t \in T$, the sum of generation, battery net injection, imports, and unserved energy must match demand and exports, as imposed by Supplementary Equation (8):

$$\begin{aligned}
&\sum_{g \in G_n^C} P_{g,t}^C + \sum_{s \in G_n^S} P_{s,t}^S + \sum_{w \in G_n^W} P_{w,t}^W \\
&\quad + \sum_{r \in G_n^R} P_{r,t}^R + \sum_{h \in G_n^H} P_{h,t}^H + \sum_{u \in G_n^N} P_{u,t}^N \\
&\quad + \sum_{b \in B_n} P_{b,t}^{\mathrm{BESS}} + \Delta_{n,t} + \sum_{l \in L_n^{\mathrm{in}}} F_{l,t} \\
&= D_{n,t} + \sum_{l \in L_n^{\mathrm{out}}} F_{l,t} \qquad \forall n \in N,\ \forall t \in T.
\end{aligned} \tag{8}$$

Generation Limits

The generation bounds are stated in Supplementary Equations (9)–(13).

$$P_{g,t}^C \geq 0 \qquad \forall g \in G^C,\ \forall t \in T; \tag{9}$$

$$P_{g,t}^C \leq \overline{P}_g^C + I_g^C \qquad \forall g \in G^C,\ \forall t \in T; \tag{10}$$

$$P_{s,t}^S \leq (\overline{P}_s^S + I_s^S) \cdot \phi_{s,t}^S \qquad \forall s \in G^S,\ \forall t \in T; \tag{11}$$

$$P_{w,t}^W \leq (\overline{P}_w^W + I_w^W) \cdot \phi_{w,t}^W \qquad \forall w \in G^W,\ \forall t \in T; \tag{12}$$

$$P_{u,t}^N \leq \overline{P}_u^N \qquad \forall u \in G^N,\ \forall t \in T. \tag{13}$$

All generation variables are nonnegative, as stated explicitly in the variable-domain conditions.

Transmission Capacity Limits

Transmission capacities are constrained by Supplementary Equations (14)–(15).

$$F_{l,t} \le \overline{F}_l + I_l^F \qquad \forall l \in L,\ \forall t \in T; \tag{14}$$
$$F_{l,t} \ge -(\overline{F}_l + I_l^F) \qquad \forall l \in L,\ \forall t \in T. \tag{15}$$

Battery Operation

Battery charging, discharging, storage balance, and terminal conditions are represented by Supplementary Equations (16)–(23).

$$P_{b,t}^{\text{BESS}} = D_{b,t}^{\text{BESS}} - C_{b,t}^{\text{BESS}} \qquad \forall b \in B,\ \forall t \in T; \tag{16}$$
$$0 \le C_{b,t}^{\text{BESS}} \le I_b^{\text{BESS}} \qquad \forall b \in B,\ \forall t \in T; \tag{17}$$
$$0 \le D_{b,t}^{\text{BESS}} \le I_b^{\text{BESS}} \qquad \forall b \in B,\ \forall t \in T; \tag{18}$$
$$E_{b,t}^{\text{BESS}} = E_{b,t-1}^{\text{BESS}} + \eta_b^{\text{ch}} C_{b,t}^{\text{BESS}} - \frac{1}{\eta_b^{\text{dis}}} D_{b,t}^{\text{BESS}} \qquad \forall b \in B,\ \forall t \in T^+; \tag{19}$$
$$E_{b,t_1}^{\text{BESS}} = E_b^{\text{BESS,init}} + \eta_b^{\text{ch}} C_{b,t_1}^{\text{BESS}} - \frac{1}{\eta_b^{\text{dis}}} D_{b,t_1}^{\text{BESS}} \qquad \forall b \in B; \tag{20}$$
$$0 \le E_{b,t}^{\text{BESS}} \le I_b^{\text{BESS}} \cdot \text{Hr}_b \qquad \forall b \in B,\ \forall t \in T; \tag{21}$$
$$-I_b^{\text{BESS}} \le P_{b,t}^{\text{BESS}} \le I_b^{\text{BESS}} \qquad \forall b \in B,\ \forall t \in T; \tag{22}$$
$$E_{b,|T|}^{\text{BESS}} = E_b^{\text{BESS,init}} \qquad \forall b \in B. \tag{23}$$

Unserved Energy and Loop Flow Penalties

The unserved-energy and loop-flow penalty variables are bounded by Supplementary Equations (24)–(26).

$$0 \le \Delta_{n,t} \le D_{n,t} \qquad \forall n \in N,\ \forall t \in T; \tag{24}$$
$$F_{l,t}^{\text{LOOP}} \ge F_{l,t} \qquad \forall l \in L,\ \forall t \in T; \tag{25}$$
$$F_{l,t}^{\text{LOOP}} \ge -F_{l,t} \qquad \forall l \in L,\ \forall t \in T. \tag{26}$$

Run-of-River Plants

If an hourly profile is available, run-of-river generation is bounded by Supplementary Equation (27):

$$0 \le P_{r,t}^R \le \text{Prod}_r^R \cdot \phi_{r,t}^R \qquad \forall r \in G^R,\ \forall t \in T. \tag{27}$$

If no hourly profile is available and only historical annual production is used, run-of-river generation is bounded by Supplementary Equation (28):

$$P_{r,t}^R \le \overline{P}_r^R \cdot \left(\frac{\text{Prod}_r^R}{\overline{P}_r^R \cdot 8760} \right) \qquad \forall r \in G^R,\ \forall t \in T. \tag{28}$$

The profile $\phi_{r,t}^R$ is normalized so that $\sum_{t \in T} \phi_{r,t}^R = 1$.

Reservoir Plants (without profile)

Reservoir plants without hourly profiles are constrained by Supplementary Equations (29)–(30).

$$P^H_{h,t} \leq \overline{P}^H_h \qquad \forall h \in G^H,\ \forall t \in T; \tag{29}$$

$$\sum_{t \in T} P^H_{h,t} \leq \overline{P}^H_h \cdot |T| \cdot \left(\frac{\text{Prod}^H_h}{\overline{P}^H_h \cdot 8760} \right) \qquad \forall h \in G^H. \tag{30}$$

Reservoir Plants (with profile)

Reservoir plants with hourly profiles are constrained by Supplementary Equations (31)–(35).

$$E^H_{h,t} = E^H_{h,t-1} + \text{Inflow}_{h,t} - P^H_{h,t} \qquad \forall h \in G^H,\ \forall t \in T^+; \tag{31}$$

$$E^H_{h,t_1} = E^{H,\text{init}}_h + \text{Inflow}_{h,t_1} - P^H_{h,t_1} \qquad \forall h \in G^H; \tag{32}$$

$$\text{Inflow}_{h,t} = \text{Prod}^H_h \cdot \phi^H_{h,t} \qquad \forall h \in G^H,\ \forall t \in T; \tag{33}$$

$$P^H_{h,t} \leq \overline{P}^H_h \qquad \forall h \in G^H,\ \forall t \in T; \tag{34}$$

$$0 \leq E^H_{h,t} \leq \overline{E}^H_h \qquad \forall h \in G^H,\ \forall t \in T. \tag{35}$$

The reservoir inflow profile $\phi^H_{h,t}$ is normalized so that $\sum_{t \in T} \phi^H_{h,t} = 1$.

Energy Sovereignty Constraint

The peak demand parameter is defined in Supplementary Equation (36):

$$D^{\text{peak}}_n = \max_{t \in T} D_{n,t} \qquad \forall n \in N. \tag{36}$$

The energy-sovereignty requirement is imposed by Supplementary Equation (37):

$$\sum_{k \in K^{\text{firm}}_n} (\overline{P}_k + I_k)\alpha_k \geq D^{\text{peak}}_n \qquad \forall n \in N. \tag{37}$$

Here, K^{firm}_n includes the technologies considered firm at node n, such as thermal, nuclear, reservoir hydro, or any other firm resource. If a technology does not have an investment decision, set $I_k = 0$.

Variable Domains

For completeness, the variable-domain conditions are stated in Supplementary Equations (38)–(41):

$$P^C_{g,t},\ P^S_{s,t},\ P^W_{w,t},\ P^R_{r,t},\ P^H_{h,t},\ P^N_{u,t} \geq 0, \tag{38}$$

$$C^{\text{BESS}}_{b,t},\ D^{\text{BESS}}_{b,t},\ E^{\text{BESS}}_{b,t},\ \Delta_{n,t},\ F^{\text{LOOP}}_{l,t},\ E^H_{h,t} \geq 0, \tag{39}$$

$$I^C_g,\ I^S_s,\ I^W_w,\ I^{\text{BESS}}_b,\ I^F_l \geq 0, \tag{40}$$

$$P^{\text{BESS}}_{b,t},\ F_{l,t} \in \mathbb{R}. \tag{41}$$

Supplementary Note 1: Formulation of Geopolitical Costs and Losses from Trusting/Not Trusting

This Supplementary Note details the mathematical formulation of the political costs associated with regional energy integration in Latin America. Specifically, we operationalize and quantify three core metrics presented in the main text: (1) the Geopolitical Cost of sovereignty preferences, (2) the Economic Loss from Trusting, and (3) the Economic Loss from Not Trusting. These constructs enable the translation of political dynamics—such as fragmentation, distrust, and risk aversion—into quantifiable economic impacts across scenarios with and without sovereignty constraints (SE).

Geopolitical Cost (GC)

The Geopolitical Cost captures the economic inefficiency that arises when sovereignty constraints prevent optimal regional coordination. It is defined as the difference in total system cost between a scenario with sovereignty constraints (CSE), in which countries must maintain sufficient national firm capacity to meet peak demand, and a scenario without such constraints (SSE), which allows full regional cooperation. Formally, this difference reflects the additional cost of forgoing regional synergies in favor of a more fragmented configuration. In the article, this metric quantifies the cost of prioritizing energy sovereignty over system-wide efficiency.

Here, SSE denotes the scenario without sovereignty constraints, and CSE denotes the scenario with sovereignty constraints. The cost differences and resulting geopolitical-cost metric are defined in Supplementary Equations (42)–(44).

$$\Delta\text{OPEX} = \text{OPEX}^{(\text{CSE})} - \text{OPEX}^{(\text{SSE})}, \tag{42}$$

$$\Delta\text{CAPEX} = \text{CAPEX}^{(\text{CSE})} - \text{CAPEX}^{(\text{SSE})}, \tag{43}$$

$$\text{GC} = \Delta\text{OPEX} + \Delta\text{CAPEX}. \tag{44}$$

The resulting GC value quantifies the annual economic penalty from underutilizing regional synergies due to sovereignty-driven planning. As shown in Fig. 4 of the main article, this cost can reach up to 7.9 billion USD annually by 2045, depending on hydrological conditions.

Economic Loss from Trusting (ELT)

This metric captures the risk exposure of a country that opts to rely on regional integration and, as a consequence, reduces part of its local investments in generation and storage under the assumption that the integrated system will perform reliably. If this trust is unmet due to underperformance or lack of coordination, countries may face energy shortages and costly emergency responses. This metric is constructed from the differences in local investments and energy exchanges between coordinated and non-coordinated scenarios, valuing the cost of covering deficits through emergency technologies. Its objective is not to question the efficiency of integration, but to capture the risk perceived by countries that may consider high dependence on the regional system too costly.

Power Deficit (DP)

Here, CT denotes the coordinated-transmission scenario and ST denotes the status quo scenario.

$$\mathrm{DP}_n = P_n^{\mathrm{GX\&BESS,(ST)}} - P_n^{\mathrm{GX\&BESS,(CT)}}, \qquad \forall n \in N. \tag{45}$$

The definition in Supplementary Equation (45) measures the reduction in local capacity investments (generation and storage) due to reliance on regional imports.

Energy Deficit (DE)

Here, IMP denotes imports and EXP denotes exports.

$$\mathrm{DE}_n = \left(\mathrm{IMP}_n^{\mathrm{(CT)}} - \mathrm{IMP}_n^{\mathrm{(ST)}}\right) + \left(\mathrm{EXP}_n^{\mathrm{(ST)}} - \mathrm{EXP}_n^{\mathrm{(CT)}}\right), \qquad \forall n \in N. \tag{46}$$

DE_n is defined in Supplementary Equation (46), which captures the net change in electricity imports (IMP) and exports (EXP) for country n between the ST and CT scenarios.

Economic Loss from Trusting (ELT)

$$\begin{aligned}\mathrm{ELT}_n = {} & \mathrm{DE}_n \cdot C^{\mathrm{oil}} + \mathrm{CAPEX}^{\mathrm{GX,oil}}(\mathrm{DP}_n) \\ & - \left(\mathrm{CAPEX}_n^{\mathrm{GX\&BESS,(ST)}} - \mathrm{CAPEX}_n^{\mathrm{GX\&BESS,(CT)}}\right), \qquad \forall n \in N. \end{aligned} \tag{47}$$

Where:

C^{oil}: operating cost of emergency diesel generation,
$\mathrm{CAPEX}^{\mathrm{GX,oil}}(\mathrm{DP}_n)$: investment cost in diesel capacity equivalent to DP_n,
CT: coordinated transmission scenario (regional optimization),
ST: status quo scenario (limited or no regional coordination).

The formulation in Supplementary Equation (47) reflects the backup cost borne by a country if regional support fails. Countries with smaller internal systems (e.g., Uruguay, Bolivia) are particularly vulnerable, as illustrated in Fig. 5 of the article.

Note: Calculations assume no sovereignty constraints (SE) and exclude transmission investments.

Economic Loss from Not Trusting (ELNT)

The third metric corresponds to the Economic Loss from Not Trusting, which represents the opportunity cost of remaining outside an efficient regional integration. This metric measures the excess investment and operating costs incurred by a country for maintaining an isolated strategy relative to a scenario in which it fully exploits regional complementarities. In economic terms, it captures how much it costs each country to sustain a self-sufficiency strategy when a more efficient regional alternative exists. This metric is particularly useful for identifying which countries have the strongest objective incentives to promote regional integration.

$$
\begin{aligned}
\mathrm{ELNT}_n = & \left(\mathrm{CAPEX}_n^{\mathrm{GX\&BESS,(CSE)}} - \mathrm{CAPEX}_n^{\mathrm{GX\&BESS,(SSE)}}\right) \\
& + \left(\mathrm{OPEX}_n^{\mathrm{(CSE)}} - \mathrm{OPEX}_n^{\mathrm{(SSE)}}\right), \qquad \forall n \in N. \qquad (48)
\end{aligned}
$$

ELNT_n is defined in Supplementary Equation (48), which quantifies the additional capital and operating costs incurred due to lack of integration.

Note: All scenarios exclude future transmission investments in Latin America.

Together, these three metrics translate the discussion of trust, sovereignty, and cooperation into concrete economic outcomes, aligning with the need to evaluate not only aggregate benefits but also the distribution of risks and costs across countries.

Supplementary Note 2: Cost Assumptions and Emission Factors

The model incorporates technology-specific assumptions for variable costs, annualized investment costs, and carbon emission factors. Supplementary Table 2 presents the operating costs for thermal power plants. Supplementary Table 3 summarizes the annual investment costs per megawatt of installed capacity, and Supplementary Table 4 lists the carbon dioxide (CO_2) emission factors for thermal technologies. All cost data are expressed in 2025 USD.[4]

Supplementary Table 2: Variable operating costs of thermal generators (USD = U.S. dollars; MWh = megawatt-hour).

Technology	Variable Cost	Unit
Coal	46	USD MWh^{-1}
Diesel	200	USD MWh^{-1}
Gas	91	USD MWh^{-1}

Supplementary Table 3: Annualized investment costs by technology (PV = photovoltaic; USD = U.S. dollars; MW = megawatt; MWh = megawatt-hour; yr = year; km = kilometer).

Technology	Investment Cost	Unit
Gas	113000	USD MW^{-1} yr^{-1}
Diesel	62000	USD MW^{-1} yr^{-1}
Solar photovoltaic (PV)	80700	USD MW^{-1} yr^{-1}
Wind	115100	USD MW^{-1} yr^{-1}
Battery Storage	30000	USD MWh^{-1} yr^{-1}
Transmission*	30000	USD MW^{-1} yr^{-1}

Note: * 300 kilometers (km) assumed

Supplementary Table 4: Carbon dioxide (CO_2) emission factors for thermal technologies (tCO_2 = tonnes of carbon dioxide; MWh = megawatt-hour).

**Technology**	**Emission Factor**	**Unit**
Coal	0.949	tCO_2 MWh^{-1}
Gas	0.436	tCO_2 MWh^{-1}
Diesel	0.779	tCO_2 MWh^{-1}

Supplementary Table 5 presents the set of international transmission lines considered in the model, distinguishing between existing infrastructure and candidate projects. Existing lines represent the current cross-border interconnections with reported transfer capacities ($F_{\max}$), based on regional planning documents and system operator data.[5] Candidate lines are hypothetical interconnections with no predefined capacity, representing strategic opportunities for future regional integration. These candidate links are incorporated into the optimization model as potential investment options and can be selected endogenously depending on their economic and operational relevance. All transmission capacities are expressed in MW, and line directions are nominal and assumed bidirectional.

Supplementary Table 5: Existing and candidate transmission lines ($F_{\max}$ = maximum transfer capacity; MW = megawatt).

**From**	**To**	**Type**	$F_{\max}$ **(MW)**
Costa Rica	Panama	Existing	300
Brazil	Uruguay	Existing	570
Brazil	Argentina	Existing	2250
Paraguay	Argentina	Existing	3200
Paraguay	Brazil	Existing	6300
Uruguay	Argentina	Existing	3990
Argentina	Chile	Existing	200
Argentina	Bolivia	Existing	120
Peru	Ecuador	Existing	100
Ecuador	Colombia	Existing	613
Colombia	Venezuela	Existing	13.5
Mexico	Guatemala	Existing	200
Honduras	Nicaragua	Existing	300
El Salvador	Honduras	Existing	300
Nicaragua	Costa Rica	Existing	300
Guatemala	El Salvador	Existing	300
Panama	Colombia	Candidate	–
Brazil	Paraguay	Candidate	–
Brazil	Bolivia	Candidate	–
Venezuela	Brazil	Candidate	–
French Guiana	Brazil	Candidate	–
Brazil	Guyana	Candidate	–
Paraguay	Bolivia	Candidate	–
Peru	Chile	Candidate	–
Peru	Bolivia	Candidate	–
Chile	Bolivia	Candidate	–
Suriname	French Guiana	Candidate	–
Guyana	Suriname	Candidate	–

Supplementary Note 3: Hydrological Resource Patterns in Latin America

Hydropower plays a central role in Latin America's electricity systems. However, the seasonal and interannual variability of water inflows differs substantially across countries due to geographic and climatic diversity.[6] Understanding these temporal patterns is essential for regional planning, particularly when designing transmission interconnections and evaluating complementarities between hydro-dominated systems.

Normalized Hydro Generation Profiles

Supplementary Figure 1 presents the normalized hourly hydro generation profiles used in the model, grouped by region and country. Each curve corresponds to the median historical series for a given country, and all profiles have been scaled such that the sum of their hourly values over the full year (8,760 hours) equals 1. This normalization allows for a consistent comparison of seasonal and diurnal patterns across countries and regions, independently of their absolute generation levels. The Southern Cone and Andean regions are shown separately, including high and low scenarios for selected countries (e.g., Brazil, Paraguay, Colombia, and Ecuador) to reflect interannual hydrological variability.

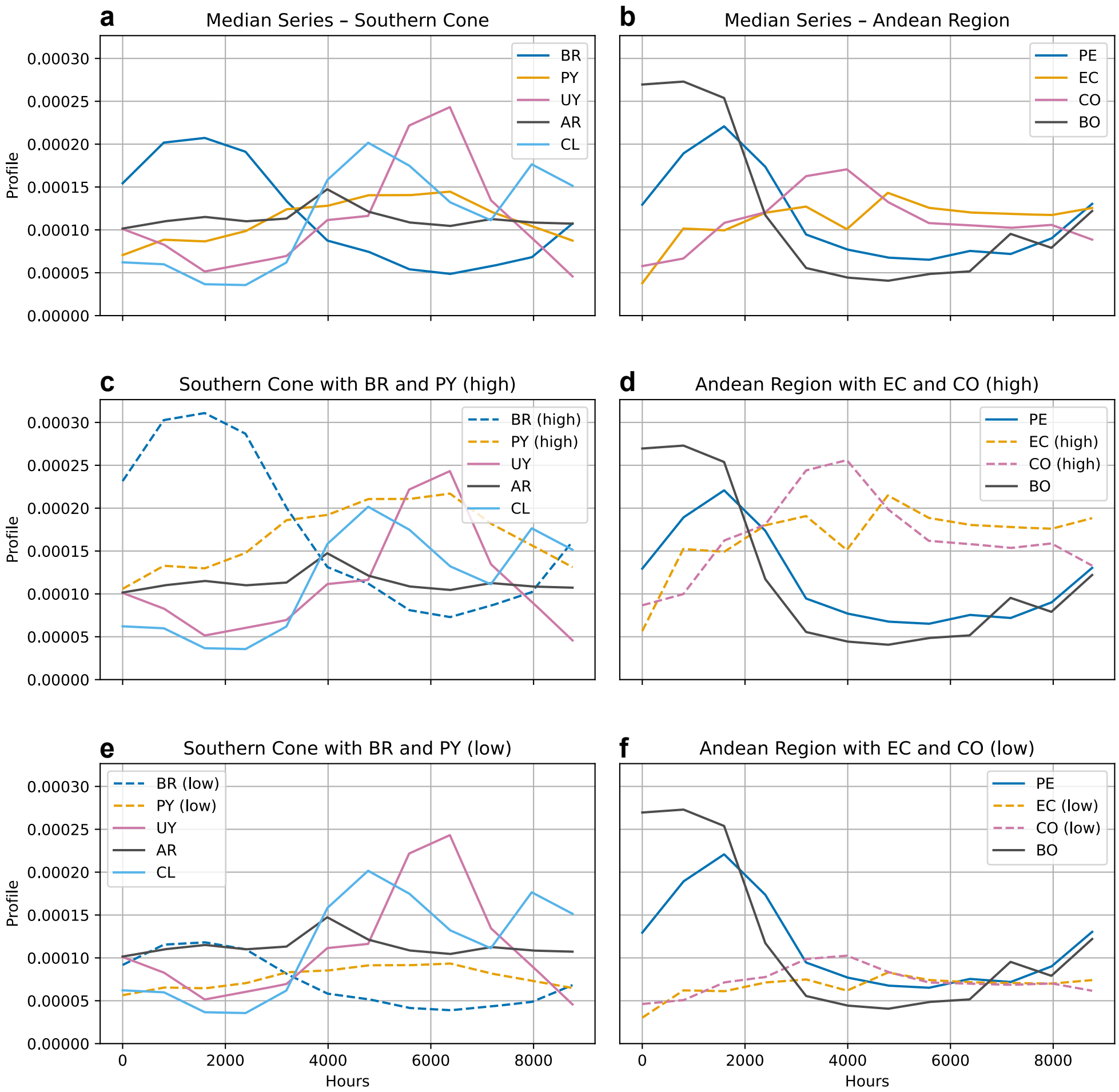

Supplementary Figure 1: Normalized hourly hydropower generation profiles for selected Latin American countries. Profiles are normalized so that the sum of hourly values over the full year (8,760 h) equals 1, allowing comparison of seasonal and diurnal patterns independently of absolute generation levels. **a** Median historical series for the Southern Cone (AR, BR, CL, PY, UY). **b** Median historical series for the Andean region (BO, CO, EC, PE). **c** Southern Cone profiles under high hydrological availability for Brazil and Paraguay, shown as dashed lines, with the median series retained for the remaining countries. **d** Andean profiles under high hydrological availability for Ecuador and Colombia, shown as dashed lines, with the median series retained for Bolivia and Peru. **e** As in **c**, but under low hydrological availability for Brazil and Paraguay. **f** As in **d**, but under low hydrological availability for Ecuador and Colombia. In all panels the horizontal axis denotes hours of the year and the vertical axis the normalized profile (p.u.). Country codes: AR = Argentina; BO = Bolivia; BR = Brazil; CL = Chile; CO = Colombia; EC = Ecuador; PE = Peru; PY = Paraguay; UY = Uruguay.

Hydrological Profile Correlation Analysis

To evaluate the synchronicity and complementarity of hydroelectric production profiles across South American countries, we computed a weighted correlation matrix based on normalized monthly profiles. Each correlation value reflects not only the temporal similarity (positive or negative correlation) between countries but also accounts for the relative magnitude of their expected production. This allows us to identify regions with aligned seasonal behavior as well as those with inverse or offset patterns, which can inform regional integration and diversification strategies.

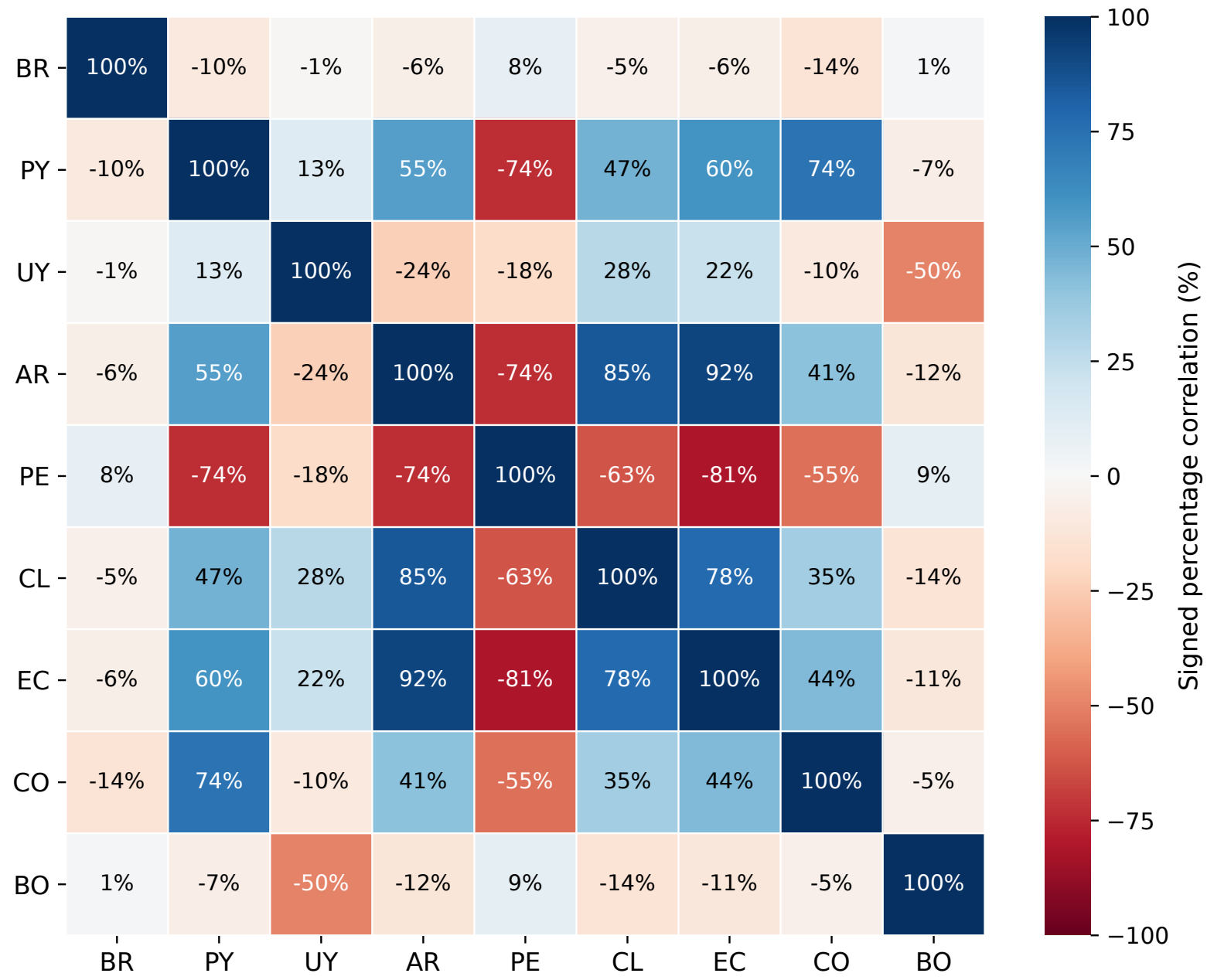

Supplementary Figure 2: Signed percentage correlation matrix based on monthly hydropower production profiles and their annual expected output. Country codes: AR = Argentina; BO = Bolivia; BR = Brazil; CL = Chile; CO = Colombia; EC = Ecuador; PE = Peru; PY = Paraguay; UY = Uruguay.

Country codes denote Argentina (AR), Bolivia (BO), Brazil (BR), Chile (CL), Colombia (CO), Ecuador (EC), Paraguay (PY), Peru (PE), and Uruguay (UY).

The matrix reveals several important regional patterns. High positive correlations are observed among Argentina, Chile, and Ecuador (e.g., AR–CL: +85%, AR–EC: +92%), indicating synchronous hydrological behavior. These countries experience similar seasonal patterns, which may limit the benefits of seasonal balancing but could enable shared reserve strategies. At the same time, strong negative correlations emerge between Peru and other countries, notably Argentina (–74%) and Ecuador (–81%). This reflects a high degree of seasonal complementarity and suggests that power exchange between the Andean and Southern Cone regions could enhance system flexibility. Paraguay shows intermediate alignment with Colombia (+74%), Ecuador (+60%), and Argentina (+55%), pointing to a moderately synchronous but potentially flexible role in regional integration, while Brazil and Bolivia exhibit low correlation

with most other countries (e.g., BR–UY: –1%, BO–AR: –12%), suggesting hydrological independence or divergent seasonality.

These insights highlight potential opportunities for regional interconnection strategies, particularly between hydrologically complementary areas such as Peru and the Southern Cone, or Colombia and Argentina.

Supplementary Note 4: Long-Term Evolution of Cross-Border Transmission in Latin America

Supplementary Figures 3, 4, and 5 detail the existing interconnections in 2025 and the planned transmission expansions by 2035 and 2045 under the scenario without energy sovereignty.[5] Between 2025 and 2045, the region's cross-border transmission capacity grows exponentially — transitioning from a limited set of links mostly under 4,000 MW in 2025, to a significantly more robust grid by 2035, with multiple corridors exceeding 15,000 MW. Examples include Colombia–Panama (15,533 MW) and Venezuela–Colombia (20,482 MW). By 2045, some links reach and surpass 30,000 MW, such as Venezuela–Colombia (32,618 MW) and Venezuela–Brazil (32,788 MW).

This expansion reflects a strategic shift from a fragmented and bilateral system toward a fully meshed, resilient regional grid designed to enable the large-scale exchange of renewable electricity, enhance energy security, and reduce the geopolitical costs of sovereignty-based constraints. Notably, investment costs for interconnections in this scenario amount to approximately 6.51 billion USD by 2035, increasing by 2 billion USD by 2045.

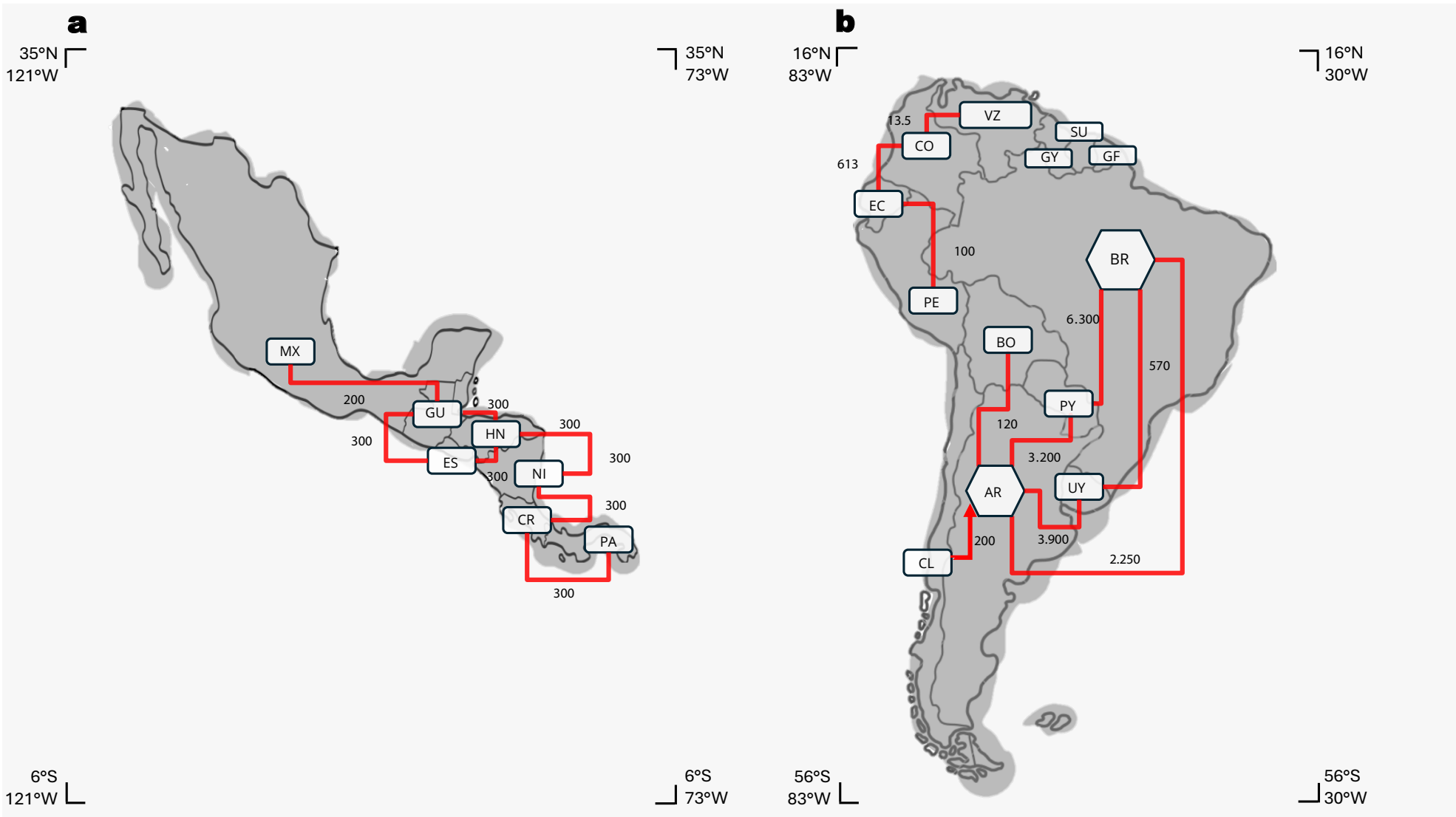

Supplementary Figure 3: Existing cross-border electricity interconnections in Latin America by 2025 (base case, without sovereignty constraints). **a** Mexico and Central America, covering the extent 35°N–6°S and 121°W–73°W. **b** South America, covering the extent 16°N–56°S and 83°W–30°W. In both panels, red lines denote existing cross-border interconnections and the adjacent numbers indicate their maximum transfer capacity in MW (megawatts), with all links assumed bidirectional. Corner labels report geographic coordinates (latitude, longitude) referenced to the World Geodetic System 1984 datum (WGS 84, EPSG:4326); base maps are displayed in the geographic (Plate Carrée) projection. Country codes: AR = Argentina; BO = Bolivia; BR = Brazil; CL = Chile; CO = Colombia; CR = Costa Rica; EC = Ecuador; ES = El Salvador; GF = French Guiana; GU = Guatemala; GY = Guyana; HN = Honduras; MX = Mexico; NI = Nicaragua; PA = Panama; PE = Peru; PY = Paraguay; SU = Suriname; UY = Uruguay; VZ = Venezuela.

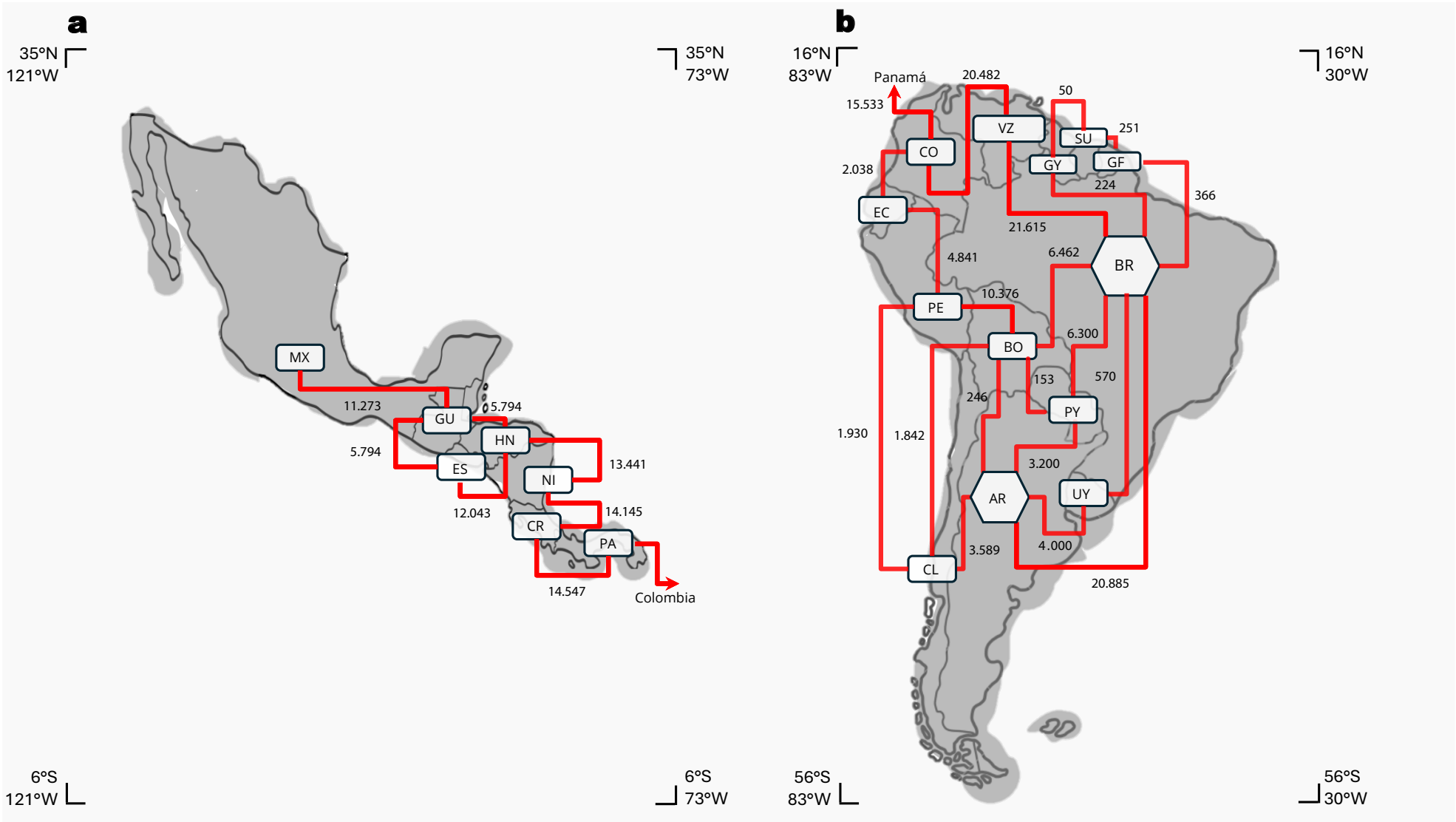

Supplementary Figure 4: Planned transmission interconnections and capacity expansions by 2035 (no sovereignty scenario). **a** Mexico and Central America, covering the extent 35°N–6°S and 121°W–73°W. **b** South America, covering the extent 16°N–56°S and 83°W–30°W. In both panels, red lines denote cross-border interconnections and the adjacent numbers indicate the resulting total transfer capacity in MW (megawatts) after the modelled expansion; line directions are nominal and all links are assumed bidirectional. Corner labels report geographic coordinates (latitude, longitude) referenced to the World Geodetic System 1984 datum (WGS 84, EPSG:4326); base maps are displayed in the geographic (Plate Carrée) projection and panels are not drawn to a common scale. Country codes: AR = Argentina; BO = Bolivia; BR = Brazil; CL = Chile; CO = Colombia; CR = Costa Rica; EC = Ecuador; ES = El Salvador; GF = French Guiana; GU = Guatemala; GY = Guyana; HN = Honduras; MX = Mexico; NI = Nicaragua; PA = Panama; PE = Peru; PY = Paraguay; SU = Suriname; UY = Uruguay; VZ = Venezuela.

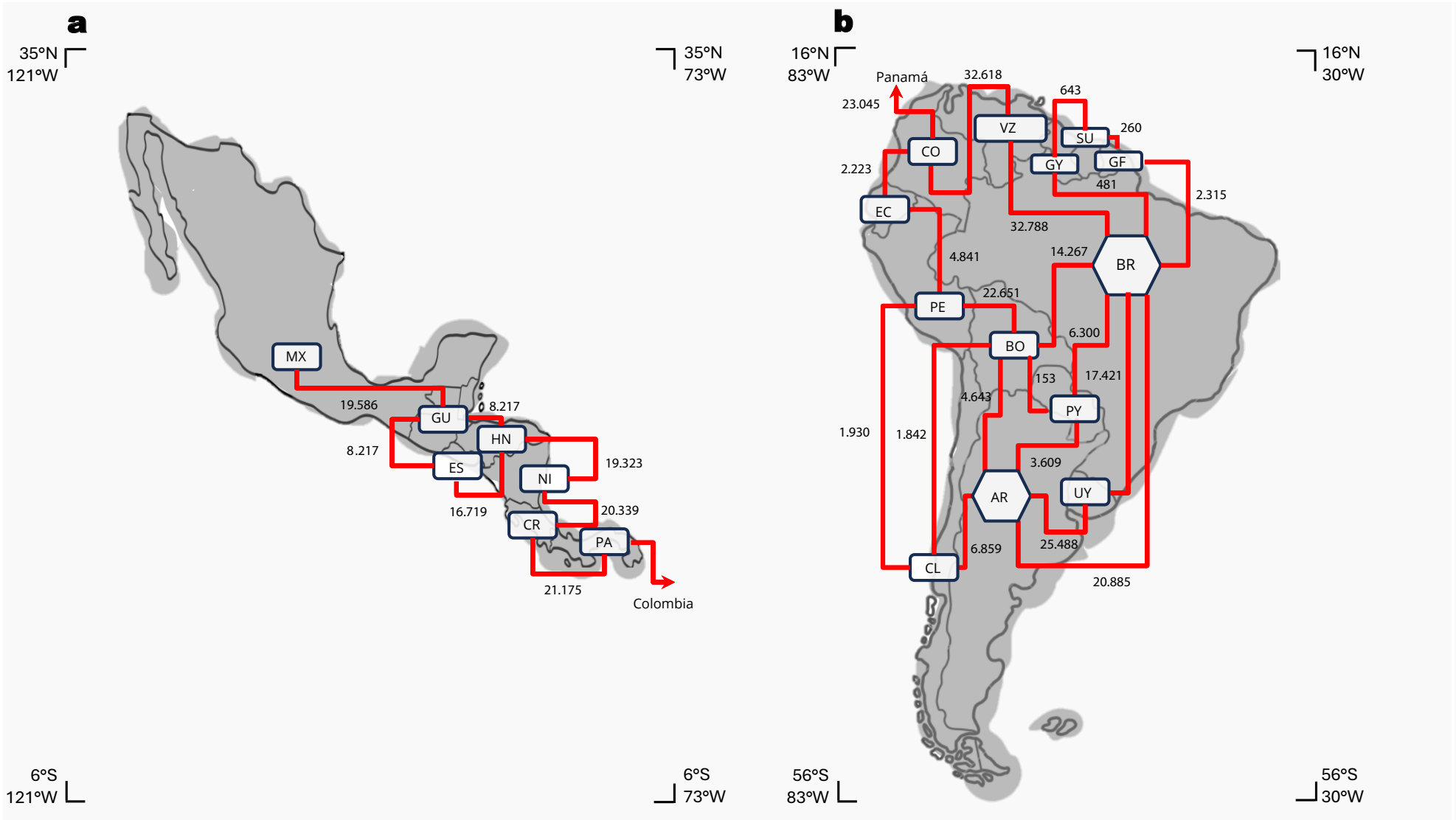

Supplementary Figure 5: Planned transmission interconnections and capacity expansions by 2045 (no sovereignty scenario). **a** Mexico and Central America, covering the extent 35°N–6°S and 121°W–73°W. **b** South America, covering the extent 16°N–56°S and 83°W–30°W. In both panels, red lines denote cross-border interconnections and the adjacent numbers indicate the resulting total transfer capacity in MW (megawatts) after the modelled expansion; line directions are nominal and all links are assumed bidirectional. Corner labels report geographic coordinates (latitude, longitude) referenced to the World Geodetic System 1984 datum (WGS 84, EPSG:4326); base maps are displayed in the geographic (Plate Carrée) projection and panels are not drawn to a common scale. Country codes: AR = Argentina; BO = Bolivia; BR = Brazil; CL = Chile; CO = Colombia; CR = Costa Rica; EC = Ecuador; ES = El Salvador; GF = French Guiana; GU = Guatemala; GY = Guyana; HN = Honduras; MX = Mexico; NI = Nicaragua; PA = Panama; PE = Peru; PY = Paraguay; SU = Suriname; UY = Uruguay; VZ = Venezuela.

On the other hand, Supplementary Figures 6 and 7 present the same interconnection developments for 2035 and 2045 under the scenario with national sovereignty constraints.

In this case, the overall regional transmission capacity also grows exponentially between 2025 and 2045. Starting from a relatively limited network in 2025, the region achieves stronger interconnections by 2035, with several corridors exceeding 15,000 MW — for example, Colombia–Panama (18,317 MW) and Venezuela–Colombia (19,429 MW). By 2045, these links reach even higher levels, including Venezuela–Colombia (30,307 MW) and Uruguay–Brazil (31,768 MW).

This evolution reflects a coordinated transition toward a resilient and strategically interconnected grid, capable of supporting cross-border renewable integration, enhancing energy security, and mitigating the geopolitical cost of sovereignty. Investment costs for 2035 are estimated at approximately 6.24 billion USD, rising by an additional 3 billion USD by 2045.

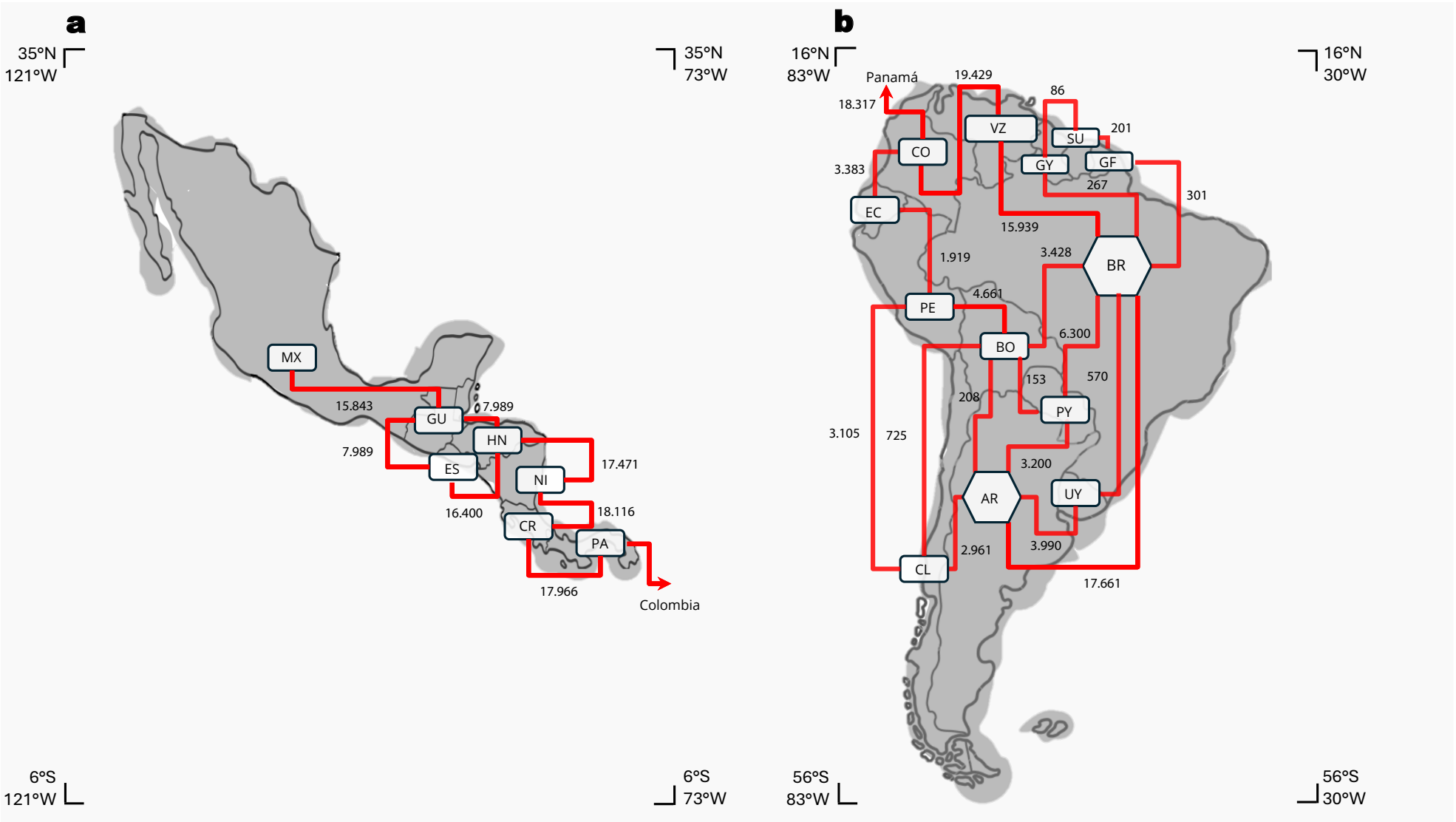

Supplementary Figure 6: Planned transmission interconnections and capacity expansions by 2035 (sovereignty scenario). **a** Mexico and Central America, covering the extent 35°N–6°S and 121°W–73°W. **b** South America, covering the extent 16°N–56°S and 83°W–30°W. In both panels, red lines denote cross-border interconnections and the adjacent numbers indicate the resulting total transfer capacity in MW (megawatts) after the modelled expansion under national sovereignty constraints, whereby each country must retain sufficient firm capacity to meet its own peak demand; line directions are nominal and all links are assumed bidirectional. Corner labels report geographic coordinates (latitude, longitude) referenced to the World Geodetic System 1984 datum (WGS 84, EPSG:4326); base maps are displayed in the geographic (Plate Carrée) projection and panels are not drawn to a common scale. Country codes: AR = Argentina; BO = Bolivia; BR = Brazil; CL = Chile; CO = Colombia; CR = Costa Rica; EC = Ecuador; ES = El Salvador; GF = French Guiana; GU = Guatemala; GY = Guyana; HN = Honduras; MX = Mexico; NI = Nicaragua; PA = Panama; PE = Peru; PY = Paraguay; SU = Suriname; UY = Uruguay; VZ = Venezuela.

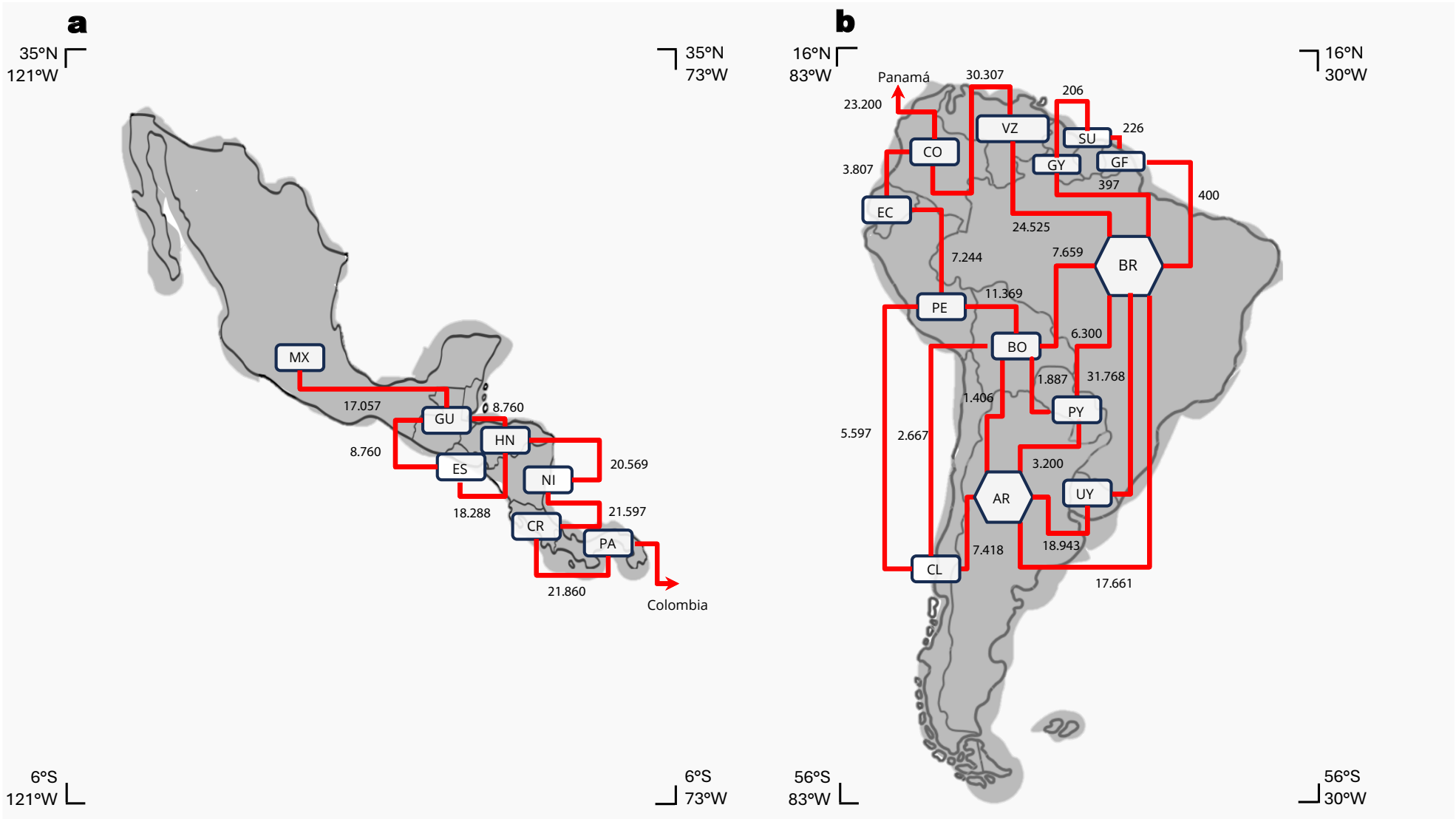

Supplementary Figure 7: Planned transmission interconnections and capacity expansions by 2045 (sovereignty scenario). **a** Mexico and Central America, covering the extent 35°N–6°S and 121°W–73°W. **b** South America, covering the extent 16°N–56°S and 83°W–30°W. In both panels, red lines denote cross-border interconnections and the adjacent numbers indicate the resulting total transfer capacity in MW (megawatts) after the modelled expansion under national sovereignty constraints; line directions are nominal and all links are assumed bidirectional. Corner labels report geographic coordinates (latitude, longitude) referenced to the World Geodetic System 1984 datum (WGS 84, EPSG:4326); base maps are displayed in the geographic (Plate Carrée) projection and panels are not drawn to a common scale. Country codes: AR = Argentina; BO = Bolivia; BR = Brazil; CL = Chile; CO = Colombia; CR = Costa Rica; EC = Ecuador; ES = El Salvador; GF = French Guiana; GU = Guatemala; GY = Guyana; HN = Honduras; MX = Mexico; NI = Nicaragua; PA = Panama; PE = Peru; PY = Paraguay; SU = Suriname; UY = Uruguay; VZ = Venezuela.

Supplementary Note 5: Quantitative Summary of Emissions and Costs

This appendix summarizes the main quantitative results of this study on regional electricity integration in Latin America. It presents, in particular, the emissions and cost results for scenarios with and without integration, together with a synthesis of the projected expansion of the interregional grid and the role of hydrological complementarity as the technical foundation of integration.

Absolute Carbon Emissions

Supplementary Table 6 summarizes the variation in CO_2 emissions under different hydrological availability scenarios for the Andean and Southern Cone regions, with and without investments in transmission infrastructure, for 2035 and 2045.

In 2035, the scenarios without integration show higher emission levels, reaching up to 97.3 $MtCO_2$ under low hydrological availability, whereas with transmission

Supplementary Table 6: Absolute carbon emissions ($MtCO_2$) for 2035 and 2045. $MtCO_2$ = million tonnes of carbon dioxide.

		Southern Cone Region					
		2035			2045		
Scenario	Andean Region	High	Medium	Low	High	Medium	Low
Without integration	High	85.6	84.6	93.4	174.4	172.9	183.5
Without integration	Medium	86.0	84.6	93.6	188.3	217.9	249.3
Without integration	Low	89.7	88.3	97.3	247.4	277.0	323.9
With integration	High	30.9	33.4	27.8	49.4	42.7	40.7
With integration	Medium	32.3	29.9	28.8	43.9	42.2	54.3
With integration	Low	28.0	28.1	27.1	47.2	66.0	98.9

investments emissions fall to as low as 27.1 $MtCO_2$ in the most demanding scenario. This reflects the positive impact of transmission infrastructure on emissions mitigation and on the more efficient use of available renewable resources.

In 2045, the trend persists, although absolute emissions increase due to projected growth in energy demand. Across the integrated cases, emissions range from approximately 41 to 99 $MtCO_2$, whereas the scenarios without integration reach as much as 324 $MtCO_2$, reinforcing the role of integration as an instrument for deep decarbonization.

Total Operation and Investment Costs

Supplementary Table 7 presents total system costs, including operation and investment in transmission, generation, and storage, for the years 2035 and 2045.

Supplementary Table 7: Summary of total operation and investment costs (USD billion) for 2035 and 2045. USD = U.S. dollars.

		Southern Cone Region					
		2035			2045		
Scenario	Andean Region	High	Medium	Low	High	Medium	Low
Without integration	High	67.7	70.4	74.3	125.8	128.5	132.7
Without integration	Medium	70.8	74.3	79.0	130.1	137.7	144.8
Without integration	Low	75.1	79.7	85.4	140.3	148.1	162.0
With integration	High	56.5	58.7	61.9	105.9	108.7	113.7
With integration	Medium	60.3	63.9	67.3	110.5	115.0	120.2
With integration	Low	65.4	69.7	73.9	118.7	124.0	130.1

Total costs are consistently higher in scenarios without energy transmission, reflecting the inefficiency of operating without exchange between regions. In 2045,

costs without integration range from approximately 126 to 162 billion USD, whereas with integration they decline to a range of approximately 106 to 130 billion USD, showing significant savings enabled by regional cooperation.

Hydrological availability has a crucial impact on these costs: scenarios with high availability show the lowest costs because of a larger hydropower contribution, whereas under low-hydrology conditions costs increase owing to greater reliance on thermal technologies and more expensive renewable options. Energy integration therefore becomes especially valuable under hydrological stress.

Taken together, these results complement the evidence presented in the previous sections on the projected expansion of the interregional grid and hydrological complementarity, reinforcing that both dimensions constitute the technical basis that explains the benefits of regional electricity integration observed in terms of emissions and costs.

Supplementary References

[1] Paredes, J. R. La red del futuro: desarrollo de una red eléctrica limpia y sostenible para américa latina (2017). URL https://doi.org/10.18235/0000937.

[2] International Energy Agency (IEA). World energy investment outlook. Tech. Rep., Organisation for Economic Co-operation and Development (OECD) (2024). URL https://www.iea.org/reports/world-energy-investment-outlook-2024.

[3] Organización Latinoamericana de Energía (OLADE). Panorama energético de américa latina y el caribe 2024. Informe técnico, OLADE (2024). URL https://www.olade.org/publicaciones/panorama-energetico-de-america-latina-y-el-caribe-2024/.

[4] U.S. Energy Information Administration (EIA). Annual energy outlook 2023. Tech. Rep., U.S. Department of Energy, Washington, D.C. (2023). URL https://www.eia.gov/outlooks/aeo/.

[5] Organización Latinoamericana de Energía (OLADE). Situación de la integración eléctrica en américa del sur. Tech. Rep., OLADE, Quito, Ecuador (2024).

[6] Organización Latinoamericana de Energía (OLADE), Tecnalia & Agencia Española de Cooperación Internacional para el Desarrollo (AECID). Vulnerabilidad y riesgo de los sistemas energéticos de américa latina y el caribe ante las amenazas del cambio climático: informe final del proyecto SCREEN ALC. Tech. Rep., OLADE, Quito, Ecuador (2023).
