# Supplementary material for: Climate urgency enables political alignment for Latin American power integration
Source: Nat Commun. 2026 Sep 17;17:10943. doi: 10.1038/s41467-026-77772-x (PMC13680428; doi:10.1038/s41467-026-77772-x)
Supplement: Supplementary file 2 [file 41467_2026_77772_MOESM2_ESM.pdf]

nature portfolio

Corresponding author(s): Luis M. Ramírez (luis.ramirez.c@ug.uchile.cl); Gerardo A. Blanco (gerardo.blanco@pucv.cl)

Last updated by author(s): May 30, 2026

Reporting Summary

Nature Portfolio wishes to improve the reproducibility of the work that we publish. This form provides structure for consistency and transparency in reporting. For further information on Nature Portfolio policies, see our Editorial Policies and the Editorial Policy Checklist.

Statistics

For all statistical analyses, confirm that the following items are present in the figure legend, table legend, main text, or Methods section.

n/a	Confirmed	
☒	☐	The exact sample size (*n*) for each experimental group/condition, given as a discrete number and unit of measurement
☒	☐	A statement on whether measurements were taken from distinct samples or whether the same sample was measured repeatedly
☒	☐	The statistical test(s) used AND whether they are one- or two-sided<br>*Only common tests should be described solely by name; describe more complex techniques in the Methods section.*
☒	☐	A description of all covariates tested
☐	☒	A description of any assumptions or corrections, such as tests of normality and adjustment for multiple comparisons
☒	☐	A full description of the statistical parameters including central tendency (e.g. means) or other basic estimates (e.g. regression coefficient) AND variation (e.g. standard deviation) or associated estimates of uncertainty (e.g. confidence intervals)
☒	☐	For null hypothesis testing, the test statistic (e.g. *F*, *t*, *r*) with confidence intervals, effect sizes, degrees of freedom and *P* value noted<br>*Give P values as exact values whenever suitable.*
☒	☐	For Bayesian analysis, information on the choice of priors and Markov chain Monte Carlo settings
☒	☐	For hierarchical and complex designs, identification of the appropriate level for tests and full reporting of outcomes
☐	☒	Estimates of effect sizes (e.g. Cohen's *d*, Pearson's *r*), indicating how they were calculated

Our web collection on statistics for biologists contains articles on many of the points above.

Software and code

Policy information about availability of computer code

Data collection	Quantitative input data were obtained from publicly available repositories: national energy balances and planning documents, Inter-American Development Bank (IDB) databases, Latin American Energy Organization (OLADE) reports, and International Energy Agency (IEA) databases. Data were compiled and pre-processed using custom Python scripts. No proprietary data collection software was used. Qualitative data for the MSA analysis were gathered through systematic bibliographic searches across academic databases (Web of Science, Scopus) and institutional repositories without automated text-mining software.
Data analysis	The energy system optimization model was implemented in Python (version 3.x) using the Pyomo optimization modeling language and solved with Gurobi (commercial solver). Post-processing, scenario analysis, and figure generation were performed using custom Python scripts (NumPy, Pandas, Matplotlib, GeoPandas). The interactive visualization platform (https://explorer.energia.la/) was developed using Python-based web tools; its source code is openly available at https://github.com/gerardoblancopy/LatamEnergyIntregrationExplorer. The qualitative MSA coding was performed manually using a predefined coding scheme based on Kingdon (2011). All model code, scenario generation scripts, post-processing routines, visualization scripts, and input files required to reproduce the main results and figures are publicly available in a permanent Zenodo repository at https://doi.org/10.5281/zenodo.19775192.

For manuscripts utilizing custom algorithms or software that are central to the research but not yet described in published literature, software must be made available to editors and reviewers. We strongly encourage code deposition in a community repository (e.g. GitHub). See the Nature Portfolio guidelines for submitting code & software for further information.

Data

Policy information about availability of data

All manuscripts must include a data availability statement. This statement should provide the following information, where applicable:

- Accession codes, unique identifiers, or web links for publicly available datasets
- A description of any restrictions on data availability
- For clinical datasets or third party data, please ensure that the statement adheres to our policy

The datasets used to construct the energy system model are derived from publicly available sources, including IDB, OLADE, and IEA reports [refs. 12, 19, 105]. Additional regional data on transmission infrastructure, renewable potential, and technology costs were obtained from official reports and academic literature [refs. 21, 23, 88, 104]. Model input data and processed model output data, which can be used to fully reproduce all analyses presented in this research, have been deposited in Zenodo [ref. 128: https://doi.org/10.5281/zenodo.19775192]. In addition, the complete set of simulation outputs (generation mixes, transmission flows, investments, and costs) is publicly accessible through an interactive visualization platform at https://explorer.energia.la/, whose source code is openly available at https://github.com/gerardoblancopy/LatamEnergyIntregrationExplorer. Source Data are provided with this paper.

Research involving human participants, their data, or biological material

Policy information about studies with human participants or human data. See also policy information about sex, gender (identity/presentation), and sexual orientation and race, ethnicity and racism.

Reporting on sex and gender	No. This study does not involve human participants, personal data, or biological material. The qualitative MSA analysis relies exclusively on publicly available secondary sources (peer-reviewed academic literature, institutional reports, and official policy declarations). No ethics approval was required. This is confirmed in the Declarations section of the manuscript ("Ethics approval and consent to participate: Not applicable. This study does not involve human participants or animal subjects").
Reporting on race, ethnicity, or other socially relevant groupings	Not applicable. This study does not involve human participants or human data.
Population characteristics	Not applicable. This study does not involve human participants or human data.
Recruitment	Not applicable. This study does not involve human participants or human data.
Ethics oversight	Not applicable. This study does not involve human participants or human data.

Note that full information on the approval of the study protocol must also be provided in the manuscript.

Field-specific reporting

Please select the one below that is the best fit for your research. If you are not sure, read the appropriate sections before making your selection.

☐ Life sciences ☒ Behavioural & social sciences ☐ Ecological, evolutionary & environmental sciences

For a reference copy of the document with all sections, see nature.com/documents/nr-reporting-summary-flat.pdf

Behavioural & social sciences study design

All studies must disclose on these points even when the disclosure is negative.

Study description	Mixed-methods study combining: (1) qualitative political analysis grounded in the Multiple Streams Approach (MSA), a theory of public policy change; and (2) quantitative long-term energy system expansion modeling. The qualitative component is a cross-sectional secondary data analysis, using deductive thematic coding of academic and policy literature to assess the convergence of problem, policy, and politics streams in Latin American power integration. The quantitative component is a deterministic, scenario-based, multi-period linear programming optimization covering 21 Latin American countries from 2025 to 2050 in five-year increments, with hourly resolution through representative days. The two components are integrated: the optimization model quantifies the economic costs of political dynamics (fragmentation, sovereignty preference, distrust) that the MSA framework identifies qualitatively, providing novel evidence-based leverage for policy entrepreneurs.
Research sample	Qualitative component: The analytical corpus comprises more than 100 peer-reviewed articles and institutional policy documents spanning approximately 70 years (1950s–2025) of Latin American energy integration history. Documents were drawn from academic databases (Web of Science, Scopus) and institutional repositories (OLADE, CEPAL/ECLAC, IDB, IEA, CIER). The sample is not a probabilistic draw from a defined population; it is a purposive, theoretically informed selection designed to capture the full range of problem, policy, and politics stream dynamics as conceptualized by MSA. The contemporary policy window analysis focuses on 2020–2025.<br><br>Quantitative component: The model covers all 21 Latin American countries included in South America, Central America, and Mexico; representing the vast majority of the region's electricity demand and renewable resource potential. Each country is represented as a

single node in the regional network. No sampling was applied; the model is comprehensive within its defined geographical scope. Input data include national-level hourly demand projections, technology cost and performance parameters, spatially explicit renewable energy potential profiles, CO_2 emission factors, fuel costs, and existing and planned cross-border transmission infrastructure.

Sampling strategy

Qualitative component: Purposive (judgmental) sampling guided by MSA theoretical constructs. Inclusion criteria: relevance to Latin American electricity integration, energy policy, climate change impacts on energy systems, and the MSA framework. The selection was iteratively refined to ensure coverage of key cases (SIEPAC, IIRSA, Itaipú/Yacyretá/Salto Grande, Argentina–Chile gas interconnections) and diverse political economy perspectives. No statistical power calculation was performed, as this is a qualitative theoretical framework application, not a survey or experiment.

Quantitative component: No statistical sampling. The scenario space was determined by analytical relevance: three integration levels × two institutional settings × nine hydrological configurations (3×3 matrix). Hydrological scenarios (High, Medium, Low inflow years) were based on historical ENSO cycle records for Andean and Southern Cone sub-regions, providing a plausible and policy-relevant range of climate variability rather than a statistically derived sample.

Data collection

Qualitative component: Systematic bibliographic review of peer-reviewed literature, technical reports from regional and international organizations (OLADE, CEPAL, IDB, IEA, CIER), and key policy declarations (e.g., 2023 Brasilia Consensus). Coding was performed manually by the authors using a predefined MSA coding scheme with the following categories: problem stream indicators, policy stream elements (proposed solutions and perceived viability), politics stream signals (national mood, political forces, government changes), political entrepreneurs, policy entrepreneurs, focusing events, policy windows, agenda windows, and decision windows. A timeline approach was used to organize events across streams and identify convergence patterns.

Quantitative component: All model inputs were derived from publicly available sources: national energy plans, IDB (Paredes, 2017), OLADE (Panorama Energético 2024; SCREEN-ALC 2023; Situación de la integración eléctrica en América del Sur 2024), and IEA (World Energy Investment Outlook 2024; Climate Impacts on Latin American Hydropower 2023). Transmission infrastructure data were obtained from official system operator publications and regional planning documents. The model was implemented in Python (Pyomo/Gurobi) and calibrated against 2022–2023 historical data from national energy balances and OLADE statistics.

Timing

Qualitative component: The historical MSA pattern analysis covers the 1950s through 2025. The contemporary policy window analysis is primarily based on material from 2020–2025, chosen to correspond with the availability of detailed data and recent policy shifts. No primary field data collection was conducted.

Quantitative component: Optimization horizon: 2025–2050 in five-year increments. Within each period, hourly resolution is captured through representative days. Model calibration uses 2022–2023 historical data. Results are analyzed primarily at 2035 and 2045 milestones, as reported in Figs. 1–6 and Supplementary Tables 5–6.

Data exclusions

No data were excluded from the analyses.

Qualitative component: The literature review was designed to be inclusive, deliberately encompassing diverse political economy perspectives — including critical views on integration obstacles — to avoid confirmation bias. No documents were excluded post-hoc based on results direction.

Quantitative component: All 21 modeled countries are included in every scenario. No countries were excluded after initial model scope definition. Guyana, Suriname, and French Guiana are included as candidate connection nodes despite limited existing data, with parameters estimated from regional benchmarks; this is noted as a model limitation.

Non-participation

Not applicable. This study involves no human participants. There were no dropouts or refusals, as all data derive from secondary publicly available sources.

Randomization

Not applicable. The study does not involve allocation of participants or samples into experimental groups. In the quantitative component, the assignment of countries and scenarios to analytical conditions is deterministic and theory-driven: integration blocs (Andean Community, Southern Cone, SIEPAC) reflect existing political alliances and geographical realities, not random assignment. In the qualitative component, the MSA framework provides the analytical structure for interpreting historical and contemporary evidence without randomization.

Reporting for specific materials, systems and methods

We require information from authors about some types of materials, experimental systems and methods used in many studies. Here, indicate whether each material, system or method listed is relevant to your study. If you are not sure if a list item applies to your research, read the appropriate section before selecting a response.

Materials & experimental systems

n/a	Involved in the study
☒	☐ Antibodies
☒	☐ Eukaryotic cell lines
☒	☐ Palaeontology and archaeology
☒	☐ Animals and other organisms
☒	☐ Clinical data
☒	☐ Dual use research of concern
☒	☐ Plants

Methods

n/a	Involved in the study
☒	☐ ChIP-seq
☒	☐ Flow cytometry
☒	☐ MRI-based neuroimaging

Plants

Seed stocks

This study does not involve plant material of any kind. No seed stocks or other plant material were used.

Novel plant genotypes

This study does not involve plants, transgenic approaches, gene editing, chemical or radiation-based mutagenesis, or hybridization. No novel plant genotypes were produced or used.

Authentication

No plant material, seed stocks, or plant genotypes were used in this study, and therefore no authentication procedures were required or performed.
