# Supplementary material for: Climate urgency enables political alignment for Latin American power integration
Source: Nat Commun. 2026 Sep 17;17:10943. doi: 10.1038/s41467-026-77772-x (PMC13680428; doi:10.1038/s41467-026-77772-x)
Supplement: Supplementary file 3 [file 41467_2026_77772_MOESM3_ESM.pdf]

nature portfolio

Peer Review File

Climate urgency enables political alignment for Latin American power integration

Corresponding Author: Dr Luis Ramírez

This file contains all reviewer reports in order by version, followed by all author rebuttals in order by version.

Version 0:

Reviewer comments:

Reviewer #1

(Remarks to the Author)
What are the noteworthy results?

This is a very good paper with highly relevant policy oriented results to fostering electricity integration in Latin America by quantifying the costs of fragmentation. I enjoyed reading it and honestly think it deserves publication, with only very minor revisions to better support its geopolitical content with related references and perhaps addressing some minor points highlighted at the end of this review

Will the work be of significance to the field and related fields?

Yes, highly significant

How does it compare to the established literature?

It offers a novel approach by applying the MSA framework. It goes beyond traditional techno economic analysis by including political and, especially, geopolitical factors. These geopolitical aspects are commonly found to be among the main obstacles to Latin American energy and electricity integration.

Does the work support the conclusions and claims?

Yes, but I miss more elaboration on geopolitical drivers. For instance in p.3 the paper refers to Cold War geopolitics as exogenous factors, which is right. P. 9 refers to geopolitical cost but lacks literature references. Along this line, I miss some references to current geopolitical drivers contributing to electricity integration, like the recent Trump's corollary to the Monroe doctrine included in the recent US National Security Strategy, which can foster closer integration within Latin America. Also, new agreements like EU-Mercosur can act as an exogenous driver favoring integration with new financing and regulatory learning. New energy policy divergences in recently elected governments (Argentina, Chile, Bolivia, Ecuador) eventually leading to more nationalistic approaches could also be mentioned.

Regarding geopolitics, the paper only refers to two papers dealing with LA energy geopolitics (50 and 71) while there are several recent papers dealing with LA energy geopolitics. Here are some of them that could further support the paper's arguments:

Escribano et al. (2023). Renewable energies in Latin America: Resources, public policies, and geopolitics. In Handbook on the geopolitics of the energy transition (pp. 535-549). Edward Elgar Publishing.

Guerrero, A. L. (2021). Geopolitics of global energy transformation and territorial dynamics of energy transition in South America. Ambiente & Sociedade, 24, e00263.

Reyes, C. V. (2017). Energy Integration in South America and Global Geopolitics. In Regionalism, Development and the Post-Commodities Boom in South America (pp. 147-164). Cham: Springer International Publishing.

Some references dealing with the international political economy of energy integration may also be useful:
Sabbatella, I., & Santos, T. (2020). The IPE of regional energy integration in South America. In The Routledge Handbook to Global Political Economy (pp. 719-740). Routledge.

For the exogenous factor of the EU and the EU-Mercosur , EU-Chile and EU-Mexico agreements,a ongoing others, see for instance:

Averchenkova et al. (2025). Beyond leading by example: enhanced EU-LAC climate cooperation—the case of Brazil, Chile and Mexico. International Environmental Agreements: Politics, Law and Economics, 1-18.

Are there any flaws in the data analysis, interpretation and conclusions?

No, but more references to geopolitics would help to support the paper's arguments

Do these prohibit publication or require revision?

Minor revision to include the literature on the geopolitics of energy integration in the region

Is the methodology sound?

Yes, but this reviewer is no expert on quantitative methods, but rather on energy policy and energy geopolitics

Does the work meet the expected standards in your field?

Yes

Is there enough detail provided in the methods for the work to be reproduced?

Yes

Finally, I have some minor formal comments: please revise the use of brackets because most of the initial brackets seem to have been changed in format to closing ones (eg. ”policy primeval soup”, but there are many others). Also, in p. 2 there are two redundant sentences, and the second seems to be incomplete: "Under this context, regional integration—complementing solar in northern Chile, wind in Patagonia, and flexible hydro in the Amazon—offers a technical solution to buffer variability and bolster cross-borders resilience9;18;19.
Regional power integration, complementing northern solar, Patagonian wind, and flexible hydro, can enhance resilience and reduce costs9;18;20–23" Both says the same, and the second seems to lack Chile after northern.

I also have a doubt in p.5, where it is said : "the Andean region (e.g., Colombia, Peru) might experience stabiliza-
tion or marginal gains due to altered precipitation93–95. This differential exposure
significantly exacerbates risks for highly hydro-dependent countries like Paraguay and
Ecuador " Ecuador is also an Andean country, if it is differently affected than Colombia and Peru it would be good to specify it and why.

Another point I would like to raise regards OLADE proposals. In my humble view, one of the important drivers for regional integration that is explicitly recognized by the paper is cost reduction, where OLADE as proposed the concept of powershoring as a comparative advantage for LA, perhaps this would support the paper's cost argument by adding the international competitiveness dimension.

In p. 13 there are references to SIEAL and a "new" LA Energy Treaty. I agree, but perhaps would be wise to recognize that many analyst remain quite skeptical on the political and geopolitical obstacles to it, as many of the suggested geopolitical references above show. Also, because it was non-existent, I don't understand very well why this is a "new" pact.

Finally, the different integration pathways are only introduced in p.15 and developed in p. 17 in the methods section. I would suggest to introduce such pathways form the start of the paper, even in the introduction, for the reader to understand what options the authors are considering.

(Remarks on code availability)

Reviewer #3

(Remarks to the Author)
Dear authors,

Firstly, I'd like to say that your work is scientific consistent, and, at the same time, very relevant in terms of improving the socioeconomic development of Latin America countries. So, Congratulations!!!

But, of course, I have my comments looking for possible improvements.

The central argument of your study, as I understand it, is that the necessary energy integration in Latin America can be accelerated by greater political cohesion and by the urgency of climate emergency, which is indeed a coherent proposition. However, this outcome depends on profound transformations in the institutional, economic, and geopolitical arrangements of the region. I recognize that the methodology you applied, centered on the Multiple Streams Approach (MSA), largely captures the complexity inherent in such an argument. Nevertheless, I believe that some of the premises you adopted would require, so to speak, a lower degree of simplification, particularly with regard to geopolitical aspects, which often depend more on the decisions and interests of other countries—for example, in securing access to the metals required for the energy transition, as well as a broader range of commodities—especially those countries representing the Global North and China. I believe that you should improve this analysis, considering, for example, the theory of accumulation by dispossession, developed by David Harvey, describes how neoliberal capitalism perpetuates primitive accumulation through the forced appropriation of public and common assets, transferring wealth to elites. It involves privatization, financialization, crisis management, and state redistribution, resulting in the loss of collective rights and assets. This aspect, however, does not imply a failure of the study, nor does it prevent its publication in the prestigious Nature Communications journal.
Furthermore, I believe that the methodology employed (“mixed-methods research design, combining qualitative political analysis grounded in the Multiple Streams Approach with quantitative, long-term energy system expansion modeling”) represents the study's main strength. It is a solid methodological approach that ultimately enables the work to achieve a high standard of scientific quality, particularly in the field of energy planning. In this context, the study demonstrates inherent reproducibility, and the quality of the English used appears to be excellent.
In page 2, it’s not “As one of the leading sources of greenhouse gas emissions, the energy sector…”, in fact, globally, the energy sector is the leading source of GHF emissions, responding for more than 70%. Please correct.
I believe that the development of the study itself supports the analyses and conclusions presented. However, I suggest that a section entitled "Conclusion" should be included, possibly by drawing up material from the excellent section "Quantitative Analysis: Regional Power System Expansion Model," as well as from other analytical sections. In this sense, I understand that it could also be better emphasized in the conclusions that greater political cohesion can create the necessary environment for the climate emergency to translate into effective action, but it will be necessary to overcome differences in interests and strengthen lasting cooperative mechanisms (or something along those lines).

Under these considerations, I believe that the manuscript "Climate urgency enables political alignment for Latin American power integration" possesses sufficient quality and scientific robustness to be published in the prestigious Nature Communications journal. However, the recommendations I have outlined should first be worked on by the authors – I emphasize the obvious: this work is of excellent quality and does not require any structural improvement.

Kind regards,

Reviewer.
February 23, 2026.

(Remarks on code availability)

Version 1:

Reviewer comments:

Reviewer #1

(Remarks to the Author)
All my comments and suggestions were adequately addressed

(Remarks on code availability)
All my comments and suggestions were adequately addressed

Reviewer #3

(Remarks to the Author)
Dear Author,

With due care, and using the Code Ocean Private Link, I analyzed and reviewed your review based on my comments below:

"The central argument of your study, as I understand it, is that the necessary energy integration in Latin America can be accelerated by greater political cohesion and by the urgency of climate emergency, which is indeed a coherent proposition. However, this outcome depends on profound transformations in the institutional, economic, and geopolitical arrangements of the region. I recognize that the methodology you applied, centered on the Multiple Streams Approach (MSA), largely captures the complexity inherent in such an argument. Nevertheless, I believe that some of the premises you adopted would require, so to speak, a lower degree of simplification, particularly with regard to geopolitical aspects, which often depend

more on the decisions and interests of other countries—for example, in securing access to the metals required for the energy transition, as well as a broader range of commodities—especially those countries representing the Global North and China. I believe that you should improve this analysis, considering, for example, the theory of accumulation by dispossession, developed by David Harvey, describes how neoliberal capitalism perpetuates primitive accumulation through the forced appropriation of public and common assets, transferring wealth to elites. It involves privatization, financialization, crisis management, and state redistribution, resulting in the loss of collective rights and assets. This aspect, however, does not imply a failure of the study, nor does it prevent its publication in the prestigious Nature Communications journal.
Furthermore, I believe that the methodology employed ("mixed-methods research design, combining qualitative political analysis grounded in the Multiple Streams Approach with quantitative, long-term energy system expansion modeling") represents the study's main strength. It is a solid methodological approach that ultimately enables the work to achieve a high standard of scientific quality, particularly in the field of energy planning. In this context, the study demonstrates inherent reproducibility, and the quality of the English used appears to be excellent.
In page 2, it's not "As one of the leading sources of greenhouse gas emissions, the energy sector…", in fact, globally, the energy sector is the leading source of GHF emissions, responding for more than 70%. Please correct.
I believe that the development of the study itself supports the analyses and conclusions presented. However, I suggest that a section entitled "Conclusion" should be included, possibly by drawing up material from the excellent section "Quantitative Analysis: Regional Power System Expansion Model," as well as from other analytical sections. In this sense, I understand that it could also be better emphasized in the conclusions that greater political cohesion can create the necessary environment for the climate emergency to translate into effective action, but it will be necessary to overcome differences in interests and strengthen lasting cooperative mechanisms (or something along those lines)."

With due care, and using the Code Ocean Private Link, I analyzed and reviewed your review based on my comments below:

I believe you have carefully reviewed my comments, and I believe you have indeed made an effort to implement, in one way or another, all of my recommendations; in this context, I approve the publication of the work in the prestigious journal Nature Communications.

Congratulations, dear Author!!!

Reviewer

June 7, 2026.

(Remarks on code availability)
Dear Author,

With due care, and using the Code Ocean Private Link, I analyzed and reviewed your review based on my comments below:

"The central argument of your study, as I understand it, is that the necessary energy integration in Latin America can be accelerated by greater political cohesion and by the urgency of climate emergency, which is indeed a coherent proposition. However, this outcome depends on profound transformations in the institutional, economic, and geopolitical arrangements of the region. I recognize that the methodology you applied, centered on the Multiple Streams Approach (MSA), largely captures the complexity inherent in such an argument. Nevertheless, I believe that some of the premises you adopted would require, so to speak, a lower degree of simplification, particularly with regard to geopolitical aspects, which often depend
more on the decisions and interests of other countries—for example, in securing access to the metals required for the energy transition, as well as a broader range of commodities—especially those countries representing the Global North and China. I believe that you should improve this analysis, considering, for example, the theory of accumulation by dispossession, developed by David Harvey, describes how neoliberal capitalism perpetuates primitive accumulation through the forced appropriation of public and common assets, transferring wealth to elites. It involves privatization, financialization, crisis management, and state redistribution, resulting in the loss of collective rights and assets. This aspect, however, does not imply a failure of the study, nor does it prevent its publication in the prestigious Nature Communications journal.
Furthermore, I believe that the methodology employed ("mixed-methods research design, combining qualitative political analysis grounded in the Multiple Streams Approach with quantitative, long-term energy system expansion modeling") represents the study's main strength. It is a solid methodological approach that ultimately enables the work to achieve a high standard of scientific quality, particularly in the field of energy planning. In this context, the study demonstrates inherent reproducibility, and the quality of the English used appears to be excellent.
In page 2, it's not "As one of the leading sources of greenhouse gas emissions, the energy sector…", in fact, globally, the energy sector is the leading source of GHF emissions, responding for more than 70%. Please correct.
I believe that the development of the study itself supports the analyses and conclusions presented. However, I suggest that a section entitled "Conclusion" should be included, possibly by drawing up material from the excellent section "Quantitative Analysis: Regional Power System Expansion Model," as well as from other analytical sections. In this sense, I understand that it could also be better emphasized in the conclusions that greater political cohesion can create the necessary environment for the climate emergency to translate into effective action, but it will be necessary to overcome differences in interests and strengthen lasting cooperative mechanisms (or something along those lines)."

With due care, and using the Code Ocean Private Link, I analyzed and reviewed your review based on my comments below:

I believe you have carefully reviewed my comments, and I believe you have indeed made an effort to implement, in one way or another, all of my recommendations; in this context, I approve the publication of the work in the prestigious journal Nature Communications.

Congratulations, dear Author!!!

Reviewer

June 7, 2026.

Open Access This Peer Review File is licensed under a Creative Commons Attribution 4.0 International License, which permits use, sharing, adaptation, distribution and reproduction in any medium or format, as long as you give appropriate credit to the original author(s) and the source, provide a link to the Creative Commons license, and indicate if changes were made.
In cases where reviewers are anonymous, credit should be given to 'Anonymous Referee' and the source.
The images or other third party material in this Peer Review File are included in the article's Creative Commons license, unless indicated otherwise in a credit line to the material. If material is not included in the article's Creative Commons license and your intended use is not permitted by statutory regulation or exceeds the permitted use, you will need to obtain permission directly from the copyright holder.
To view a copy of this license, visit https://creativecommons.org/licenses/by/4.0/

Reviewer #1 Comments

General assessment: "This is a very good paper with highly relevant policy oriented results to fostering electricity integration in Latin America by quantifying the costs of fragmentation. I enjoyed reading it and honestly think it deserves publication, with only very minor revisions to better support its geopolitical content with related references and perhaps addressing some minor points highlighted at the end of this review. It offers a novel approach by applying the MSA framework. It goes beyond traditional techno-economic analysis by including political and, especially, geopolitical factors. These geopolitical aspects are commonly found to be among the main obstacles to Latin American energy and electricity integration."

Comment R1.1 - Inadequate discussion of geopolitical factors; missing literature references (p.3 and p.9)

Reviewer Comment: *Does the work support the conclusions and claims? Yes, but I miss more elaboration on geopolitical drivers. For instance in p.3 the paper refers to Cold War geopolitics as exogenous factors, which is right. P.9 refers to geopolitical cost but lacks literature references. Along this line, I miss some references to current geopolitical drivers contributing to electricity integration, like the recent Trump's corollary to the Monroe doctrine included in the recent US National Security Strategy, which can foster closer integration within Latin America. Also, new agreements like EU-Mercosur can act as an exogenous driver favoring integration with new financing and regulatory learning. New energy policy divergences in recently elected governments (Argentina, Chile, Bolivia, Ecuador) eventually leading to more nationalistic approaches could also be mentioned.*

Regarding geopolitics, the paper only refers to two papers dealing with LA energy geopolitics (50 and 71) while there are several recent papers dealing with LA energy geopolitics. Here are some of them that could further support the paper's arguments:

Escribano et al. (2023). Renewable energies in Latin America: Resources, public policies, and geopolitics. In Handbook on the geopolitics of the energy transition (pp. 535-549). Edward Elgar Publishing.

Guerrero, A. L. (2021). Geopolitics of global energy transformation and territorial dynamics of energy transition in South America. Ambiente & Sociedade, 24, e00263.

Reyes, C. V. (2017). Energy Integration in South America and Global Geopolitics. In Regionalism, Development and the Post-Commodities Boom in South America (pp. 147-164). Cham: Springer International Publishing.

Some references dealing with the international political economy of energy integration may also be useful:

Sabbatella, I., & Santos, T. (2020). The IPE of regional energy integration in South America. In The Routledge Handbook to Global Political Economy (pp. 719-740). Routledge.

For the exogenous factor of the EU and the EU-Mercosur, EU-Chile and EU-Mexico agreements, and ongoing others, see for instance:

Averchenkova et al. (2025). Beyond leading by example: enhanced EU-LAC climate cooperation-the case of Brazil, Chile and Mexico. International Environmental Agreements: Politics, Law and Economics, 1-18.

Are there any flaws in the data analysis, interpretation and conclusions? No, but more references to geopolitics would help to support the paper's arguments.

Do these prohibit publication or require revision? Minor revision to include the literature on the geopolitics of energy integration in the region.

Is the methodology sound? Yes, but this reviewer is no expert on quantitative methods, but rather on energy policy and energy geopolitics.

Does the work meet the expected standards in your field? Yes.

Is there enough detail provided in the methods for the work to be reproduced? Yes.

AR: *We sincerely thank Reviewer #1 for this insightful and detailed comment. We have substantially expanded the geopolitical analysis to address all points raised: (i) We have added a new paragraph in the Politics Stream section that explicitly discusses contemporary geopolitical forces shaping the integration landscape—both endogenous and exogenous. This includes discussion of the Monroe Doctrine corollary in the recent US National Security Strategy (White House, 2025), EU-LAC climate cooperation and agreements (EU-Mercosur, EU-Chile), and the risk of resource nationalism driven by recent electoral shifts in Argentina, Chile, Bolivia, and Ecuador. (ii) We have incorporated all suggested references: Escribano et al. (2023), Guerrero (2021), Reyes (2017), Sabbatella & Santos (2020), and Averchenkova et al. (2025). Additionally, the IIRSA paragraph now cites Reyes (2017) to strengthen the geopolitical framing of that case. (iii) The three integration pathways (Full Integration, Partial Integration, and Fragmented Systems) have been introduced early in the Introduction section, as requested in Comment R1.8. These changes are highlighted in the revised manuscript.*

Original	Revised
This period exemplifies a classic MSA configuration, where convergence of streams was largely driven by exogenous forces. The problem stream (oil crisis) and the politics stream (Cold War geopolitics) were shaped by global dynamics. Coupled with binational hydroelectric policies and enabled by policy entrepreneurs, this opened a policy window that led to landmark bilateral projects such as Itaipu, Salto Grande, and Yacyreta. [...] although the climate crisis is exogenous in origin, it generates deeply shared, endogenous vulnerabilities across Latin America such as water stress, energy insecurity, and economic disruption. These conditions more closely resemble the SIEPAC context than the fragmented geopolitics of the 1970s, suggesting that the current policy window may favour multilateral convergence.	*This period exemplifies a classic MSA configuration, where convergence of streams was largely driven by exogenous forces. The problem stream (oil crisis) and the politics stream (Cold War geopolitics) were shaped by global dynamics. Coupled with binational hydroelectric policies and enabled by policy entrepreneurs, this opened a policy window that led to landmark bilateral projects such as Itaipu, Salto Grande, and Yacyreta [79-81]. Similar cyclical patterns of cooperation and stagnation have characterized other integration efforts, such as the Argentina-Chile gas interconnections [82]. [...] although the climate crisis is exogenous in origin, it generates deeply shared, endogenous vulnerabilities across Latin America—such as water stress, energy insecurity, and economic disruption. These conditions more closely resemble the SIEPAC context than the fragmented geopolitics of the 1970s, suggesting that the current policy window may favor multilateral convergence—if political will aligns accordingly [20,61,62].*<br><br>***The Politics Stream: Overcoming Fragmentation by Quantifying its Costs***<br>*[NEW PASSAGE ADDED TO POLITICS STREAM SECTION]*<br><br>*This political landscape is further shaped by contemporary geopolitical forces—both endogenous and exogenous—that simultaneously create new pressures and opportunities for regional cooperation [87][108-110]. On the one hand, the global geopolitics of the energy transition*

	has increased Latin America's strategic relevance, given the region's renewable resource base and its role in critical mineral supply chains for low-carbon technologies, including lithium, copper, nickel, graphite, and rare earth elements [108,109,111,112]. Similarly, EU-LAC climate cooperation and agreements such as EU-Mercosur and EU-Chile provide exogenous momentum by introducing opportunities for financing, regulatory learning, and institutional convergence [113-116]. Concurrently, the renewed projection of US strategic primacy over the Western Hemisphere—articulated in the recent US National Security Strategy as a contemporary corollary to the Monroe doctrine—constitutes a further exogenous pressure that, while heightening external uncertainty, may paradoxically reinforce intra-Latin American cooperation as a defensive strategy [108,109,117]. On the other hand, electoral shifts and energy-policy divergences in countries such as Argentina, Chile, Bolivia, and Ecuador risk reinvigorating resource nationalism and sovereignty-first approaches, potentially counteracting integration efforts [108-110].

Comment R1.2 - 'Geopolitical cost' section lacks literature references (p.9)

Reviewer Comment: *P.9 refers to geopolitical cost but lacks literature references. Along this line, I miss some references to current geopolitical drivers contributing to electricity integration. The references suggested in Comment R1.1 above (Escribano et al., 2023; Guerrero, 2021; Reyes, 2017; Sabbatella & Santos, 2020) should be considered to support the geopolitical cost argument.*

AR: *The "Geopolitical Cost" paragraph in the Politics Stream section now explicitly references the suggested geopolitical literature (Escribano et al., 2023; Guerrero, 2021; Reyes, 2017; Sabbatella & Santos, 2020) to support the geopolitical cost argument. The revised paragraph contextualizes the quantified sovereignty cost within the broader literature on Latin American energy geopolitics, noting that sovereignty concerns and historical distrust are consistently identified as structural barriers to cooperation. These changes are highlighted in the revised manuscript.*

Original	Revised
The 'Geopolitical Cost' of Sovereignty Preference: We estimate the economic penalty incurred when national sovereignty constraints hinder optimal regional cooperation (see Methods; Fig. 4). This cost reveals a significant inefficiency, amounting to several billion USD annually ($4.6-7.9 billion by 2045 with integration), driven by suboptimal system deployment when national priorities are prioritized over regional synergy. Detailed dynamics are presented in Fig. 4.	*The 'Geopolitical Cost' of Sovereignty Preference: We estimate the economic penalty incurred when national sovereignty constraints hinder optimal regional cooperation (see Methods; Fig. 4). This cost reveals a significant inefficiency, amounting to several billion USD annually ($4.6–7.9 billion by 2045 with integration), driven by suboptimal system deployment when national priorities are prioritized over regional synergy. This framing draws on recent literature on the geopolitics of Latin American energy integration [108-110], which consistently identifies sovereignty concerns and historical distrust as structural barriers to*

	cooperation. Detailed dynamics are presented in Fig. 4.

Comment R1.3 - International competitiveness dimension / OLADE 'powershoring' concept

Reviewer Comment: *Another point I would like to raise regards OLADE proposals. In my humble view, one of the important drivers for regional integration that is explicitly recognized by the paper is cost reduction, where OLADE has proposed the concept of 'powershoring' as a comparative advantage for LA. Perhaps this would support the paper's cost argument by adding the international competitiveness dimension.*

AR: *We thank Reviewer #1 for this valuable suggestion. A new paragraph has been added to the Policy Stream section explicitly incorporating OLADE's concept of powershoring—Latin America's abundant and low-cost renewable energy as a structural comparative advantage for attracting energy-intensive industries and green hydrogen value chains—citing OLADE's 2024 report on electrical integration in South America [107]. The paragraph explains that regional-scale integration would amplify this advantage by enabling more stable and affordable cross-border electricity supply, reinforcing the region's positioning in global decarbonized supply chains. This international competitiveness dimension is framed as a further economic argument for integration, complementing the cost-reduction and resilience arguments already present. The change is highlighted in the revised manuscript.*

**Original**	**Revised**
Lower System Costs: Integration yields compelling economic advantages by enabling efficient resource allocation. By 2045, total system costs in integrated scenarios range from $106-130 billion USD annually, compared to $126-162 billion USD for isolated systems (Table 2). This translates into potential net economic savings (avoided losses) reaching up to $32 billion USD annually by that year, particularly significant during periods of water scarcity. [...] Further evidenced by recent calls to action and strategic analyses from key regional institutions like OLADE, which highlight both the urgency and the renewed potential for advancing electrical integration.	*Lower System Costs: Integration yields compelling economic advantages by enabling efficient resource allocation. By 2045, total system costs in integrated scenarios range from $106–130 billion USD annually, compared to $126–162 billion USD for isolated systems (Supplementary Table 6). This translates into potential net economic savings (avoided losses) reaching up to $32 billion USD annually by that year, particularly significant during periods of water scarcity (Fig. 2). [...] Beyond energy security and emissions reductions, regional integration also carries significant implications for international economic competitiveness. OLADE has advanced the concept of powershoring—whereby Latin America's abundant and low-cost renewable energy potential becomes a structural comparative advantage for attracting energy-intensive industries and green hydrogen value chains—as part of its analysis of regional electricity integration [107]. Integrating power systems at the regional scale would amplify this advantage by enabling more stable, affordable, and reliable electricity supply across borders, reinforcing the region's positioning in global decarbonized supply chains. This international competitiveness dimension adds a further layer to the cost argument for integration: beyond avoiding internal system inefficiencies, fragmentation also forfeits the opportunity to leverage collective renewable*

	endowments as a geopolitical and economic asset in the global energy transition.

Comment R1.4 - Two redundant sentences on p.2 (minor)

Reviewer Comment: *In p.2 there are two redundant sentences, and the second seems to be incomplete: 'Under this context, regional integration-complementing solar in northern Chile, wind in Patagonia, and flexible hydro in the Amazon-offers a technical solution to buffer variability and bolster cross-borders resilience; Regional power integration, complementing northern solar, Patagonian wind, and flexible hydro, can enhance resilience and reduce costs.' Both say the same, and the second seems to lack Chile after northern.*

AR: *The second sentence ("Regional power integration, complementing northern solar, Patagonian wind, and flexible hydro, can enhance resilience and reduce costs") has been removed. The first sentence has been slightly expanded to incorporate the cost-reduction element: "...offers a technical solution to buffer variability, bolster cross-border resilience, and reduce system costs." The word "cross-borders" has also been corrected to "cross-border." The change is highlighted in the revised manuscript.*

Original	Revised
Under this context, regional integration-complementing solar in northern Chile, wind in Patagonia, and flexible hydro in the Amazon-offers a technical solution to buffer variability and bolster cross-borders resilience. Regional power integration, complementing northern solar, Patagonian wind, and flexible hydro, can enhance resilience and reduce costs. [Note: both sentences convey the same idea; the second omits 'Chile' after 'northern']	*Under this context, regional integration—complementing solar in northern Chile, wind in Patagonia, and flexible hydro in the Amazon—offers a technical solution to buffer variability, bolster cross-border resilience, and reduce system costs [9,18-23]. [Note: the redundant second sentence has been removed. The revised sentence now integrates 'reduce costs' directly into the first sentence and uses 'cross-border' (not 'cross-borders') for consistency.]*

Comment R1.5 - Bracket format issue throughout the text (minor)

Reviewer Comment: *Please revise the use of brackets because most of the initial brackets seem to have been changed in format to closing ones (e.g. 'policy primeval soup', but there are many others).*

AR: *A careful review of the entire manuscript has been conducted to correct all instances of inconsistent quotation mark formatting. The specific example cited ("policy primeval soup") and all other affected passages throughout the text have been corrected so that opening and closing quotation marks appear in the standard typographic form. The corrections are reflected throughout the revised manuscript.*

Original	Revised
Solutions to societal problems often germinate within specialist communities-Kingdon's "policy primeval soup"-long before capturing widespread political attention.<br><br>*[Note: reviewer flags that throughout the manuscript, most opening brackets appear to have*	*Solutions to societal problems often germinate within specialist communities—Kingdon's "policy primeval soup"—long before capturing widespread political attention.*<br><br>*[Note: throughout the revised manuscript, the bracket/quotation formatting has been corrected so that opening and closing quotation marks*

been changed in format to closing ones. This affects multiple instances across the text.]	*appear in the standard typographic form. The specific example flagged by the reviewer ("policy primeval soup") and all other affected instances have been corrected.]*

Comment R1.6 - Ecuador classification as Andean country (p.5)

Reviewer Comment: *I also have a doubt in p.5, where it is said: 'the Andean region (e.g., Colombia, Peru) might experience stabilization or marginal gains due to altered precipitation. This differential exposure significantly exacerbates risks for highly hydro-dependent countries like Paraguay and Ecuador.' Ecuador is also an Andean country; if it is differently affected than Colombia and Peru it would be good to specify it and why.*

AR: *The reviewer is correct that Ecuador is also an Andean country, and the original text was insufficiently precise. We have added a clarifying sentence explaining Ecuador's exceptional situation within the Andean context: "Ecuador, despite its Andean location, constitutes an important exception: its hydroelectric system is concentrated in basins particularly sensitive to ENSO-driven variability and projected precipitation reductions in its coastal and inter-Andean watersheds, making it more closely aligned with the vulnerability profile of Southern Cone nations [11,15]." This addition clarifies the geographic and climatic basis for Ecuador's different exposure profile compared to Colombia and Peru. The change is highlighted in the revised manuscript.*

**Original**	**Revised**
Modeling studies project that Latin America's hydroelectric potential could diminish by 7.5% to 17.4% by the end of the century, contingent on global emissions pathways. These impacts are regionally heterogeneous: consistent declines are anticipated in Central America, Mexico, and Southern Cone nations (e.g., Argentina, Chile), whereas the Andean region (e.g., Colombia, Peru) might experience stabilization or marginal gains due to altered precipitation. This differential exposure significantly exacerbates risks for highly hydro-dependent countries like Paraguay and Ecuador and starkly underscores how escalating global emissions intensify regional energy vulnerability.	*Modeling studies project that Latin America's hydroelectric potential could diminish by 7.5% to 17.4% by the end of the century, contingent on global emissions pathways [2,11,92,93]. These impacts are regionally heterogeneous: consistent declines are anticipated in Central America, Mexico, and Southern Cone nations (e.g., Argentina, Chile), whereas parts of the Andean region (e.g., Colombia, Peru) might experience stabilization or marginal gains due to altered precipitation patterns [94-96]. Ecuador, despite its Andean location, constitutes an important exception: its hydroelectric system is concentrated in basins particularly sensitive to ENSO-driven variability and projected precipitation reductions in its coastal and inter-Andean watersheds, making it more closely aligned with the vulnerability profile of Southern Cone nations [11,15]. This differential exposure significantly exacerbates risks for highly hydro-dependent countries like Paraguay and Ecuador, and starkly underscores how escalating global emissions intensify regional energy vulnerability in ways that do not follow simple geographic boundaries [12,15,97].*

Comment R1.7 - SIEAL and 'new' Latin American Energy Treaty skepticism (p.13)

Reviewer Comment: *In p.13 there are references to SIEAL and a 'new' LA Energy Treaty. I agree, but perhaps it would be wise to recognize that many analysts remain quite skeptical on the political and geopolitical obstacles to it, as many of the suggested geopolitical references above show. Also, because it was non-existent, I don't understand very well why this is a 'new' pact.*

AR: *We have made two clarifications in response: (i) Regarding skepticism about the SIEAL and Energy Treaty: We have added a paragraph explicitly acknowledging that "many analysts remain skeptical about the political and geopolitical feasibility of such an ambitious instrument, given the persistent sovereignty concerns, ideological divergences among governments, and the historically slow pace of institutionalization in Latin American regionalism" [75,108,110]. We then clarify that we view these obstacles as precisely the barriers the quantified costs of inaction are designed to help overcome. (ii) Regarding the word "new": The term has been replaced with "dedicated," and additional language has been added to clarify that the proposed Energy Treaty does not currently exist at the regional level: "akin to a dedicated LA Energy Treaty—an instrument that, to date, does not exist at the regional level, which is precisely what motivates its proposal here." This framing makes clearer why the proposal is novel without implying novelty of the idea itself. The changes are highlighted in the revised manuscript.*

Original	Revised
To seize this critical juncture, we propose a new generation of regional energy policy, headlined by a foundational "Latin American Energy Pact". This high-level political agreement should aim for the phased establishment of a LA Electricity Integration System (SIEAL). A critical deliverable of this Pact should be the development of a coordinated legal corpus, akin to a new LA Energy Treaty. The notion of a regional energy treaty aimed at promoting collaboration and a stable investment environment, especially in South America, has been previously examined, highlighting its capacity to alleviate risks such as resource nationalism and draw essential infrastructure investment.	*To seize this critical juncture, we propose a new generation of regional energy policy, headlined by a foundational "Latin American Energy Pact". This high-level political agreement should aim for the phased establishment of a LA Electricity Integration System (SIEAL). A critical deliverable of this Pact should be the development of a coordinated legal corpus, akin to a dedicated LA Energy Treaty—an instrument that, to date, does not exist at the regional level, which is precisely what motivates its proposal here. The notion of a regional energy treaty aimed at promoting collaboration and a stable investment environment, especially in South America, has been previously examined [74], highlighting its capacity to alleviate risks such as resource nationalism and draw essential infrastructure investment. We acknowledge, however, that many analysts remain skeptical about the political and geopolitical feasibility of such an ambitious instrument, given the persistent sovereignty concerns, ideological divergences among governments, and the historically slow pace of institutionalization in Latin American regionalism [75,108,110]. Rather than dismissing these obstacles, we view them as precisely the barriers that the quantified costs of inaction presented in this study are designed to help overcome. We propose to extend this view to the LA region by a macro-treaty that would aim not to reinvent existing frameworks but to harmonize and ensure compatibility among current bilateral and sub-regional agreements.*

Comment R1.8 - Integration pathways only introduced on p.15/17 (minor)

Reviewer Comment: *Finally, the different integration pathways are only introduced in p.15 and developed in p.17 in the methods section. I would suggest to introduce such pathways from the start of the paper, even in the introduction, for the reader to understand what options the authors are considering.*

AR: *Following the reviewer's recommendation, the three integration pathways (Full Integration, Partial Integration, and Fragmented Systems) are now introduced explicitly at the end of the Introduction section, before the MSA framework is presented. A new paragraph explains these pathways and how they serve as the analytical backbone of the quantitative scenarios, allowing readers to understand the cooperation options under consideration from the outset. The full scenario definitions are retained in the Methods section (Scenario Design for Policy Analysis). The addition is highlighted in the revised manuscript.*

**Original**	**Revised**
Level of Regional Integration: This primary dimension explores pathways ranging from: (a) Full Integration, assuming complete coordination in planning, investment, and dispatch across all 21 modeled countries, with minimal institutional barriers to electricity trade; (2) Partial Integration, simulating cooperation among specific sub-regional blocs (e.g., Andean Community, Southern Cone, SIEPAC), reflecting existing political alliances; and (3) Fragmented Systems (or Status Quo), where each country optimizes its system primarily on a national basis. [Note: these pathways are first presented on p.15 within the Methods section, and not in the introduction or early sections of the paper]	*To assess the implications of different degrees of cooperation, this study evaluates three integration pathways: (1) Full Integration, assuming complete regional coordination in planning, investment, and dispatch across all modeled countries; (2) Partial Integration, simulating cooperation among specific sub-regional blocs (e.g., Andean Community, Southern Cone, SIEPAC), reflecting existing political alliances and plausible intermediate steps; and (3) Fragmented Systems, where each country optimizes its system primarily on a national basis, with limited cross-border coordination beyond existing agreements. These pathways serve as the analytical backbone of the quantitative scenarios presented throughout this study. In turn, they allow us to quantify the material consequences of the political dynamics that the MSA helps interpret, making visible how different degrees of cooperation translate into costs, vulnerabilities, and distributional outcomes across the region. [Full definitions are also retained in the Methods section (Scenario Design for Policy Analysis).]*

Reviewer #2 Comments

General assessment: "Firstly, I'd like to say that your work is scientifically consistent, and, at the same time, very relevant in terms of improving the socioeconomic development of Latin America countries. So, Congratulations!!! The methodology employed ('mixed-methods research design, combining qualitative political analysis grounded in the Multiple Streams Approach with quantitative, long-term energy system expansion modeling') represents the study's main strength. It is a solid methodological approach that ultimately enables the work to achieve a high standard of scientific quality, particularly in the field of energy planning. In this context, the study demonstrates inherent reproducibility, and the quality of the English used appears to be excellent."

Comment R2.1 - Geopolitical analysis needs deeper treatment; suggestion of Harvey's 'accumulation by dispossession' theory

Reviewer Comment: *The central argument of your study, as I understand it, is that the necessary energy integration in Latin America can be accelerated by greater political cohesion and by the urgency of climate emergency, which is indeed a coherent proposition. However, this outcome depends on profound transformations in the institutional, economic, and geopolitical arrangements of the region. I recognize that the methodology you applied, centered on the Multiple Streams Approach (MSA), largely captures the complexity inherent in such an argument. Nevertheless, I believe that some of the premises you adopted would require, so to speak, a lower degree of simplification, particularly with regard to geopolitical aspects, which often depend more on the decisions and interests of other countries-for example, in securing access to the metals required for the energy transition, as well as a broader range of commodities-especially those countries representing the Global North and China.*

I believe that you should improve this analysis, considering, for example, the theory of accumulation by dispossession, developed by David Harvey, which describes how neoliberal capitalism perpetuates primitive accumulation through the forced appropriation of public and common assets, transferring wealth to elites. It involves privatization, financialization, crisis management, and state redistribution, resulting in the loss of collective rights and assets.

This aspect, however, does not imply a failure of the study, nor does it prevent its publication in the prestigious Nature Communications journal.

AR: *We sincerely thank Reviewer #2 for this intellectually rich and constructive suggestion. We agree that the geopolitical analysis in the Discussion section required deeper treatment, and we have substantially expanded it in response. We have added two new paragraphs to the Discussion section that explicitly engage with the geopolitical economy in which Latin American integration is embedded. The first contextualizes the region's position vis-à-vis the Global North and China competing for critical minerals (lithium, copper, nickel, graphite, rare earths) underpinning renewable energy technologies. The second directly draws on Harvey's concept of accumulation by dispossession to interpret the risks of financialization of green infrastructure, privatization of strategic resources, and external control of critical mineral value chains—risks that could transfer the benefits of Latin America's renewable endowments to external actors. We also reference the emerging literature on green colonialism and lithium extraction in Latin America (Dorn, 2022; Gómez Lende, 2022) to support the argument that poorly governed energy-transition projects can reproduce ecological dispossession and territorial inequalities. We then argue that strong multilateral regional governance with explicit benefit-sharing, socio-environmental safeguards, and regionally owned planning mechanisms can serve as a counterweight to these dynamics—ensuring that the region's renewable wealth becomes a collective asset. This framing reinforces the urgency of the governance dimension and connects the MSA analysis to critical political economy theory, as suggested by the reviewer. The additions are highlighted in the revised manuscript.*

**Original**	**Revised**
This study offers a critical and timely contribution not only to the academic literature on energy policy and regional integration but, more pressingly, to the current geopolitical energy praxis in Latin America. We move beyond describing persistent political barriers by providing a novel, actionable framework: combining the Multiple Streams Approach (MSA) with quantitative optimal energy expansion modeling to translate abstract political dynamics-such as historical distrust and sovereignty concerns-into concrete, billion-dollar economic trade-offs. Our analysis indicates that the escalating climate crisis, with its severe impacts on regional energy security, is not merely a "focusing event" triggering conditions to overcome decades of integration policies stagnation.	*This study offers a critical and timely contribution not only to the academic literature on energy policy and regional integration but, more pressingly, to the current geopolitical energy praxis in Latin America. We move beyond describing persistent political barriers by providing a novel, actionable framework: combining the Multiple Streams Approach (MSA) with quantitative optimal energy expansion modeling to translate abstract political dynamics—such as historical distrust and sovereignty concerns—into concrete, billion-dollar economic trade-offs. Our analysis indicates that the escalating climate crisis, with its severe impacts on regional energy security, is not merely a "focusing event," but a catalyst that can help overcome decades of stagnation in regional integration policies [2,12,61]. [NEW PASSAGE] Nevertheless, this optimistic reading must be tempered by a recognition of the broader geopolitical economy in which Latin American integration is embedded. Drawing directly on Harvey's concept of accumulation by dispossession [119], these pressures can be interpreted as part of a broader risk that the financialization of green infrastructure, the privatization of strategic resources, and the external control of critical mineral value chains may transfer a significant share of Latin America's renewable and mineral rents to external actors rather than to regional populations. Recent debates on green colonialism and lithium extraction in Latin America further show that poorly governed energy-transition projects can reproduce territorial inequalities, socio-environmental conflicts, and forms of ecological dispossession [120,121]. Regional power integration, if governed through strong multilateral institutions with explicit benefit-sharing, socio-environmental safeguards, and regionally owned planning mechanisms, can serve as a counterweight to these dynamics.*

Comment R2.2 - Incorrect framing of energy sector emissions (p.2)

Reviewer Comment: *In page 2, it's not 'As one of the leading sources of greenhouse gas emissions, the energy sector...'; in fact, globally, the energy sector is the leading source of GHG emissions, responding for more than 70%. Please correct.*

AR: *The reviewer is absolutely right: globally, the energy sector is the leading source of GHG emissions, accounting for over 70% of the total. The phrase "As one of the leading sources of greenhouse gas emissions" has been corrected to "As the leading source of greenhouse gas emissions*

globally—accounting for over 70% of total GHG emissions." The correction is highlighted in the revised manuscript.

Original	Revised
As one of the leading sources of greenhouse gas emissions, the energy sector must accelerate the replacement of fossil fuels with clean alternatives to reduce emissions and enhance system resilience.	*As the leading source of greenhouse gas emissions globally—accounting for over 70% of total GHG emissions—the energy sector must accelerate the replacement of fossil fuels with clean alternatives to reduce emissions and enhance system resilience [2-8].*

Comment R2.3 - Add a dedicated 'Conclusion' section and emphasize cooperative mechanisms

Reviewer Comment: *I believe that the development of the study itself supports the analyses and conclusions presented. However, I suggest that a section entitled 'Conclusion' should be included, possibly by drawing up material from the excellent section 'Quantitative Analysis: Regional Power System Expansion Model,' as well as from other analytical sections.*

In this sense, I understand that it could also be better emphasized in the conclusions that greater political cohesion can create the necessary environment for the climate emergency to translate into effective action, but it will be necessary to overcome differences in interests and strengthen lasting cooperative mechanisms (or something along those lines).

AR: *A dedicated Conclusion section has been added at the end of the manuscript, following the Discussion section. The Conclusion draws together the main analytical and quantitative findings of the study, explicitly highlighting: (i) the convergence of the three MSA streams; (ii) the key quantitative results (fragmentation increases system costs by up to 7%, integration can reduce annual CO2 emissions by up to 225 MtCO2 by 2045 and unlock savings of up to $32 billion USD per year, geopolitical costs of sovereignty preference range from $4.6–7.9 billion USD annually by 2045); and (iii) the emphasis requested by the reviewer—that greater political cohesion is a necessary but not sufficient condition for effective action, and that durable cooperative mechanisms (risk-sharing instruments, harmonized regulatory frameworks, transparent benefit-distribution arrangements) are indispensable for making integration credible and self-sustaining over time. The Conclusion is highlighted in the revised manuscript.*

Original	Revised
In conclusion, Latin America confronts a pivotal, time-sensitive opportunity to transform its fragmented power systems into a resilient, integrated regional network. The convergence of recent crises has aligned the necessary conditions for bold action. Regional integration offers a viable, evidence-based path forward, but seizing this window requires decisive political leadership, a shared strategic vision, and the collective will to overcome historical barriers, leveraging the now-quantifiable costs of non-cooperation. The time to act is now, before this critical opportunity closes. [Note: the manuscript currently has no dedicated Conclusion section; this is the closing paragraph of the Discussion]	*[DEDICATED CONCLUSION SECTION ADDED]*<br><br>*Latin America confronts a pivotal, time-sensitive opportunity to transform its fragmented power systems into a resilient, integrated regional network. This study has demonstrated, through the combined lens of the Multiple Streams Approach and quantitative power system modeling, that the three necessary conditions for major policy change are currently aligned: a highly salient problem stream driven by climate-induced energy disruptions; a well-evidenced policy stream where regional integration is technically viable and economically superior; and an evolving politics stream signaled by emerging multilateral*

	commitments and renewed institutional momentum. Furthermore, the current global geopolitical juncture—marked by conflicts in Ukraine and the Middle East, and the resulting volatility in fossil fuel availability and costs—acts as a powerful exogenous trigger reinforcing the urgency for Latin America to insulate its energy security through deep regional integration. Quantitatively, our simulations show that continued fragmentation increases total system costs by up to 7%, reduces resilience to hydroclimatic shocks, and concentrates benefits within narrow cooperating blocs. By contrast, full regional integration can reduce annual CO2 emissions by up to 225 MtCO2 by 2045 and unlock economic savings of up to $32 billion USD per year, particularly under conditions of water scarcity. The geopolitical cost of prioritizing national sovereignty over regional synergy ranges from $4.6–7.9 billion USD annually by 2045, providing concrete, evidence-based leverage for negotiators and policy entrepreneurs. However, translating this open policy window into effective and lasting action requires more than technical and economic arguments. Greater political cohesion is a necessary condition for the climate emergency to produce real institutional change, but it will not be sufficient on its own. Overcoming entrenched differences in national interests, historical distrust, and geopolitical tensions demands the deliberate construction of durable cooperative mechanisms—including regional risk-sharing instruments, harmonized regulatory frameworks, and transparent benefit-distribution arrangements—that make integration credible and self-sustaining over time. The time to act is now, before this critical window closes.

Comment R2.4 - Strengthen lasting cooperative mechanisms

Reviewer Comment: *Under these considerations, I believe that the manuscript 'Climate urgency enables political alignment for Latin American power integration' possesses sufficient quality and scientific robustness to be published in the prestigious Nature Communications journal. However, the recommendations I have outlined should first be worked on by the authors. I emphasize the obvious: this work is of excellent quality and does not require any structural improvement. The main point to strengthen is adding more emphasis on how to strengthen lasting cooperative mechanisms, recognizing that greater political cohesion is necessary but not sufficient-differences in interests must be overcome through robust institutional design.*

AR: *In response, we have strengthened the section on cooperative mechanisms in the Discussion in two ways: (i) We have added language specifying that the proposed risk-sharing mechanisms and*

harmonized contracts "must be institutionally embedded rather than ad hoc—designed to persist across electoral cycles and changes in government orientation," directly addressing the reviewer's point that political cohesion alone is insufficient. (ii) We have added that the Regional Master Plan for Interconnections "must incorporate transparent benefit-distribution arrangements that make the gains from integration visible and credible to all participating nations, including those currently most skeptical"—reinforcing the need to overcome differences in national interests through institutional design. (iii) In the new Conclusion section (added in response to Comment R2.3), we have further emphasized that "greater political cohesion is a necessary condition for the climate emergency to produce real institutional change, but it will not be sufficient on its own," and that lasting integration requires "the deliberate construction of durable cooperative mechanisms." The changes are highlighted in the revised manuscript.

**Original**	**Revised**
Further crucial steps include directly addressing the "cost of trust" through regional risk-sharing mechanisms and harmonized contracts to reduce investment uncertainty, particularly for risk-averse nations (Section 1.4.1). Enhanced planning and coordination via a Regional Master Plan for Interconnections, supported by multilateral platforms involving all key stakeholders, is also essential. Finally, activating and empowering diverse policy entrepreneurs across governments, MDBs, academia, and civil society is vital to champion integration and build the broad coalitions needed to overcome institutional inertia.	*Further crucial steps include directly addressing the "cost of trust" through regional risk-sharing mechanisms and harmonized contracts to reduce investment uncertainty, particularly for risk-averse nations (the subsubsection 'Quantified Costs of Political Factors'). These mechanisms must be institutionally embedded rather than ad hoc—designed to persist across electoral cycles and changes in government orientation. Enhanced planning and coordination via a Regional Master Plan for Interconnections, supported by multilateral platforms involving all key stakeholders, is also essential; such a Master Plan must incorporate transparent benefit-distribution arrangements that make the gains from integration visible and credible to all participating nations, including those currently most skeptical. Finally, activating and empowering diverse policy entrepreneurs across governments, MDBs, academia, and civil society is vital to champion integration and build the broad coalitions needed to overcome institutional inertia.*

We sincerely thank the Editor and both Reviewers for their time and constructive engagement with our work. We are confident that these revisions significantly strengthen the manuscript and look forward to the next stage of the review process.

Climate urgency enables political alignment for Latin American power integration

Authors' responses to reviewers

Manuscript ID: NCOMMS-25-74219A | Journal: Nature Communications

General Comment

The authors thank the Editor and both Reviewers once again for their continued guidance through this final round of revisions for the accepted manuscript. We are delighted that the paper has been accepted in principle for publication in Nature Communications. Presented below is a point-by-point reply to the reviewer comments received with the “Final revisions” decision letter of 15 June 2026, with each response labeled “*AR*” (Author’s Response) and preceded by the corresponding reviewer comment. The editorial requirements detailed in the accompanying Author Checklist have been reviewed and completed separately and are summarized in the cover letter.

Reviewer #1 Comments

Reviewer #1 confirmed, both in the "Remarks to the Author" and "Remarks on code availability" fields, that all previous comments and suggestions had been adequately addressed.

Comment R1.1 – Remarks to the Author

Reviewer Comment: *All my comments and suggestions were adequately addressed.*

AR: *We sincerely thank Reviewer #1 for confirming that all comments and suggestions raised during the previous round of review have been adequately addressed in the revised manuscript (see our prior responses to Comments R1.1–R1.8). No further action is required from the authors at this stage.*

Comment R1.2 – Remarks on code availability

Reviewer Comment: *All my comments and suggestions were adequately addressed.*

AR: *We thank Reviewer #1 for confirming that the code availability of the manuscript is also satisfactory. The model code, scenario generation scripts, post-processing routines, visualization scripts, and input files remain publicly available in the permanent Zenodo repository (https://doi.org/10.5281/zenodo.19775192). No further action is required.*

Reviewer #3 Comments

General assessment: "Furthermore, I believe that the methodology employed ('mixed-methods research design, combining qualitative political analysis grounded in the Multiple Streams Approach with quantitative, long-term energy system expansion modeling') represents the study's main strength. It is a solid methodological approach that ultimately enables the work to achieve a high standard of scientific quality, particularly in the field of energy planning. In this context, the study demonstrates inherent reproducibility, and the quality of the English used appears to be excellent."

Comment R3.1 – Geopolitical simplification and the theory of accumulation by dispossession (Harvey)

Reviewer Comment: *The central argument of your study, as I understand it, is that the necessary energy integration in Latin America can be accelerated by greater political cohesion and by the urgency of climate emergency, which is indeed a coherent proposition. However, this outcome depends on profound transformations in the institutional, economic, and geopolitical arrangements of the region. I recognize that the methodology you applied, centered on the Multiple Streams Approach (MSA), largely captures the complexity inherent in such an argument. Nevertheless, I believe that some of the premises you adopted would require, so to speak, a lower degree of simplification, particularly with regard to geopolitical aspects, which often depend more on the decisions and interests of other countries—for example, in securing access to the metals required for the energy transition, as well as a broader range of commodities—especially those countries representing the Global North and China. I believe that you should improve this analysis, considering, for example, the theory of accumulation by dispossession, developed by David Harvey, describes how neoliberal capitalism perpetuates primitive accumulation through the forced appropriation of public and common assets, transferring wealth to elites. It involves privatization, financialization, crisis management, and state redistribution, resulting in the loss of collective rights and assets. This aspect, however, does not imply a failure of the study, nor does it prevent its publication in the prestigious Nature Communications journal.*

AR: *We sincerely thank Reviewer #3 for this comment. We have further refined this passage to more explicitly capture the two dimensions the reviewer highlights: that external competition extends beyond critical minerals to a broader range of commodities and value chains, and that the pace and shape of regional integration depend not only on intra-regional political will but also on the decisions and strategic interests of external actors (Global North and China), who shape financing terms, trade rules, and technology-transfer conditions. These additions are supported by the geopolitical literature already cited in this passage [108,109,111,112] and are highlighted in the revised manuscript below.*

Original	Revised
The region's energy transition is not occurring in a vacuum: the Global North and China are actively competing for preferential access to the critical minerals and commodities—including lithium, copper, nickel, graphite, and rare earth elements—that underpin renewable energy infrastructure, storage, transmission, and electrification technologies [108,109,111,112]. This creates opportunities for industrial development and regional cooperation, but it also exposes the region to external competition for control over resources, infrastructure, financing, and regulatory influence. These dynamics introduce structural tensions that	*"The region's energy transition is not occurring in a vacuum: the Global North and China are actively competing for preferential access not only to critical minerals—including lithium, copper, nickel, graphite, and rare earth elements—**but to a broader range of commodities and associated value chains** that underpin renewable energy infrastructure, storage, transmission, and electrification technologies [108,109,111,112]. **The pace and shape of Latin American integration therefore depend not solely on intra-regional political will, but also on the decisions and strategic interests of these external actors,***

the MSA framework, focused primarily on policy-stream convergence and political coupling, does not fully capture. Drawing directly on Harvey's concept of accumulation by dispossession, these pressures can be interpreted as part of a broader risk that the financialization of green infrastructure, the privatization of strategic resources, and the external control of critical mineral value chains may transfer a significant share of Latin America's renewable and mineral rents to external actors rather than to regional populations [119]. Recent debates on green colonialism and lithium extraction in Latin America further show that poorly governed energy-transition projects can reproduce territorial inequalities, socio-environmental conflicts, and forms of ecological dispossession [120,121]. Regional power integration, if governed through strong multilateral institutions with explicit benefit-sharing, socio-environmental safeguards, and regionally owned planning mechanisms, can serve as a counterweight to these dynamics—ensuring that the region's renewable wealth becomes a collective asset rather than a new frontier for external appropriation. This consideration further reinforces the urgency of building robust, regionally owned governance frameworks as a prerequisite for integration that is both effective and equitable.	***who shape financing terms, trade rules, and technology-transfer conditions.*** *This creates opportunities for industrial development and regional cooperation, but it also exposes the region to external competition for control over resources, infrastructure, financing, and regulatory influence. These dynamics introduce structural tensions that the MSA framework, focused primarily on policy-stream convergence and political coupling, does not fully capture.*<br>*Drawing directly on Harvey's concept of accumulation by dispossession [119], these pressures can be interpreted as part of a broader risk that the financialization of green infrastructure, the privatization of strategic resources, and the external control of critical mineral value chains may transfer a significant share of Latin America's renewable and mineral rents to external actors rather than to regional populations. Recent debates on green colonialism and lithium extraction in Latin America further show that poorly governed energy-transition projects can reproduce territorial inequalities, socio-environmental conflicts, and forms of ecological dispossession [120,121]. Regional power integration, if governed through strong multilateral institutions with explicit benefit-sharing, socio-environmental safeguards, and regionally owned planning mechanisms, can serve as a counterweight to these dynamics—ensuring that the region's renewable wealth becomes a collective asset rather than a new frontier for external appropriation."*

Comment R3.2 – Energy sector as the leading source of GHG emissions (p.2)

Reviewer Comment: *In page 2, it's not "As one of the leading sources of greenhouse gas emissions, the energy sector…", in fact, globally, the energy sector is the leading source of GHF emissions, responding for more than 70%. Please correct.*

AR: *We thank Reviewer #3 for this observation. The same correction was requested by a reviewer during the previous round (Comment R2.2 in our prior response letter) and was implemented at that time: the sentence on page 2 was revised to state that the energy sector is the leading source of GHG emissions globally, accounting for more than 70% of the total. This corrected wording is already part of the accepted manuscript and is reproduced below for the reviewer's reference. No further action is required.*

Original	Revised
As the leading source of greenhouse gas emissions globally—accounting for over 70% of total GHG emissions—the energy sector must accelerate the replacement of fossil fuels with	*[No additional changes are required. This passage was already incorporated during the previous round of revisions and remains unchanged in the current, accepted version of the manuscript.]*

clean alternatives to reduce emissions and enhance system resilience [2-8].	

Comment R3.3 – Inclusion of a dedicated "Conclusion" section

Reviewer Comment: *I believe that the development of the study itself supports the analyses and conclusions presented. However, I suggest that a section entitled "Conclusion" should be included, possibly by drawing up material from the excellent section "Quantitative Analysis: Regional Power System Expansion Model," as well as from other analytical sections. In this sense, I understand that it could also be better emphasized in the conclusions that greater political cohesion can create the necessary environment for the climate emergency to translate into effective action, but it will be necessary to overcome differences in interests and strengthen lasting cooperative mechanisms (or something along those lines).*

AR: *We thank Reviewer #3 for this suggestion. A dedicated Conclusion section was added to the manuscript in response to an identical recommendation made by a reviewer during the previous round (Comment R2.3 in our prior response letter). That section explicitly states that greater political cohesion is a necessary, but not sufficient, condition for the climate emergency to translate into effective institutional action, and that durable cooperative mechanisms are required to overcome differences in national interests — precisely the emphasis requested by the reviewer.*

Separately, in line with the editorial requirements specified in the accompanying Author Checklist for this round, we have removed the "Conclusion" heading, as the main text of Nature Communications articles may only comprise the following sections: Abstract, Introduction, Results, an optional Discussion section (which may optionally be combined with Results), and an optional Methods section. Accordingly, the paragraphs previously presented under the "Conclusion" heading have been retained in full, without any reduction in content, and now form the closing paragraphs of the Discussion section. This ensures that the substantive emphasis requested by Reviewer #3 remains fully present in the manuscript, while complying with the journal's formatting requirements. The main text now contains only Abstract, Introduction, Results, Discussion, and Methods. No further action is required.

**Original**	**Revised**
Latin America confronts a pivotal, time-sensitive opportunity to transform its fragmented power systems into a resilient, integrated regional network. This study has demonstrated, through the combined lens of the Multiple Streams Approach and quantitative power system modeling, that the three necessary conditions for major policy change are currently aligned: a highly salient problem stream driven by climate-induced energy disruptions; a well-evidenced policy stream where regional integration is technically viable and economically superior; and an evolving politics stream signaled by emerging multilateral commitments and renewed institutional momentum. Furthermore, the current global geopolitical juncture—marked by conflicts in Ukraine and the Middle East, and the resulting volatility in fossil fuel availability and costs—acts as a powerful exogenous trigger. This macro-level instability only reinforces the urgency for Latin America to insulate its energy security through	*[No additional changes are required. This passage was already incorporated during the previous round of revisions and remains unchanged in the current, accepted version of the manuscript.]*

deep regional integration, thereby accelerating the opening of this policy window. Quantitatively, our simulations show that continued fragmentation increases total system costs by up to 7%, reduces resilience to hydroclimatic shocks, and concentrates benefits within narrow cooperating blocs. By contrast, full regional integration can reduce annual CO2 emissions by up to 225 MtCO2 by 2045 and unlock economic savings of up to $32 billion USD per year, particularly under conditions of water scarcity. The geopolitical cost of prioritizing national sovereignty over regional synergy ranges from $4.6–7.9 billion USD annually by 2045, providing concrete, evidence-based leverage for negotiators and policy entrepreneurs. However, translating this open policy window into effective and lasting action requires more than technical and economic arguments. Greater political cohesion is a necessary condition for the climate emergency to produce real institutional change, but it will not be sufficient on its own. Overcoming entrenched differences in national interests, historical distrust, and geopolitical tensions demands the deliberate construction of durable cooperative mechanisms—including regional risk-sharing instruments, harmonized regulatory frameworks, and transparent benefit-distribution arrangements—that make integration credible and self-sustaining over time. Without these institutional foundations, even the most compelling cost arguments risk remaining rhetorical. The time to act is now, before this critical window closes. Bold political leadership, a shared strategic vision, and the collective will to build lasting cooperative institutions represent the final, indispensable ingredients for securing Latin America's integrated, decarbonized energy future.	

Comment R3.4 – Remarks on code availability (duplicate submission)

Reviewer Comment: *[Reviewer #3 reproduced the same review text quoted above — including the comments addressed in R3.1–R3.3 — verbatim in the "Remarks on code availability" field of the review form, followed by the same closing statement of approval.]*

AR: *We thank Reviewer #3 for this confirmation. As the text submitted in this field is identical to the "Remarks to the Author" field, our responses to Comments R3.1, R3.2, and R3.3 above apply equally here. We also confirm that the code availability statement and the Zenodo repository (https://doi.org/10.5281/zenodo.19775192) containing the model code, scenario generation scripts, post-processing routines, visualization scripts, and input files remain complete and publicly accessible, consistent with the reviewer's approval.*

Closing remark by Reviewer #3 (reproduced verbatim from both the "Remarks to the Author" and "Remarks on code availability" fields, dated 7 June 2026): "I believe you have carefully reviewed my comments, and I believe you have indeed made an effort to implement, in one way or another, all of my recommendations; in this context, I approve the publication of the work in the prestigious journal Nature Communications. Congratulations, dear Author!!!"

AR: We are deeply grateful to Reviewer #3 for this confirmation and for the recommendation of publication, and to both Reviewers and the Editor for their valuable guidance throughout the review process.
